# Gaussian Process Regression for Materials and Molecules

**DOI:** 10.1021/acs.chemrev.1c00022

**Published:** 2021-08-16

**Authors:** Volker L. Deringer, Albert P. Bartók, Noam Bernstein, David M. Wilkins, Michele Ceriotti, Gábor Csányi

**Affiliations:** † Department of Chemistry, Inorganic Chemistry Laboratory, University of Oxford, Oxford OX1 3QR, United Kingdom; ‡ Department of Physics and Warwick Centre for Predictive Modelling, School of Engineering, University of Warwick, Coventry CV4 7AL, United Kingdom; § Center for Computational Materials Science, U.S. Naval Research Laboratory, Washington D.C. 20375, United States; ∥ Atomistic Simulation Centre, School of Mathematics and Physics, Queen’s University Belfast, Belfast BT7 1NN, Northern Ireland, United Kingdom; ⊥ Laboratory of Computational Science and Modeling, IMX, École Polytechnique Fédérale de Lausanne, Lausanne 1015, Switzerland; # National Centre for Computational Design and Discovery of Novel Materials (MARVEL), École Polytechnique Fédérale de Lausanne, Lausanne, Switzerland; 7 Engineering Laboratory, University of Cambridge, Cambridge CB2 1PZ, United Kingdom

## Abstract

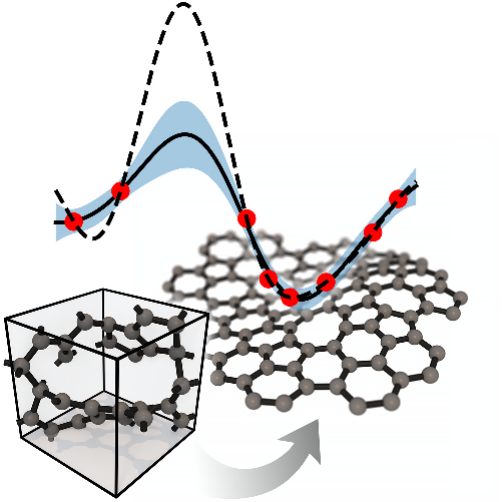

We provide an introduction to Gaussian process regression (GPR) machine-learning methods in computational materials science and chemistry. The focus of the present review is on the regression of atomistic properties: in particular, on the construction of interatomic potentials, or force fields, in the Gaussian Approximation Potential (GAP) framework; beyond this, we also discuss the fitting of arbitrary scalar, vectorial, and tensorial quantities. Methodological aspects of reference data generation, representation, and regression, as well as the question of how a data-driven model may be validated, are reviewed and critically discussed. A survey of applications to a variety of research questions in chemistry and materials science illustrates the rapid growth in the field. A vision is outlined for the development of the methodology in the years to come.

## 1 Introduction

At the heart of chemistry is the need to understand the nature, transformations, and macroscopic effects of atomistic structure. This is true for *materials*—crystals, glasses, nanostructures, composites—as well as for *molecules*, from the simplest industrial feedstocks to entire proteins. And with the often-quoted role of chemistry as the “central science”,^1,2^ its emphasis on atomistic understanding has a bearing on many neighboring disciplines: candidate drug molecules are made by synthetic chemists based on an atomic-level knowledge of reaction mechanisms; functional materials for technological applications are characterized on a range of length scales, which begins with increasingly accurate information about where exactly the atoms are located relative to one another in three-dimensional space.

Research progress in structural chemistry has largely been driven by advances in experimental characterization techniques, from landmark studies in X-ray and neutron crystallography to novel electron microscopy techniques which make it possible to visualize individual atoms directly. Complementing these new developments, detailed and realistic structural insight is also increasingly gained from computer simulations. Today, chemists (together with materials scientists) are heavy users of large-scale supercomputing facilities, and the computationally guided discovery of previously unknown molecules and materials has come within reach.^3−8^

Computations based on the quantum mechanics of electronic structure, currently most commonly within the framework of density-functional theory (DFT), are widely used to study structures of molecules and materials and to predict a range of atomic-scale properties.^9−11^ Two approaches are of note here. One is the prediction of atomically resolved physical quantities, e.g., isotropic chemical shifts, δ_iso_, that can be used to simulate NMR spectra with a large degree of realism^12^—thereby making it possible to corroborate or falsify a candidate structural model or to deconvolute experimentally measured spectra. The other central task is the determination of atomistic structure itself, achieved through molecular dynamics (MD), structural optimization, and other quantum-mechanically driven techniques. Many implementations of DFT exist and are widely used, and their consistency has been demonstrated in a comprehensive community-wide exercise.^13^

Electronic-structure computations are expensive, in terms of both their absolute resource requirements and their scaling behavior with the number of atoms, *N*. For DFT, the scaling is typically  in the most common implementations; see ref (14) for the current status of a linear-scaling implementation. Routine use is therefore limited to a few thousand atoms at most for DFT single-point evaluations, to a few hundred atoms for DFT-driven “ab initio” MD, and to even fewer for high-level wave function theory methods such as coupled cluster (CC) theory or quantum Monte Carlo (QMC). The latter techniques offer an accuracy far beyond standard DFT, and they are beginning to become accessible not only for isolated molecules but also for condensed phases. However, running MD with these methods requires substantial effort and is currently largely limited to proof-of-principle simulations.^15−17^ For studies that predict atomistic properties, such as NMR shifts, derived from the wave function, a new electronic-structure computation has to be carried out every time a new structure is considered, again incurring large computational expense.

In the past decade, machine learning (ML) techniques have become a popular alternative, aiming to make the same type of predictions using an approximate or surrogate model, while requiring only a small fraction of the computational costs. There is practical interest in being able to access much more realistic descriptions of structurally complex systems (e.g., disordered and amorphous phases) than currently feasible, as well as a wider chemical space (e.g., scanning large databases of candidate materials rather than just a few selected ones). There is also a fundamental interest in the question of how one might “teach” chemical and physical properties to a computer algorithm which is inherently chemically agnostic and in the relationship of established chemical rules with the outcome of purely data-driven techniques.^19^ We may direct the reader to high-level overviews of ML methods in the physical sciences by Butler et al.,^20^ Himanen et al.,^21^ and Batra et al.,^22^ to more detailed discussions of various technical aspects,^23−26^ and to a physics-oriented review that places materials science in the context of many other topics for which ML is currently being used.^27^

The use of ML in computational chemistry, materials science, and also condensed-matter physics is often focused on the regression (fitting) of atomic properties, that is, the functional dependence of a given quantity on the local structural environment. For the case of force fields and interatomic potentials, there are a number of general overview articles^28−31^ and examples of recent benchmark studies.^32,33^ There are also specialized articles that offer more detailed introductions.^34−38^

**Figure 1:**
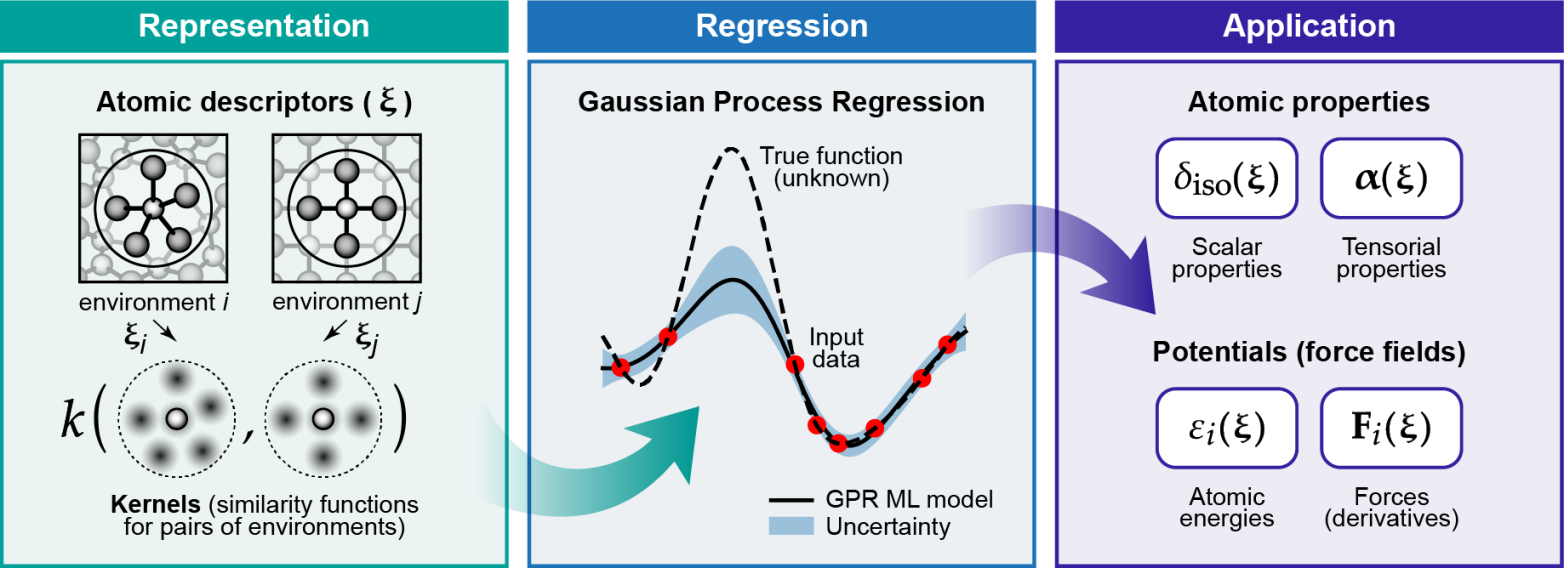
Overview of central concepts in Gaussian process regression (GPR) machine-learning models of atomistic properties. Left: The models discussed in the present review are based on atomistic structure, and therefore, they require a suitable representation of atomic environments up to a cutoff. The neighborhood is “encoded” using a descriptor vector, **ξ**, and a kernel function, *k*, which is used to evaluate the similarity of two atomic environments. Center: In the regression task, the goal is to infer an unknown function from a limited number of observations or input data (section 2). The result, in GPR, is a function with quantifiable uncertainty. Right: Applications of GPR. There are two main classes within the scope of the present review. The first class of applications is the fitting of atomic properties (section 3): these can be scalar, such as the isotropic chemical shift in NMR, δ_iso_, or vectors or higher-order tensors, such as the polarizability, **α**. The second class of applications is the construction of interatomic potentials or force fields (section 4), which describe atomic energies, *ε*_*i*_ , as well as interatomic forces, **F_*i*_**. All these properties are fitted as functions of the descriptor, **ξ**. The drawings on the left are adapted from ref (18). Adapted by permission of The Royal Society of Chemistry. Copyright 2020 The Royal Society of Chemistry.

In the present work, we review the application of Gaussian process regression (GPR) to computational chemistry, with an emphasis on the development of the methodology over the past decade. Figure 1 provides an overview of the central concepts. Given early successes, there is significant emphasis on the construction of accurate, linear-scaling force-field models and the new chemical and physical insights that can be gained by using them. We also survey, more broadly, methodology and emerging applications concerning the “learning” of general atomistic properties that are of interest for chemical and materials research. Quantum-mechanical properties, including the eletronic energy, are inherently nonlocal, but the degree to which local approximations, taking account of the immediate neighborhood of an atom, can be used will be of central importance. It is hoped that the present work—indeed the entire thematic issue in which it appears—will provide guidance and inspiration for research in this quickly evolving field and that it will help advance the transition of the methodology from relatively specialized to much more widely used.

## 2 Gaussian Process Regression

We begin this review article with a brief general introduction to the basic principles of GPR. The present section is not yet concerned with applications, but rather provides a discussion of the underlying mathematical concepts and motivates them for modeling functions in the context of chemistry and physics, as a preparation for subsequent sections of this review. A glossary of the most important terms is provided in Table 1.

Inferring a continuous function from a set of individual (observed or computed) data points is a common task in scientific research. Depending on the prior knowledge of the process that underlies the observations, a wide range of approaches are available. If there exists a plausible model that can be translated to a closed functional formula, parametric fitting is most suitable, as limited data are often sufficient to estimate the unknown parameters. Examples include the interaction of real (nonideal) gas particles, the Arrhenius equation, or, closer to the topic of the present review, the *r*^–6^ decay of the long-range tail of the van der Waals dispersion interaction.

In practice, not all processes can be modeled well by simple expressions. Structure–property relations, kinetics of biomolecular reactions, and quantum many-body interactions are examples of observable outcomes that depend on input variables in a complex, not easily separable way, because of the presence of hidden variables. Instead of trying to understand this dependence analytically, one may set out to describe it *purely* based on existing data and observations. Interpolation and regression techniques provide tools to fill in the space between data points, resulting in a continuous function representation which, once established, can be used in further work. Linear interpolation and cubic splines are widely used examples of these methods, but they are limited to low-dimensional data and cases where there is little noise in the observations. With more than a few variables, it becomes exponentially more difficult to collect sufficient data for the uniform coverage that is required by these methods. As interpolation techniques are inherently local, noise in observations is not averaged out over a larger domain, meaning that these approaches tend to be less tolerant to uncertainty in the data.

From the practitioner’s point of view, GPR is a nonlinear, nonparametric regression tool, useful for interpolating between data points scattered in a high-dimensional input space. It is based on Bayesian probability theory and has very close connections to other regression techniques, such as kernel ridge regression (KRR) and linear regression with radial basis functions. In the following, we will discuss how these methods are related.

Nonparametric regression does not assume an *ansatz* or a closed functional form, nor does it try to explain the process underlying the data using theoretical considerations. Instead, we rely on a large amount of data to fit a flexible function with which predictions can be made; this is what we call “machine learning”.

GPR provides a solution to the modeling problem such that the locality of the interpolation may be explicitly and quantitatively controlled, by encoding it in the *a priori* assumption of smoothness of the underlying function. To introduce GPR, we consider a smooth, regular function, *y*(**x**), which takes a *d*-dimensional vector as input and maps it onto a single scalar value:
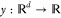
1We do not know the functional form of *y*, but we have made *N* independent observations, *y*_*n*_ , of its value at the locations **x**_*n*_ , resulting in a *dataset*,
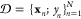
2We can consider the observations, *y*_*n*_ to be *samples* of *y*(**x**) at the given location, which may contain observation noise. The goal is now to use these data values to create an estimator that can predict the continuous function *y*(**x**) at *arbitrary* locations **x** and also to quantify the uncertainty (“expected error”) of this prediction.

There are two equivalent approaches to deriving the GPR framework: the *weight-space* and the *function-space* views, each highlighting somewhat different aspects of the fitting process.^39^ We provide both derivations in the following.

### 2.1 Weight-Space View of GPR

**Figure 2:**
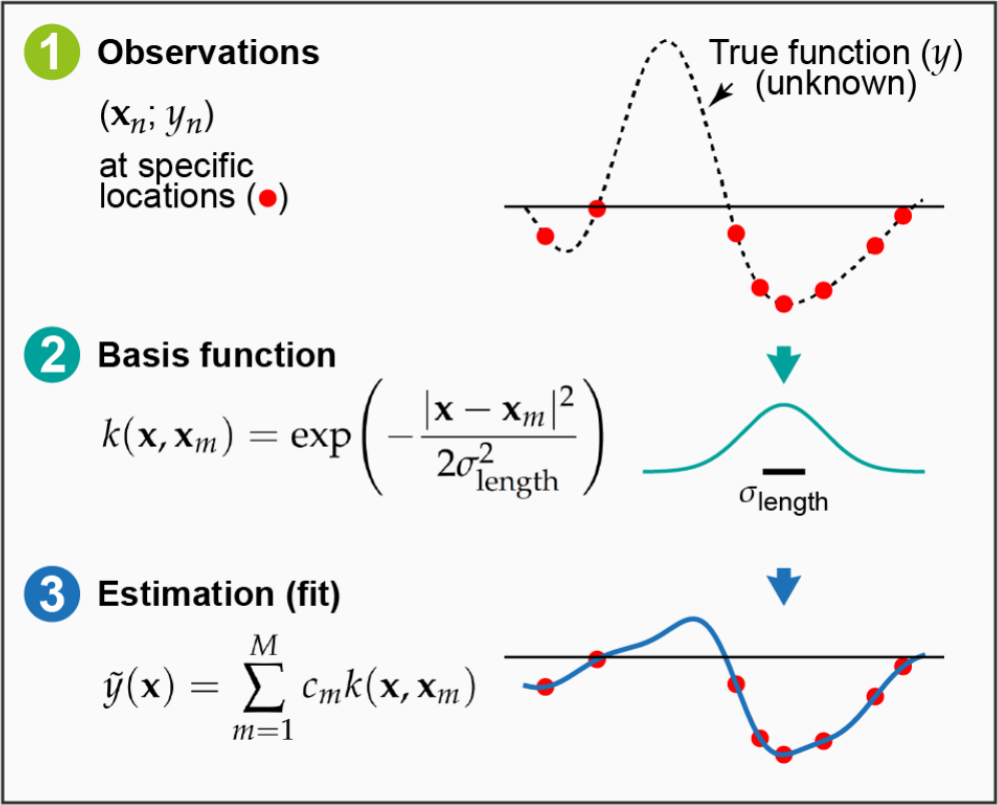
Basic elements of GPR as discussed in the present section: (1) observations of an unknown function at a number of locations; (2) basis functions (only one of them shown for clarity), centered at the data locations; (3) an estimation, , defined by the set of coefficients, *c*_*m*_, and the corresponding basis functions; this is the prediction of the GPR model.

In the weight-space view of GPR, which is also the one most closely aligned with the usual exposition of kernel ridge regression, we approximate *y*(**x**) by (**x**), defined as a linear combination of *M* basis functions (Figure 2):
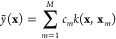
3where the basis functions, *k*, are placed at arbitrary locations in the input space, **x**_*m*_ , comprising what we refer to as the *representative set*, {**x**_*m*_}_*m*=1_^*M*^ (sometimes also called the “active” or “sparse” set), and *c*_*m*_ are coefficients or weights. At this stage, we do not need to specify the actual functional form of *k*; we only need to know that  describes the similarity between the function at two arbitrary locations, , that the function is symmetric to swapping its arguments, viz. *k*(**x**, **x**′) = *k*(**x**′, **x**), and that it is positive semidefinite. The kernel function is positive semidefinite when, given an arbitrary set of inputs {**x**_*n*_}, the matrix built from *k*(**x**_*n*_, **x**_*n*′_) is positive semidefinite.

**Table 1:** Glossary of Technical Terms and Concepts Relevant to GPR^a^

**Covariance**	A measure for the strength of statistical correlation between two data values, *y*(**x**) and *y*(**x′**), usually expressed as a function of the distance between **x** and **x′**. Uncorrelated data lead to zero covariance.
**Descriptor**	In the context of regression, descriptors (sometimes called “features”) encode the independent variables into a vector, **x**, on which the modeled variable, *y*, depends.
**Hyperparameter**	A global parameter of an ML model that controls the behavior of the fit. Distinct from the potentially very large number of “free parameters” that are determined when the model is fitted to the data. Hyperparameters are estimated from experience or iteratively optimized using data.
**Kernel**	A similarity measure between two data points, normally denoted *k*(**x**, **x**′). Used to construct models of covariance.
**Overfitting**	A fit that is accurate for the input data but has uncontrolled errors elsewhere (typically because it has not been regularized appropriately).
**Prior**	A formal quantification, as a probability distribution, of our initial knowledge or assumption about the behavior of the model, before the model is fitted to any data.
**Regularity**	Here, we take a function to be regular if all of its derivatives are bounded by moderate bounds. Loosely interchangeable with “degree of smoothness”.
**Regularization**	Techniques to enforce the regularity of fitted functions. In the context of GPR, this is achieved by penalizing solutions which have large basis coefficient values. The magnitude of the regularization may be taken to correspond to the “expected error” of the fit.
**Sparsity**	In the context of GPR, a sparse model is one in which there are far fewer kernel basis functions than input data points, and the locations of these basis functions (which we call the *representative* set) need not coincide with the input data locations.
**Underfitting**	A fit that does not reach the accuracy, on neither the training nor the test data, that would be possible to achieve by a better choice of hyperparameters.

a These definitions do not yet refer to physical properties, but they will be used in subsequent sections. For a comprehensive introduction to GPR, we refer the reader to ref (39).

**Figure 3:**
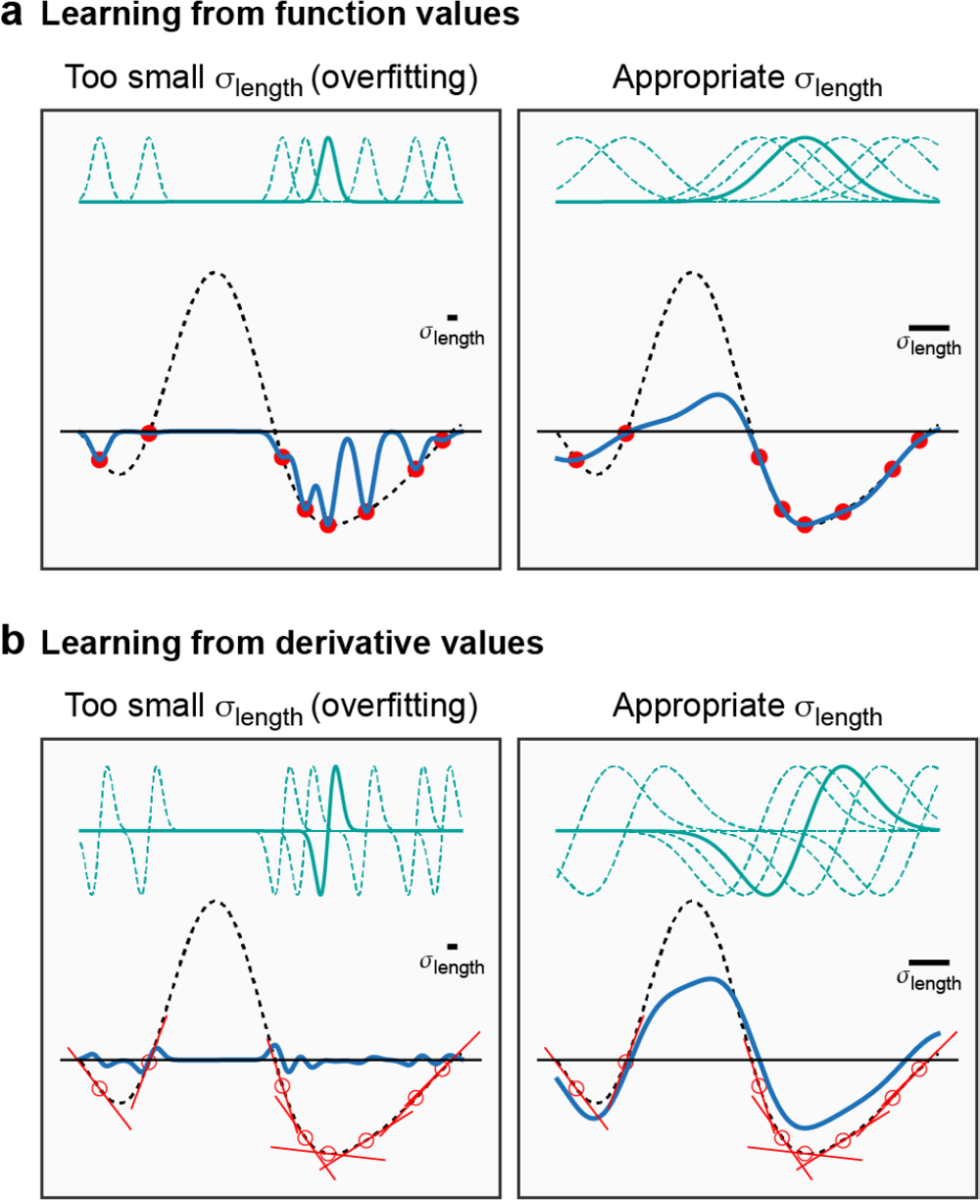
Effect of the kernel length scale on the GPR fit for different types of input data. (a) Learning from function value observations. We illustrate the effect of using a small (left) or larger (right) hyperparameter associated with the correlation length scale (represented by a solid bar in each panel) on the GPR models (solid black line). Basis functions (teal dashed lines—one is highlighted as solid for clarity), centered on data points (red circles) sampled from the target function (black dashed line), are also shown. (b) Learning from derivative values (section 2.4). Data points are represented by red points and derivatives by red sticks: in this example, the data values themselves, i.e. the {*y*_*n*_}, are not included in the fits. For all fits in this figure, the regularizer was very small, just large enough to ensure that a stable numerical solution to the linear least-squares problem can be obtained.

Although the form of the kernel function does not matter *in principle*, the practical success or failure of a GPR model will depend to a large extent on choosing the appropriate kernel. Figure 3a demonstrates this using the example of the Gaussian kernel which includes a length scale hyperparameter, σ_length_ (defined in Figure 2). In fact, this kernel is a universal approximator for any setting of the length scale, but choosing an inappropriate length scale will result in very slow convergence as a function of the number of training data points.

The fitting of the GPR model to the data is accomplished by finding the coefficients **c** = (*c*_1_, ..., *c*_*M*_) that minimize the loss function,
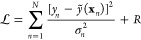
4where *R* is a regularization term and the relative importance of individual data points is controlled by the parameters *σ*_*n*_. In GPR, the Tikhonov regularization is used, defined as
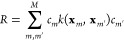
5Two objectives are included in the loss function that is defined in eq 4. The first term is designed to achieve a close fit to the data points. However, this term alone would lead to overfitting because of the large flexibility of the functional form, and it is therefore controlled by the second term, the regularization, which forces the coefficients to remain small. The collection of parameters {σ_*n*_}_*n*=1_^*N*^ (together with the length scale hyperparameter) adjusts the balance between accurately reproducing the fitting data points and the overall smoothness of the estimator.

Crucially, the coefficients in this regularization term are also weighted by the corresponding kernel elements, a relation that can be understood when formally deriving GPR from the properties of the reproducing kernel Hilbert space (RKHS), which we discuss below.^39,40^Equation 4 is often written as
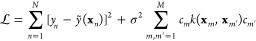
6using a uniform *σ*_*n*_ = σ parameter for all data points, but we later exploit the ability to express the reliability of each data point individually. Because in this form, σ multiplies the Tikhonov regularizer, most practitioners identify σ with the regularization “strength” or “magnitude”. Using this definition, we can rewrite the loss function in matrix form:
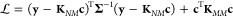
7where the matrix elements are defined as
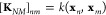
8and **y** = (*y*_1_, ..., *y*_*N*_). Recall that *N* indicates the number of data points in  and *M* indicates the number of representative points, respectively. Our notation emphasizes the dimensions of the various kernel matrices in the subscript and implies that **K**_*NM*_^T^ ≡ **K**_*MN*_ because the kernel function is symmetric. In eq 7, **Σ** is a diagonal matrix of size *N*, collecting all the *σ*_*n*_ values, with **Σ**_*nn*_ = σ_*n*_^2^. To minimize , we differentiate eq 7 with respect to *c*_*m*_ for all *m* and then search for solutions that satisfy
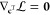
9and we obtain
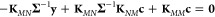
10

**Figure 4:**
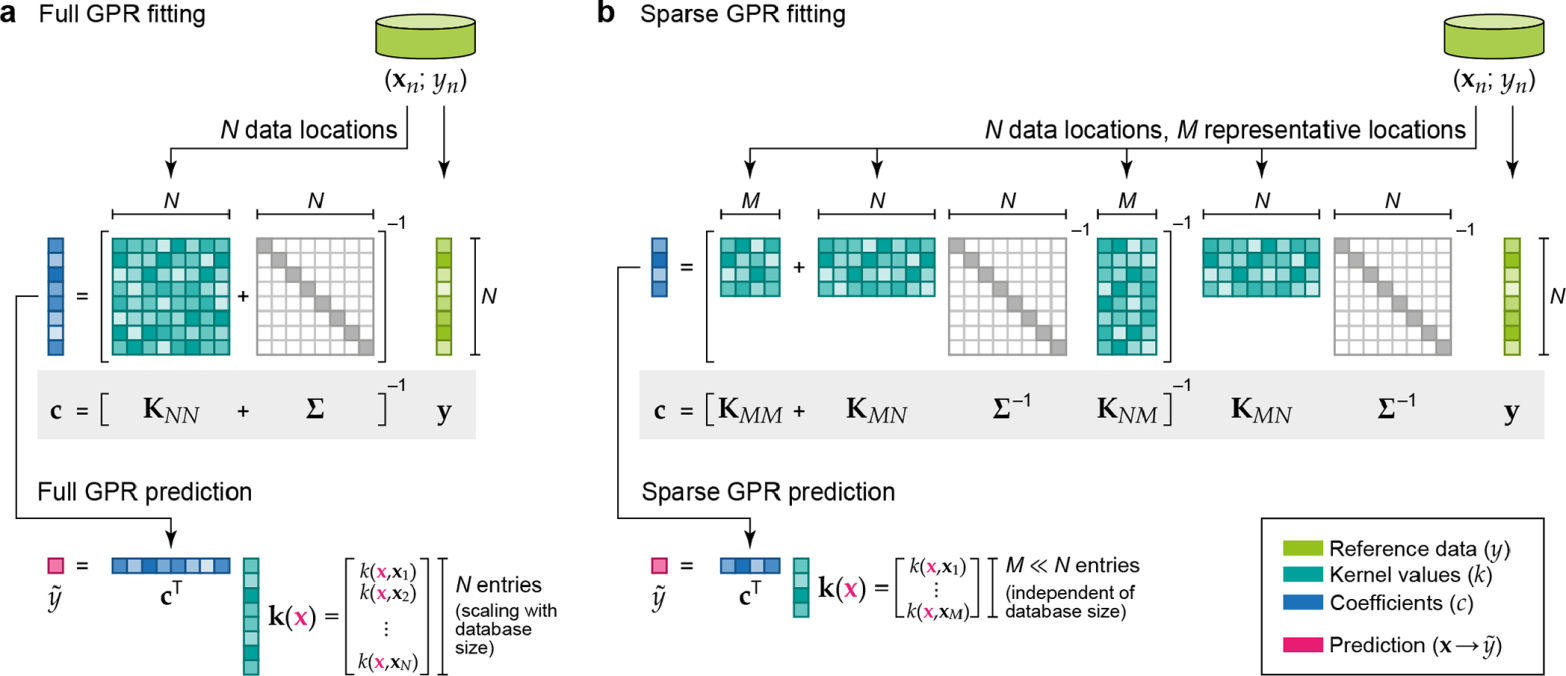
Visualization of the matrix equations that define the fitting of full (eq 14) and sparse (eq 11) GPR models, and the way they are used for prediction. (a) The reference database consists of entries {**x**_*n*_; *y*_*n*_}; the data labels *y*_1_ to *y*_*n*_ are collected in the vector **y** (light green); the data locations **x**_1_ to **x**_*N*_ are used to construct the kernel matrix, **K**, of size *N* × *N* (teal). The regularizer, **Σ**, is shown as a light gray diagonal matrix. By solving the linear problem, the coefficient vector **c** (blue) is computed, and this can be used to make a prediction at a new location, (**x**) (eq 12), the cost of which scales with the number of data locations, *N*. (b) In sparse GPR, the full data vector **y** is used as well, but now *M* representative (“sparse”) locations are chosen, with *M* ≪ *N*. The coefficient vector is therefore of length *M*, and the cost of prediction is now independent of *N*.

Rearranging gives an analytical expression for the coefficients,
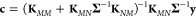
11and once these coefficients have been determined, the prediction at a new location **x** is evaluated using eq 3, which in matrix notation is
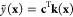
12where a shorthand notation **k**(**x**) is introduced for the vector of kernel values at the prediction location (**x**) and the set of representative points ({**x**_*m*_}),
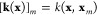
13When the number and locations of the representative points are set to coincide with the input data points, a case to which we refer as “full GPR”, we have *M* = *N*, and the expression for the coefficients simplifies to
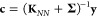
14We note that the expression in eq 14, together with eq 3, is formally equivalent to kernel ridge regression (KRR), which is also based on the solution of the least-squares problem using Tikhonov regularization.^41^ Full GPR becomes expensive for large datasets, because the computational time required to generate the approximation scales with the cube of the dataset size, , and the memory requirement scales as , at least when direct dense linear algebra is used to solve eq 14. While iterative solvers, which are ubiquitous in ML generally, might reduce this scaling, they are not widely employed in the context of GPR/KRR. In our applications, detailed in the rest of this review, we use relatively few representative points, i.e. *M* ≪ *N*, and we refer to this regime as “sparse GPR”, following the Gaussian process literature.^42,43^ The matrix equations that specify both the full and the sparse GPR fits are visualized in Figure 4. More details on how we select representative points in practice will be given in section 4.3.

### 2.2 Function-Space View of GPR

The function-space view is an alternative way of deriving, defining, and understanding GPR/KRR. Again, we aim to estimate an unknown function which we can sample at specified locations, resulting in the dataset , and we consider estimators of the form
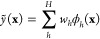
15where ϕ are *fixed*, and for-now unspecified, basis functions. It is important to emphasize that even though eqs 3 and 15 are formally similar, the basis functions *ϕ*_*h*_ are not equivalent to the kernel function *k* (their relationship is shown below), nor are the coefficients **c** equivalent to **w**. Whereas in the weight-space view, the kernel basis functions are placed on the representative set of points **x**_*m*_, which typically (but not necessarily) coincide with data points, the fixed basis functions here are independent of the data and serve purely as a framework to define a probability distribution of functions.

The function  is determined by the coefficients, **w** = (*w*_1_, *w*_2_, ...), which are drawn from independent, identically distributed Gaussian probability distributions,
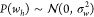
16leading not to a single estimate of  but to a distribution of estimators, which corresponds to a Gaussian prior and is commonly called a Gaussian process (GP). For these generalized estimators, the covariance of two estimator *values* at the locations **x** and **x**′ is
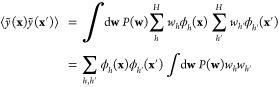
17With the information from eq 16, the integral evaluates to σ_*w*_^2^δ_*hh*′_, and then we have
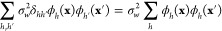
18The sum over the basis functions in the last expression is used to define a kernel function, *k*, as
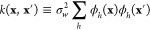
19This definition makes it clear why the kernel function needs to be positive semidefinite: it has the structure of a Gram matrix, i.e. a matrix of scalar products. For coinciding arguments (**x** = **x**′), the value of the kernel function corresponds to a variance.

Each function value in the dataset is taken to incorporate observation noise, *y*_*n*_ = *y*(**x**_*n*_) + ϵ, where ϵ is a random variable, independent for each data point and identically distributed, drawn from a Gaussian distribution with zero mean and variance σ^2^. It follows that the covariance of any two actual function observations in the dataset is given by
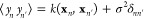
20The probability distribution of all the observations **y** = (*y*_1_, ..., *y*_*N*_) is therefore a multivariate Gaussian with zero mean and covariance of **K**_*NN*_ + σ^2^**I**, written as
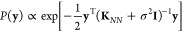
21Note that, for convenience in the derivation, we assume that the mean of the prior distribution of functions is zero, but very often there is a good prior guess for the mean of the function, in which case it is straightforward to modify the distribution—or simply to subtract the prior mean from the observed function values before fitting, to be added back on after prediction.

Function estimation based on the data now proceeds by fixing the *N* data locations and values and considering the probability distribution of a new function value, *y*_*N*__+1_, observed at a new location, **x**_*N*+1_. Bayes’ rule gives this distribution as a conditional probability in terms of the old (previous) observations and the joint distribution of the old and new observations,
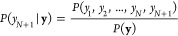
22After substituting eq 21 into eq 22 (using it for both the numerator and denominator appropriately), and some algebraic manipulation,^44^ we find that the distribution of *y*(**x**_*N*+1_) is also Gaussian, and we can express its mean and variance as
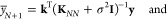
23
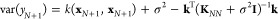
24where we again use **k** for the vector of covariances (kernel values) evaluated between the new data location and the set of *N* previous ones,
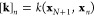
25It is interesting to note that the GP variance estimate is formally independent of the training function values: the expression in eq 24 depends solely on the location of data points but not on the data values **y**. However, if the model hyperparameters are optimized either by maximizing the marginal likelihood or by cross-validation (see below), then the variance estimates do implicitly depend on training function values through this optimization.

The fact that both the estimators in eqs 23 and 24 only depend explicitly on the kernel function, *k*, and not on the basis functions, *ϕ*_*h*_, shows that a GP may be defined by its kernel, without ever specifying the underlying basis set (although it is possible to determine the corresponding basis set from any given kernel). Recall that the meaning of the kernel function is the covariance of data values (eq 19) and is thus regarded as a measure of similarity between data points. This route to specifying a basis for modeling nonlinear functions is often referred to as the “kernel trick”.

Note that the combination of eqs 3 and 14 defining GPR in the weight-space view is equivalent to the result of the function-space view in eq 23. This equivalence reveals that the magnitude of the regularization term in the weight-space view, σ^2^ in eq 6, is the same as the variance of the Gaussian noise model on the function observations (cf. eq 20). We can use this to understand regularization from a new perspective: it is the expression of uncertainty of our observations and naturally leads to a model with an imperfect fit to the data.

Notable kernels include the Gaussian, or squared exponential, kernel (the latter name is in common use to emphasize the distinction between the form of the kernel function and the multivariate Gaussian distributions that underlie the entire GPR framework),
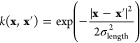
26parametrized by the spatial length scale, σ_length_.^44^ The linear, or dot-product, kernel is defined as
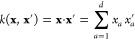
27where *x*_*a*_ = [**x**]_*a*_ are the elements of the *d*-dimensional input vector **x**. Substituting this kernel definition into eq 3 gives the prediction formula,
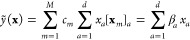
28which shows that using the linear kernel in GPR is equivalent to performing regularized linear regression, with coefficients given by
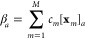
29It follows that the basis functions corresponding to the dot-product kernel are simply *M* functions that each pick out one element of the data vector {**x**_*m*_}_*m*=1_^*M*^. Finally, the polynomial kernel is
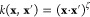
30and expressing the prediction formula explicitly reveals that the basis functions are outer products of the elements of the data vectors. For ζ = 2, for example, we obtain the expression
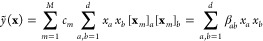
31that corresponds to a polynomial basis with a degree of ζ = 2.^45^

### 2.3 Explicit Construction of the Reproducing Kernel Hilbert Space

It is instructive to see how the function-space view of GPR arises from an explicit construction of an approximation of the RKHS.^46^ Consider the kernel matrix that is computed for the representative set of data points, **K**_*MM*_, and its eigenvalue decomposition which is given by
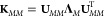
32Because the kernel is positive semidefinite, the eigenvalues, Λ_*i*_, are greater than or equal to zero, and it is possible to compute the *feature matrix*,
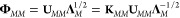
33such that
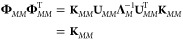
34The definition in eq 33 corresponds to performing a kernel principal component analysis (KPCA)^47^ without discarding any of the resulting components and is consistent with the introduction of an explicit function-space model, as follows. The elements of a feature vector, **ϕ**, associated with an arbitrary input point, **x**, are given by
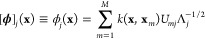
35where the sum runs over all *M* representative points and the number of features is the same; that is, the index *j* takes values from 1 to *M*. For any pair of locations *within the representative set*, we have
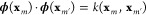
36which corresponds to the definition of the kernel in terms of a scalar product in the RKHS, as given in eq 19. For arbitrary pairs of locations that are not included in the representative set, the above expression is only an approximation of the kernel, which can be improved by enlarging *M*.

This point of view also makes it possible to directly derive the Nyström form of sparse GPR,^48^ by considering it as ridge regression in the RKHS defined by the representative points. The feature matrix associated with a set of *N* points is **Φ**_*NM*_ = **K**_*NM*_**U**_*MM*_**Λ**_*M*_^–1/2^. This expression may be regarded as an *approximate* decomposition of the full kernel matrix **K**_*NN*_ ≈ **Φ**_*NM*_**Φ***_NM_^T^*. The resulting regularized linear regression weights are
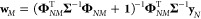
37and the predictions are given by **ϕ**(**x**)·**w**_*M*_. By substituting for the features, **ϕ**, the definition in terms of the eigendecomposition of **K**_*MM*_ (eq 35), we obtain the model predictions,
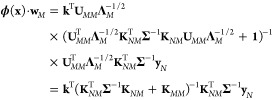
38This is the same as eq 11, revealing how the abstract function-space derivation can be formulated as a matrix approximation problem and more generally how kernel methods can be seen as simultaneously addressing the problem of building a data-adapted feature space and performing linear regression in it.

### 2.4 GPR Based on Linear Functional Observations

In later sections, we will need to use the GPR formalism to estimate functions whose value cannot be directly observed. This is the case for fitting an atomic energy function (using the neighbor environment of an atom as the input) to data from quantum-mechanical electronic-structure computations, which yield the system’s *total* energy, not individual atomic energies, and atomic forces and stresses, which are derivatives of this total energy with respect to the atomic positions and the lattice deformation, respectively. It is therefore useful to consider this problem in the abstract: estimating a function when it is not possible to directly observe values of a function, but we have access to derived properties. The formalism that follows was introduced in ref (49) for modeling materials, which itself builds on ref (50) that discusses learning a function from its derivatives using GPR.

As a simple example, assume that we observe data values **Y** at data locations **X**, but we wish to model the estimator as a sum of values of the elementary estimator function ,
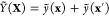
39where **x** and **x**′ are subsets of the degrees of freedom in **X**,
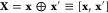
40using a kernel function that is defined between points in the smaller space, *k*(**x**, **x**′). In the spirit of the function-space view of GPR, it follows that the covariance of two such observations *Y*_1_ and *Y*_2_ (taken at **X**_1_ = **x**_1_ ⊕ **x**_1_′ and **X**_2_ = **x**_2_ ⊕ **x**_2_′, respectively) is given by the sum of kernels,
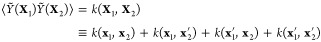
and the rest of the regularized kernel regression formalism follows using this definition of the kernel. When building a sparse GPR model, we have the choice of picking representative points such as **x** from the smaller space or **X** from the larger space. In either case, kernels can be computed between the observed data locations and representative points, e.g. *k*(**X**_1_, **x**) = *k*(**x**_1_, **x**) + *k*(**x**_1_^′^, **x**).

It is straightforward to generalize this construction to *any* linear functional observation, and the resulting kernel model becomes a linear functional of the corresponding kernel functions. To formalize this, we model the observations as
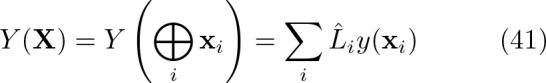
41where *L̂*_*i*_ is a linear operator applied on the elementary model function *y*. In the previous example, *L̂* was simply the identity operator, but it can also include differentiation, scaling, or any other linear operation. To illustrate how fitting based on derivative observations can be performed, we consider the derivative of the estimator function defined in eq 15 with respect to the α component of the input vector, **x**, viz.
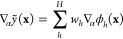
42We obtain the covariance of two such derivative observations as
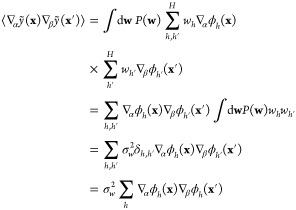
43from which it follows, using eq 19, that the kernel for derivative observations is the double derivative of the original kernel:
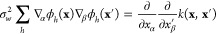
44

**Figure 5:**
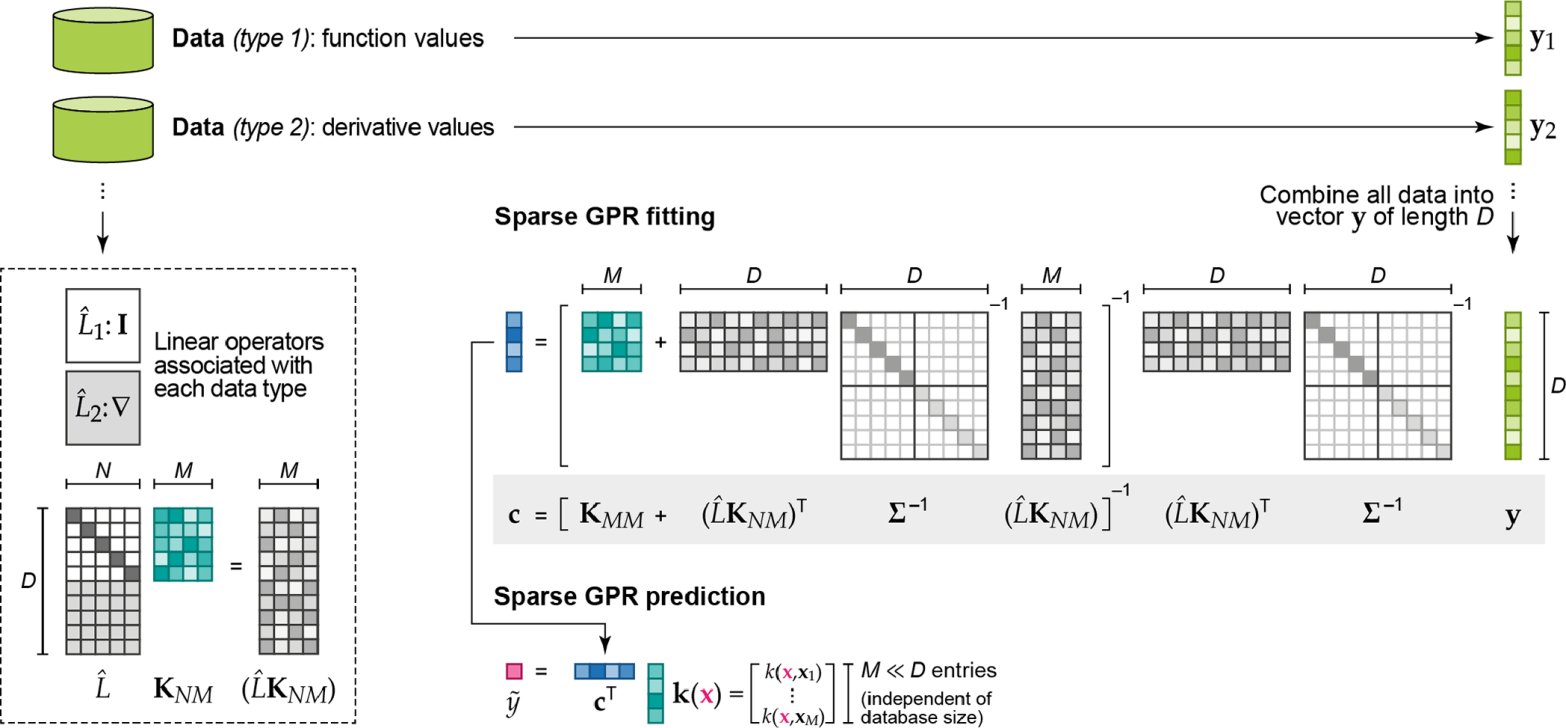
Sparse GPR fitting based on different types of input data (eq 47). In this example, two types of data are present in the reference database: function values (corresponding to the identity operator, **I**) and derivatives (corresponding to the differential operator, ∇). All these observations are combined into a single vector, **y**, which has *D* entries. The fit itself proceeds as shown in Figure 4b but now includes the use of a matrix of operators, *L̂*. Some sizes of vectors and matrices (*M*, *N*, or *D*) are indicated. The regularization, **Σ**, is indicated as a block diagonal matrix (one block corresponding to function values, one to derivatives); more individual settings are also possible. Once **c** is determined, it is used for sparse GPR prediction in the same way as shown in Figure 4.

In a similar manner, the covariance between a function value and a derivative observation can be found as
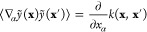
45allowing a covariance matrix to be built for arbitrary observations that are linear functionals of an underlying function. For example, the block of the covariance matrix corresponding to the data vector [*y*, ∇_1_*y*], collected at the points [**x**, **x**′], is given by
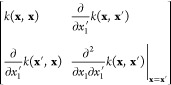
46For a general linear operator, *L̂*, the coefficients in eq 11 that constitute the regularized solution of the regression problem then become
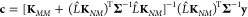
47where **y**, of length *D*, contains all the training data. This matrix equation is visualized in Figure 5. When implementing this in code, the operator  is applied to the kernel matrix **K**_*NM*_ which results in a matrix of size *M* × *D*, that we label  or alternatively 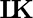.

Figure 3 illustrates these concepts for a simple one-dimensional function (dashed lines) for which GPR estimates are made (solid lines). The examples presented here show “full GPR” fits (i.e., when the set of representative points associated with the basis functions is exactly the same as the set of input data points) in the two cases when either function values (Figure 3a) or derivative values (Figure 3b) are used in the regression. In each case, we show two choices for the length scale of the squared exponential kernel, σ_length_, namely a value that is too small, and also a larger (near optimal) value. If the length scale is chosen too small (left panels in Figure 3), the result is a terrible overfit in both cases, but showing different behavior. When fitting to function observations, the fit matches the data exactly near the data points (red points) and reverts to near zero away from the data points. When fitting to derivatives, the estimate has the correct derivatives locally but overall is nearly zero everywhere. For the near optimal value of the length scale (right panels in Figure 3), fitting to function observations results in an excellent fit near the right-hand side minimum where there are a lot of data and a rather poor fit elsewhere. Fitting to derivatives reproduces the shape of both minima, and the maximum in between qualitatively too, despite there being no data points there. However, the relative depths of the two minima are not well captured.

**Figure 6:**
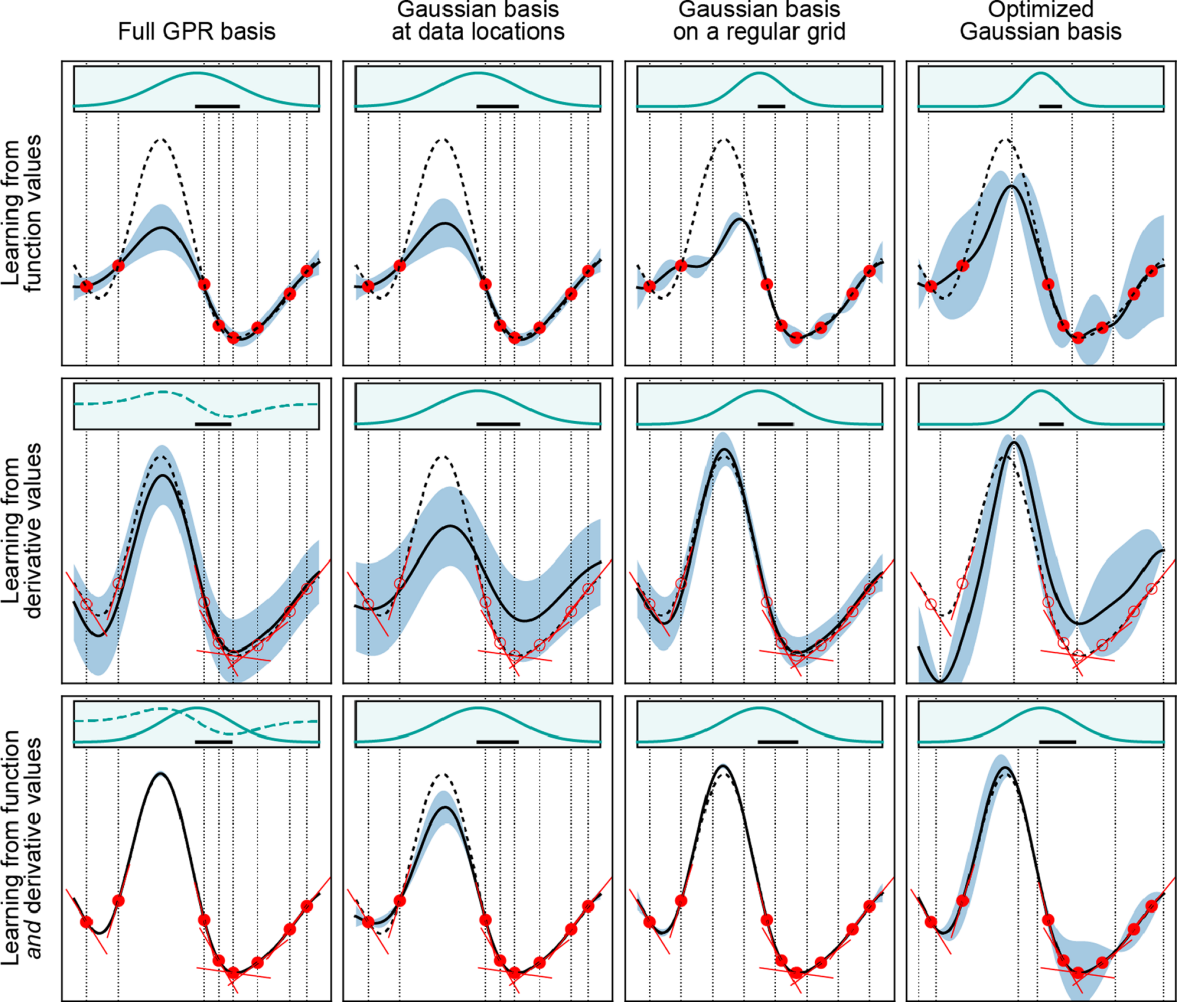
Effects of different types of data and basis functions on GPR fits. These are illustrated using the same example function as in Figure 2 (black dashed lines), showing the predicted mean (black solid lines) and variance (light blue shaded area) of the fit. Observations are indicated by the red points for values and short red line segments for derivatives. The fitting data included only function values in the first row, only derivative values in the second row, and both function and derivative values in the bottom row. Full GPR was used for the data shown in the first column, and sparse GPR for those in the others. Representative point locations (vertical dotted lines) coincide with the data point locations for the first and second columns, whereas they were placed at regular intervals for the third column. In the fourth column, the number and location of representative points were optimized to maximize the marginal likelihood. The regularization hyperparameter σ as well as the length-scale hyperparameter σ_length_ were independently optimized for each panel to maximize the marginal likelihood. Insets show the kernel basis functions used in the fit (solid for Gaussians; dashed for Gaussian derivatives); scale bars represent the optimized values of σ_length_.

In Figure 6, we show the fit quality for the same simple one-dimensional function, but this time using sparse GPR and exploring different ways of constructing the representative set and the corresponding basis set. In the first row, only function values are used in the fit, in the second row, only derivatives are used, and in the third row, function values and derivatives are combined to form the dataset. The first column shows full GPR (as in Figure 2), using square kernel matrices and placing a basis function on each data point, with the basis function type corresponding to the data type: function value observations induce Gaussian basis functions, and derivative value observations induce Gaussian-derivative basis functions (cf. eq 45). Therefore, in the first column of Figure 6, the top panel shows a fit to the function values and uses eight Gaussian basis functions, the middle panel shows a fit to only derivative values and uses eight Gaussian-derivatives, and the bottom panel shows a fit to all the available data and uses both types of basis functions (16 altogether). The improvement in the fit from top to bottom is steep, with the bottom panel showing an almost perfect fit.

The second, third, and fourth columns of Figure 6 all use sparse GPR fits, but with Gaussian basis functions irrespective of what the type of the data is (that is, even if only derivative values are used). In the second column, the eight basis functions are simply placed at the input data locations (and thus the first two panels of the first row are identical!). In the third column, again we use eight Gaussian basis functions but they are centered on a regular grid. This has little effect when the fitting data consist of function values, but it shows a considerable improvement in rows two and three, when derivative data are used. In the fourth column, the locations of the representative set are optimized (by maximizing the marginal likelihood; see below). Note that fewer than eight basis functions are used, because some of the basis function centers have merged during the optimization. We observe some improvement in the first row and an improved estimation of the maximum in the second row, albeit with a poor description of the relative depths of the minima. In the last row, when both the function value and derivative information are provided, the fit is as good as using a regular grid and almost as good as with full GPR (first column).

Studying such simple toy models can be very instructive in understanding GPR, but of course one has to be careful in drawing conclusions and applying them to the high-dimensional problems of materials and molecular data. Nevertheless, it is clear that full GPR does not scale to large datasets and that high dimensionality precludes the use of regular grids when setting up basis sets—indeed the fundamental reason why GPR is efficient even in many dimensions is because the basis set can *adapt* to the data locations. In the Gaussian approximation potential (GAP) scheme, detailed in section 4, the construction is most similar to column-four-row-three of Figure 6, because both total energy and derivative data are used, and the representative set is selected as the optimized subset of the very large number of atomic environments that are present in the input dataset.

The general formulation in eq 41 for modeling arbitrary linear observations in the framework of sparse GPR allows for the complete separation of the basis functions of the representative set and the training data. This greatly simplifies the application of GPR for force-field development, where a large proportion of the training data are in the form of atomic forces. This is because each structure contributes three times the number of atoms as Cartesian force components and just one total energy value. Attempting to use full GPR would result in square kernel matrices with row and column sizes equal to the number of input data values, which in turn would limit the models to rather small datasets. Therefore, in the context of interatomic potentials, where the fitting data correspond to total energies (sums of many atomic energies), forces, and stresses (sums of partial derivatives of many atomic energies), we model the atomic energy as the elementary function and use representative points that are individual atomic environments and corresponding to kernel (rather than kernel derivative) basis functions.

### 2.5 Regularization

Regularization can be regarded as a mechanism to deal with noisy and incomplete data, which balances the requirements of a smooth estimator and a close fit to the data. We introduced the Tikhonov regularization term when we described the weight-space view of GPR in section 2.1 and made the connection with the noise model assumed for function observations in section 2.2 in the function-space view of GPR. From a Bayesian point of view, a noise parameter that is significantly larger than the covariance of function values, σ^2^ ≫ *k*(**x**, **x**′), favors the prior assumption on the function space, which is smoothness, and ultimately leads to the trivial solution of the constant function *y*(**x**) = 0 as σ → *∞* (assuming that the mean of the GP prior is zero). Equivalently, the loss function in eq 4 is dominated by the regularization term for the choice of large σ and leads to the trivial solution of **c** = **0** in the σ → *∞* limit. Conversely, small σ values force the estimator to follow the data points as closely as possible, at the price of potentially significant overfitting. The extreme case of σ = 0 reduces eq 4 to the unregularized least-squares fit.

Apart from these considerations, regularization is of practical relevance from the point of view of numerical stability: it conditions the kernel matrix by adding a diagonal matrix with positive values. In the case of the location of some data points coinciding, the determinant of the kernel matrix **K**_*NN*_ would otherwise become zero, and the inverse **K**_*NN*_^–1^ would become undefined without conditioning the diagonal values. Of course, it would be possible to remove exactly duplicate data points, but even close data points would cause numerical instabilities in practice, which are less trivial to eliminate. Furthermore, it may actually be desirable to have multiple data points at the same or similar locations if the observations do genuinely contain noise, as the observed function values would sample the function, and GPR would effectively and automatically perform averaging. Note that noise in the observations does not have to be of stochastic nature: even in the case of deterministic observations, model error can give rise to deviations that appear as noise, as we will discuss in section 4.

### 2.6 Hyperparameters

A particularly appealing feature of GPR is that it is parameter-free, in the sense that once the prior assumptions (i.e., the kernel and the observation noise) are specified, the function estimator follows. In some cases (particularly when working in the well-established field of atomic-scale modeling), the appropriate kernel and the observation noise might be known. For example, we might have a very good idea of how smoothly the atomic forces vary with atomic position (corresponding to the length scale hyperparameter, σ_length_, introduced above), or how much error we expect in observed values (corresponding to σ) due to a lack of convergence in the electronic-structure computations that provide the fitting data. But often, the hyperparameters describing the problem are not available. In section 6 below, we will describe strategies to set these for material models that we found effective. Formally, when using sparse GPR, the locations of the basis functions are also hyperparameters, and their choice can dramatically influence the accuracy of the fit (cf. Figure 6).

In the Bayesian interpretation of GPR, we have already made use of the marginal likelihood^44^ (or evidence; eq 21), which can also be understood as a conditional probability over the hyperparameters,
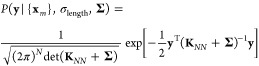
48This provides a route to eliminating all of the unknown hyperparameters, because Bayes’ formula allows one to integrate the likelihood over all possible hyperparameter values when making a prediction. This is essentially an encapsulation of the Bayesian principle of “Occam’s razor”: we are not just interested in hyperparameter settings that lead to small fitting error, but in solutions that are also robust, in the sense that parameters in a large volume of parameter space near the optimum all lead to small fitting error. This turns out to be a good predictor of performance on any future test set, without having to explicitly do the test.

However, integrating the likelihood is often not a practical proposition for large models, because the integral cannot be evaluated analytically. Instead, the hyperparameters corresponding to the highest marginal likelihood are often selected, and these can be obtained in a straightforward way by maximizing the logarithm of the marginal likelihood,
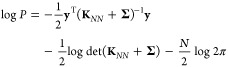
49for which the derivatives with respect to hyperparameters may also be computed.

Another route for hyperparameter optimization, more often used in the context of KRR, is cross-validation. There are many variations to this approach, but commonly the available data are divided into a “training” and a “test” set. The training set is used for the regression, with the predictions evaluated on the test set. The hyperparameters are then adjusted to achieve the lowest possible error on the test set. There are more sophisticated versions, where multiple splits are created (so-called “*k*-fold cross-validation”).

## 3 Learning Atomistic Properties

Let us now show how the general GPR framework translates into a scheme to model the atomic-scale properties of molecules and materials. First, we discuss how the Cartesian coordinates and the atomic numbers that determine the specific configuration of the system should be represented to obtain a description that is suitable for atomistic ML. This is one of the central problems in the field, and we refer the reader to a dedicated review^51^ in the present thematic issue for a more detailed discussion. Here, we limit ourselves to a family of approaches which covers most of the example applications that are discussed in what follows. We then present a “hands-on” example: the construction of a GPR model of the energy and dipole moment of an isolated water molecule. We use this example to introduce the relevant concepts and show them “in action”; for more details, the reader is referred to subsequent sections. We provide Python (Jupyter) notebooks that reproduce the results shown in the present section, and we report code snippets to show the connection between general expressions and the practical implementation for an atomistic problem.

### 3.1 Representing Atomic Structures

The chemical structure of molecules and materials is defined most directly by the Cartesian positions, {**r**_*i*_}, of the constituent atoms. Interatomic potential models do not typically use these positions as input directly but rather transform them into a different mathematical *representation*. This way, the resulting potential can automatically gain some desirable properties, particularly symmetries of the potential energy with respect to translation, rotation, and permutation (swapping) of atoms of the same element. Furthermore, the representation should reflect other physical requirements, such as smoothness of the mapping, additivity when applied to the learning of extensive properties, as well as correct limiting behaviors, e.g., that the atomization energy is zero (by definition) when atoms are at infinite separation.

**Figure 7:**
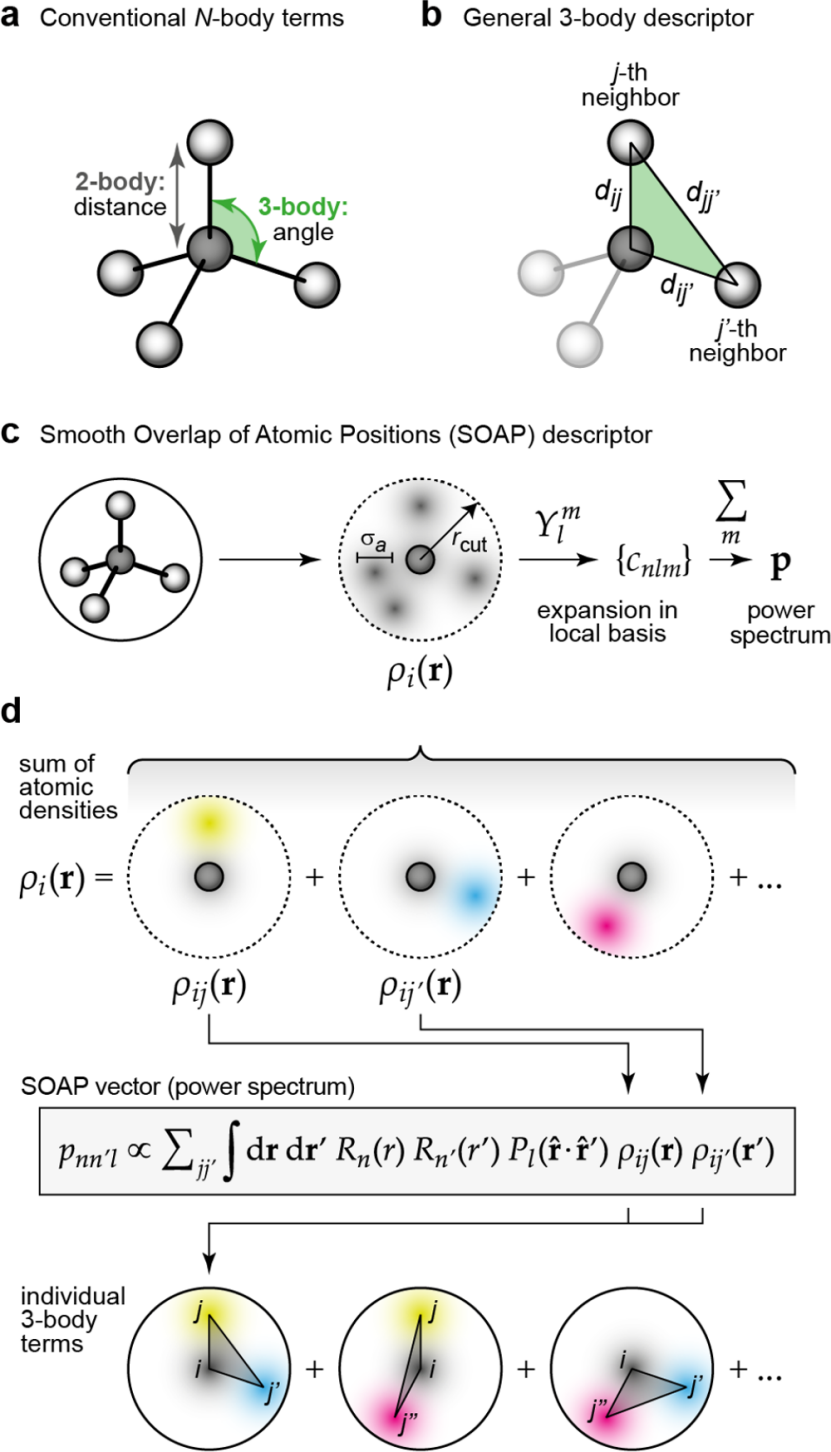
Descriptors for atomistic structure. (a) Conventional 2-body and 3-body terms, viz. distances and angles between atoms, as typically used in empirical force fields. Adapted from ref (29) with permission. Copyright 2019 Wiley-VCH. (b) General descriptor for 3-body terms: the three distances, *d*, between the atoms, specify the relative geometry of the three atoms completely. (c) Schematic of the smooth overlap of atomic positions (SOAP) descriptor.^52^ The neighbor density ρ is permutationally invariant; expanding it in a local basis of radial functions and spherical harmonics, *Y*_*l*_^*m*^, and then summing up the square modulus of the expansion coefficients *c*_*nlm*_ over the index *m* ensures rotational invariance of the power spectrum **p** (eq 56). Adapted from ref (53). Original figure published under the CC BY 4.0 license (https://creativecommons.org/licenses/by/4.0/). (d) Illustration why the power-spectrum vector, **p**, is a 3-body descriptor (here shown without element indices for clarity); the consequences of this are discussed in the text.

The classic example of such a transformation is to represent the relative positions of two atoms *i* and *j* by their mutual distance (Figure 7a),
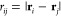
50If, in addition, the potential energy is written as a separable sum of functions of these distances, the result is a *pair potential*,
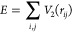
51where *V*_2_ is a one-dimensional function. The simplicity of the above form obscures its implications as the basis of a regression model for atomic-scale properties. The fact that the interatomic distances are independent of an absolute coordinate reference frame guarantees that the potential is invariant with respect to translation and rotation. Since the true potential energy obeys these invariances exactly, this is universally agreed to be a good thing. The true potential is also invariant to permutation of like atoms, and the separable form and the sum over each pair of atoms guarantees this invariance but at the cost of a drastic approximation: the true quantum-mechanical energy is *not* separable into a sum of pairwise terms. Whether this approximation still results in a usable potential depends on the system: the Lennard–Jones pair potential is an excellent approximation for noble gases, and similar models give qualitatively decent models of simple fcc metals^54^ and simple ionic halides^55^ and some oxides.^56^ For covalently bonded systems, pair potentials that reflect the connectivity of the system can provide reasonably accurate descriptions of small displacements, e.g., vibrational dynamics, but fail to give a natural description of chemical reactivity and are basically unusable as general-purpose models.

The traditional route to improving the potential is to add a correction in the same spirit, but at a higher body order, i.e. a term that explicitly depends on the positions of three atoms (Figure 7b) and is summed up over all atom triplets in the system to preserve permutational symmetry:
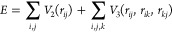
52The three-body term is sometimes approximated to explicitly depend only on the angle between the three atoms (cf. Figure 7a), rather than their individual distances, thereby reducing the number of adjustable parameters. Interestingly, as a result of recent developments in high-dimensional fitting using many parameters, it has become apparent that a lot can be accomplished with just three-body but fully flexible potentials.^57−62^ In principle, one could continue along this direction and add even higher-order, viz. general four-body, terms. Because of the complexity of managing the increasing number of parameters while still maintaining the permutation symmetry exactly (which involves summing over all atom tuples of increasing size), this has only been done systematically for small molecules^63^ and is only now beginning to be explored for larger systems^64^ and materials.^65^

An alternative approach is to not make the approximation of separability in body order in the first place but instead to write the total energy of the system as a sum of atomic (“local” or “site”) energies that depend on many-body descriptions of atomic environments. This, however, requires a representation that itself is invariant to permutation of like atoms and also incorporates the approximation that interactions are of finite range. The foundational works of Behler and Parrinello^66^ and Bartók et al.^49^ precisely hinged on such innovations: the former, on atom-centered symmetry functions; the latter, on spherical harmonic spectra, originally the bispectrum and later the power spectrum,^52^ also called the smooth overlap of atomic positions (SOAP; Figure 7c). Coupled with nonlinear regression models, the remaining significant approximations are controlled by the number of training data points and the interaction range. All of our examples in sections 6 and 7 will use the SOAP representation, and so we give a brief definition here for completeness.

To obtain the SOAP representation of the neighborhood of a given atom *i*, we first build a set of neighbor densities, one for each chemical element in the set that is relevant for the system at hand:^52^
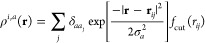
53where the sum is over neighbors *j* of element *a* that are within the cutoff *r*_cut_ and *f*_cut_(*r*) is a cutoff function that smoothly goes to zero at *r*_cut_. The hyperparameter *σ*_*a*_ has units of length and determines the regularity (smoothness) of the representation. The above neighbor density is thus a *mollified* version of a neighbor distribution where each atom would be represented by a Dirac delta function. It is tempting to associate the Gaussian mollifier with an atomic electron density or a smeared nuclear charge, but the correspondence is not so direct. The direct effect of the mollification in the density is only to ensure that the interatomic potential constructed using the SOAP representation is regular, and it would be reasonable to construct a SOAP representation from Dirac delta densities, given that the regularity of the potential is ensured in some other way. For example, the moment tensor potentials (MTP)^67^ and the atomic cluster expansion (ACE)^68^ do exactly that.

In the following, for each expression, we will give both the notation that was introduced in ref (52) and (highlighted in blue) a recently proposed^69^ bra-ket notation of the form 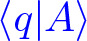 that uses *q* to describe the indices enumerating the entries of a feature vector and *A* to indicate the nature of the representation. Note that the expressions typeset in blue are here to make the connection to ref (51) explicit and are not needed to follow most of the exposition in the present review. Using this notation, for example, the equivalent expression corresponding to eq 53 reads:
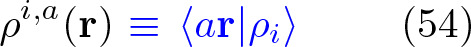
54It is important to emphasize that for each atom *i*, irrespective of what element it is, the full set of elemental neighbor densities is constructed. Each elemental neighbor density is invariant to permutations of that element. To achieve rotational invariance, we first expand the neighbor density in a basis of orthogonal radial functions, 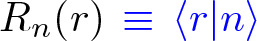, and spherical harmonics, 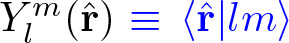,
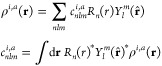
55or equivalently,
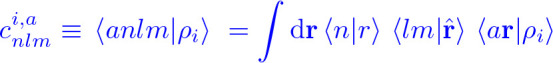
where the expansion coefficients are labeled *c*_*nlm*_^*i,a*^ for consistency with earlier publications^34,52^ and are not to be confused with the coefficients of the kernel regression model that have been introduced in section 2. Note the similarity with how atom-centered orbitals, containing radial and angular parts, are constructed in quantum chemistry. As emphasized by the bra-ket notation, the expansion in spherical harmonics just amounts to a change of basis, and these coefficients are *not* rotationally invariant. A symmetrized combination of these coefficients yields the power spectrum,
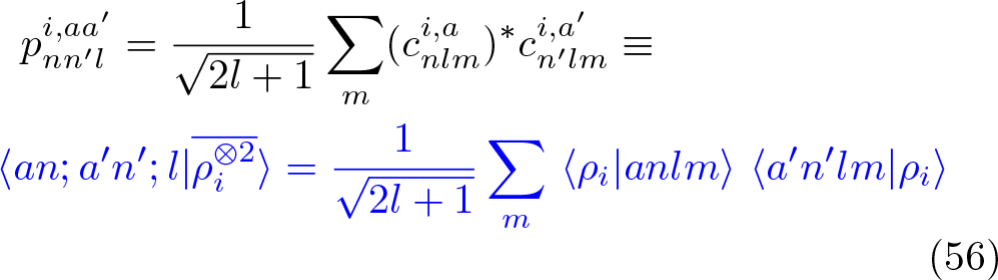
56where the notation  hints at the fact that the SOAP power spectrum is obtained by averaging a two-point tensor product of the atom density over rotations—which, in the spherical harmonic basis, is equivalent to summing over *m*. The *l*-dependent prefactor in the definition of the power spectrum is necessary to make a connection to the overlap of densities (see below). Note that various other constant numerical factors have appeared in the definition in the past,^34,52^ but none of them are consequential, because the power spectrum is typically normalized to yield a unit length vector. The descriptor for each atomic environment now has five indices: two for the neighbor-element channels (*a*, *a*′), two radial channels (*n*, *n*′), and an angular channel (*l*). This power spectrum, also commonly referred to as the SOAP descriptor, or SOAP vector, is a concise representation of atomic neighbor environments. It is smooth and continuous with respect to atomic displacements and invariant with respect to physical symmetries, and its only free parameters, the cutoff and the length scale, *σ*_*a*_, are physically intuitive.

An important question in the context of building atomistic regression models based on any structural descriptor is whether the descriptor is *complete*, in the sense that two atomic environments that are not related by symmetry should map to different descriptors (that is, whether the functional definition of the descriptor is injective). If this were not the case, the accuracy of *any* ML model based on the descriptor would be ultimately limited by the corresponding loss of information. Since their introduction, it was believed or implied^70^ that SOAP and all related descriptors (i.e., those that are based on three-body correlations, such as the atom centered symmetry functions of Behler and Parrinello^66^) are complete. Recently, however, it was discovered that neither SOAP nor the other equivalent descriptors are complete, and counter-examples were shown also for the higher-order bispectrum (which corresponds to four-body correlations).^71^ Therefore, SOAP-based models cannot describe an atomic energy function of its neighborhood to arbitrary precision, although practical successes suggest that the corresponding errors are on the same order or smaller than other systematic errors that are due to locality and *k*-point sampling (see below for a more detailed discussion of these). Yet, it may well be possible that complete descriptors can lead to more efficient learning; see ref (51) for more details.

The full SOAP descriptor for each atom *i* contains all entries of *p*_*nn*^′^*l*_^*i,aa*^′^^, resulting in a vector whose length scales with the square of the number of elements (due to the presence of the two element indices, *a* and *a*′), with the square of the radial basis expansion limit (due to the two indices *n* and *n*′), and linearly with the angular basis expansion limit (due to the index *l*). This vector has hundreds of components (thousands, for systems with several elements) when the basis expansion of the neighbor density is truncated in *n* and *l* such that these truncations do not give rise to noticeable inaccuracy. It is therefore natural to think about suitable *subsets* of the SOAP vector components that could be used without compromising accuracy. There is a highly abstract question here: given the dimensionality of the Cartesian positions, most of the SOAP components must be algebraically related to one another. Knowing such relationships would be useful in reducing the number of components to the independent ones, although it is quite likely that a regression model might work significantly better with more inputs, even if many of those are not independent, because the functional relationship being modeled might be simpler. We are not aware of any theoretical results in this area. On the practical side, however, given datasets and specific regression models, one can numerically experiment with choosing subsets of the SOAP components, and considerable compression is possible.^72−74^

While such atomic environment descriptors can be used as the basis of any kind of regression scheme, to use them in GPR (which is the focus of the present review), we need to define a kernel that allows us to compare two atomic environments, denoted *A* and *A*′. While a standard Gaussian kernel is certainly an option, applications to date have used low-order polynomial kernels, viz.
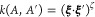
57where **ξ** and **ξ**′ indicate the feature vectors corresponding to the normalized power spectrum vectors, **ξ** = **p**/|**p**|, associated with the two environments—with the power spectrum vector associated with an atom *i* being built from the components that are defined in eq 56, viz.
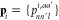
58Considering the linear kernel (ζ = 1) explains the origin of the SOAP name (cf. “smooth overlap of atomic positions”), because the dot product of the power spectra is equivalent to the rotationally integrated squared overlap of the corresponding neighbor densities of two atoms,^52^
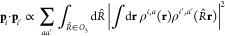
59where *R̂* is a 3D rotation. A kernel model made using this linear kernel results in a three-body model, i.e. one in which the model can be written as a sum, over triplets of atoms, of a function which only depends on the Cartesian coordinates of the triplet.^58^ This is not obvious, but it follows from the fact that the SOAP vector itself is a three-body representation of the atomic environment, which is not obvious either, but which we show as follows (and have illustrated in Figure 7d).

Let us separate out the contribution of each neighbor *j* to the neighborhood density of atom *i*,
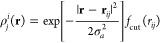
60so that the neighbor density for element *a* is simply
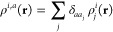
61We then form the two-point correlation of this density,
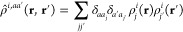
62and compute the SOAP vector by transforming it into the spherical harmonic basis in both arguments and then summing over *m* to ensure rotational invariance:
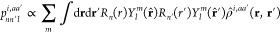
63
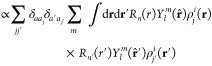
64
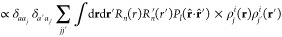
65where *P*_*l*_ are the Legendre polynomials. Thus, the SOAP vector elements can be written explicitly as sums over pairs of neighbors. In all software implementations, eq 56 is used to compute the SOAP vectors, because that makes the calculation independent of the number of neighbors—this is commonly referred to as the “density trick” and is essentially the swapping of the sum and the integral in the last expression.

Using ζ = 2, i.e. raising the scalar product to the power of 2, results in dependence on four neighbors and together with the central atom yields 5-body terms; in general, the body order of the model is 2ζ + 1. Quantum mechanics is a fundamentally many-body theory, and although it is clear that for many properties an expansion in atomic body order is a good idea, formally all body orders are necessary for convergence. In the kernel framework, there is no extra computational cost to increasing the body order in this way, because there is no explicit sum over atom tuples: the SOAP components are computed just once, and the body order is set when the kernel is evaluated between environments. Yet, it is likely a good idea to not choose the body order higher than necessary for achieving the target accuracy: a model with lower body order, and therefore with lower dimensionality, will converge more quickly to its ultimate accuracy as the amount of input data is increased. Many successful SOAP-GAP interatomic potentials for materials have been built with ζ = 2 and ζ = 4, and such potentials and their applications are discussed in section 6.

We note that this link between the body order of the model and the quadratic nature of the power spectrum features (and the fact that the bispectrum features correspond to the next body order) leads naturally to *body ordered linear models*, that are three-body potentials if they use the power spectrum^58,75−77^ and four-body when using the bispectrum (because its terms are cubic in the neighbor density coefficients), as is the case for the spectral neighbor analysis potential (SNAP).^78^ One can go further in body order explicitly while continuing to keep the regression linear.^64,65,67,68,73,79^

### 3.2 Symmetry-Adapted Representation

In contrast to scalar properties such as the potential energy, which are invariant under rotations of a system, tensorial properties such as molecular dipole moments and material polarizations transform covariantly when the system is rotated. A natural way to account for this covariance is to build it into the training and prediction processes. The procedure for doing so was first discussed by Glielmo et al. in the context of learning Cartesian vectors.^80^ They noted that the GPR interpretation of a kernel function as a covariance naturally dictates the symmetry properties of kernels for predicting vectors, requiring the kernel function *k*(**ξ**_*i*_, **ξ**_*i*′_) to be replaced by a matrix-valued function **k**(**ξ**_*i*_, **ξ**_*i*′_). In this function, the block *k*_*αα*__′_(**ξ**_*i*_, **ξ**_*i*′_) represents the coupling between the Cartesian component α of a coordinate system centered on the *i*-th atom and the coordinate α′ of a reference system centered on the *i*′-th atom. A number of symmetry-adapted methods for predicting tensors have appeared in recent years, generally relying on the use of reference frames based on the internal molecular coordinates. These have been successfully applied to generate ML models for the multipole moments of small organic molecules^81,82^ and the hyperpolarizability of water,^83^ as well as being used to predict vibrational spectra, including infrared spectra of organic molecules^84,85^ and the Raman spectrum of liquid water.^86^ It has become clear in the past few years that both linear^73,79,87^ and fully nonlinear^88−90^ models can be built using covariant representations.

It is possible to generalize the approach of ref (80) to arbitrary orders of tensor by applying analogous symmetry arguments,^91,92^ and we refer to the resulting method as symmetry-adapted GPR (SA-GPR). Rather than working with Cartesian tensors, it is more convenient to decompose them into their irreducible spherical components,^93^ which are more naturally related to the transformation properties of the rotation group and afford a more concise description of the problem. For instance, the polarizability (a symmetric 3 × 3 tensor with six independent components) can be decomposed into its trace, which transforms as a scalar, and a 5-vector that transforms as a λ = 2 spherical harmonic. (Note that we use λ to indicate the angular momentum symmetry of the fitting target, rather than *l*, to distinguish it from the analogous angular momentum index that appears in the density expansion.) Given that a covariant kernel must describe the correlations between the entries of the tensors associated with two environments, this transformation allows us to work with a 1 × 1 and a 5 × 5 kernel, rather than one with 6 × 6 entries. The transformation between Cartesian and spherical tensors is not entirely trivial for λ > 1, but it is well-established^93^ and necessary for separating the Cartesian tensor into components according to how they transform under rotation. The basic form of a kernel that is suitable for fitting spherical tensors of order λ is a generalization of the SOAP kernel of eq 59:
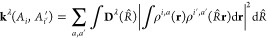
66 is the Wigner *D* matrix of order λ. These kernel matrices encode information on the relative orientation of the two environments, as well as their similarity, and are referred to as λ-SOAP kernels. A kernel built using eq 66 satisfies the two properties that are necessary for learning a tensorial quantity: namely, that the predictions of a SA-GPR model are invariant to a rotation of any member of the training set and that when a rotation is applied to a test structure, the predictions of the model transform covariantly with this rotation.

For λ = 0, which has eq 66 reduces to the expression for the scalar SOAP kernel. For the general spherical case, the integral of eq 66 can be carried out analytically.^91^ In practice, the kernel can be computed from an equivariant generalization of the power spectrum,
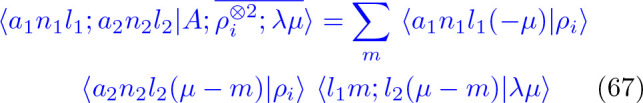
67where  is a Clebsch–Gordan coefficient, 
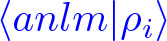
 is a density expansion coefficient (eq 55), and the notation 
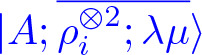
 alludes to the fact that these are features obtained from the symmetrized average of a two-point density correlation (akin to SOAP) that transforms under rotation as a spherical harmonic *Y*_*λ*_^μ^. The λ-SOAP kernel (eq 66) can be obtained by summing over the feature indices,
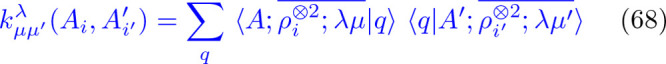
68where we use  as a shorthand notation for the full set of indices .

### 3.3 H_2_O Potential Energy: A Hands-On Example

#### The Dataset

**Figure 8:**
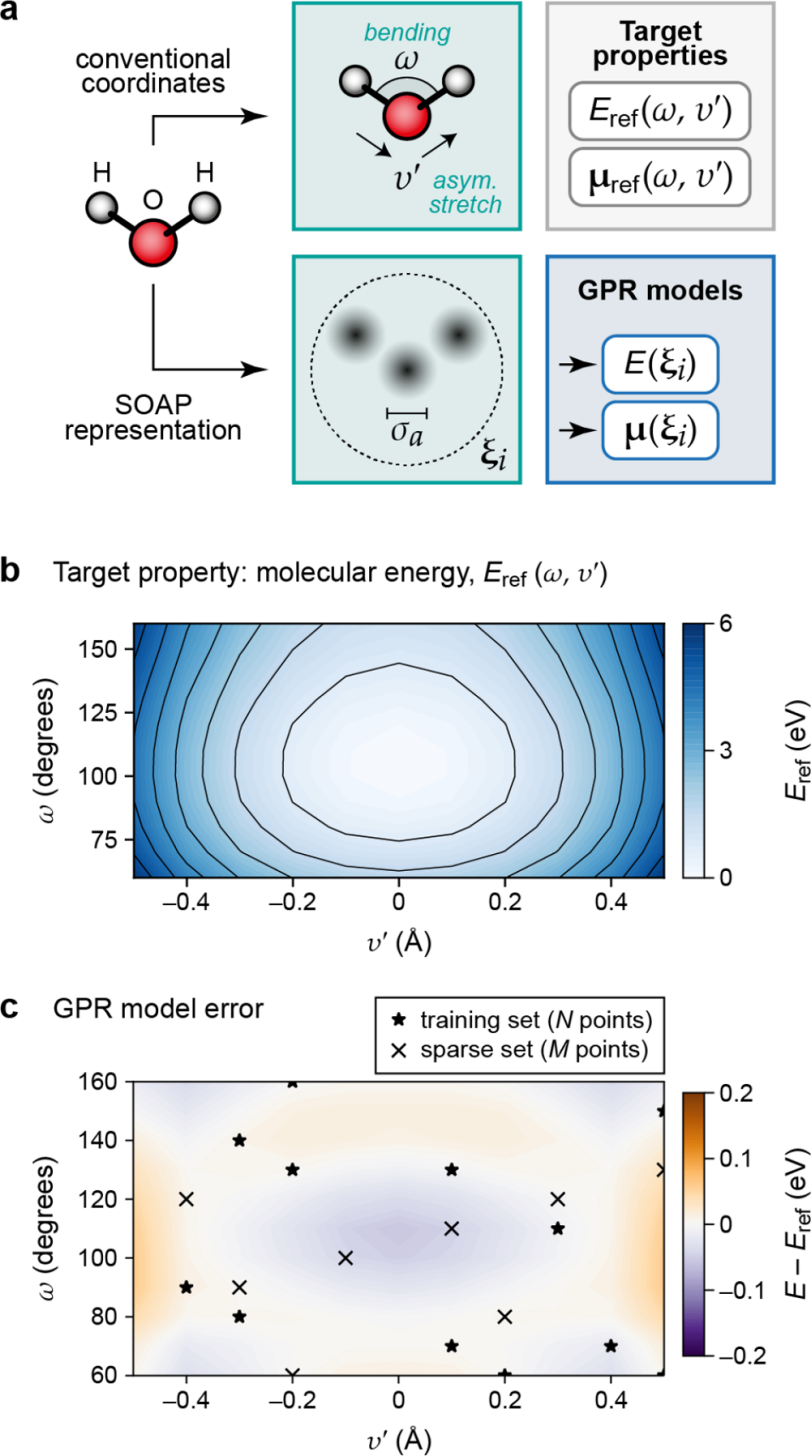
Hands-on example for atomistic GPR: learning the potential-energy surface of a single water molecule. (a) Structures in the dataset are defined by two coordinates: the asymmetric stretch coordinate ν′ and the bending coordinate ω; the sum of both bond lengths is fixed to 2 × 0.95 Å. (b) Target property to be represented by the model, spanning several eV because a very large range of distortions has been chosen for this toy example. (c) Error in the GPR-predicted molecular energy as a function of (ν′, ω). Stars indicate structures used for training; crosses indicate structures used as representative points of the sparse GPR model.

We consider as a toy model the prediction of the energy of a water molecule, deformed along the bending coordinate ω and the asymmetric stretch coordinate ν′ = *d*_OH^(1)^_ – *d*_OH^(2)^_, with fixed symmetric stretch coordinate  Å (Figure 8). The dataset is a collection of 121 configurations, equally spaced along the two directions in an 11 × 11 grid, which we use below to select training and representative set configurations. For each configuration we evaluate the energy, *E*, and the dipole moment, **μ**, using the Partridge–Schwenke model,^94^ which constitute the targets for regression. These structures are highly distorted, with energies in the electronvolt range relative to the most stable configuration. Note that we choose one of the coordinates to be the asymmetric stretching coordinate, ν′, so that the manifold is symmetric with respect to reflection relative to ν′ = 0, corresponding to a swap of the labels of the two hydrogen atoms.

#### Computing Features

The structure of the molecule is uniquely determined by just the two O–H distances and the H–O–H angle. However, for the purposes of this example, we parametrize the GPR model in terms of the SOAP power spectrum features *p*_*nn*__′*l*_ (equivalently 
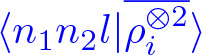
) centered on the O atom with *σ*_*a*_ = 0.5 Å.

Using the SOAP implementation of the librascal package,^95,96^ as illustrated in the Python notebook that is provided to accompany the present paper, we compute the feature matrix using the following code:

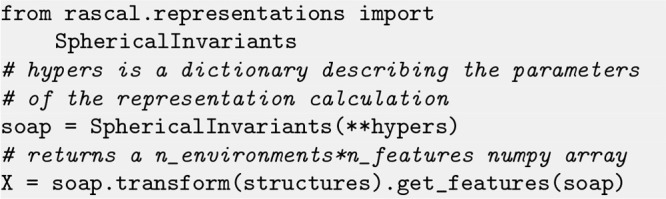



In this code extract, the structures are loaded and stored in the variable structures (in the Atoms format of the Atomic Simulation Environment, ASE,^97^ to which librascal,^95,96^ QUIP,^98^ and some other SOAP implementations are coupled). The hyperparameters (hypers) describe the extent and shape of the cutoff function delimiting the atomic environment, the spread of the atom density, the parameters of the radial and angular expansion, and how the feature vectors should be treated after being calculated. These parameters could be optimized by cross-validation but often can be chosen by hand, taking into account the specifics of the modeling problem.

#### Data Splitting

We split the dataset into a training set (which we indicate with the letter *N* and which is used to determine the model parameters) and test points, which we indicate as *T*, that are used to assess the accuracy of the predictions. This is common practice, as discussed in section 2. The corresponding indices within the overall dataset are stored in the variables itrain and itest. We also select representative points that are used as basis functions to expand the sparse GPR ansatz. We indicate the representative set as *M* and store the indices in the variable irep. It is worth stressing that, even though it is customary to take the representative points to be a subset of the training set, this need not be the case, and methods exist that optimize the feature vectors of the representative points so that they do not even correspond to an actual structure.

#### Kernel Matrices and Regression

As discussed in section 2, the kernel matrix can be built by evaluating a positive-definite kernel function, *k*(**ξ**_*i*_, **ξ**_*j*_), over all pairs of training configurations. The elements of the power spectrum feature vectors for all the structures in the dataset and are collected into a feature matrix **Ξ**, in which each row is associated with one O-centered environment. The linear kernel matrix is obtained as
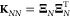
69where each element is a scalar product between the corresponding feature vectors, leading to a model which is equivalent to linear regression. The true advantage of GPR, however, comes when we use the kernel to incorporate an element of nonlinearity into the model. This could take the form of a polynomial kernel (e.g., taking *k*(***ξ***_*i,*_***ξ***_*j*_) = (***ξ***_*i*_·***ξ***_*j*_)^ζ^) or of a Gaussian kernel, *k*(**ξ**_*i*_, **ξ**_*j*_) = exp(−|**ξ**_*i*_ – **ξ**_*j*_|^2^/2θ^2^). The latter allows for the approximation of *any* sufficiently regular function defined on the chosen feature space.^39^ Here we implement a sparse GPR model, which corresponds to the minimization of a loss analogous to eq 7, and so we compute kernel matrices within the representative set (**K**_*MM*_) and between training and representative set (**K**_*NM*_), using a polynomial kernel with exponent ζ = 2, as follows:

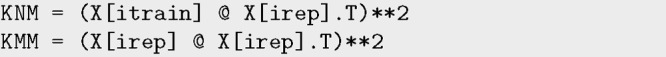



The GPR weights are determined by eq 11 which, using a single value σ for the regularizer, takes the form
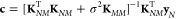
70and can be easily implemented using linear algebra library functions, for example, with a least-squares solver:

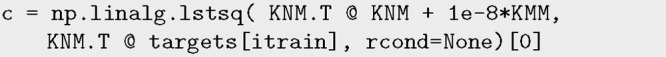



The predictions for the test set, or indeed for any new structure, can be easily computed as **y**_*T*_ = **K**_*TM*_**c**, i.e.

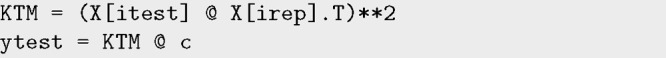



As shown in Figure 8, using only 12 training points and 8 representative points, the model achieves an error below 15 meV, which is less than 2% of the intrinsic spread of energies in the dataset. An important observation is that the errors are exactly symmetric with respect to ν′ = 0: the use of an invariant representation guarantees that molecular symmetries are automatically enforced, which improves the accuracy of predictions even if we do not exploit them explicitly in the selection of the training set.

**Figure 9:**
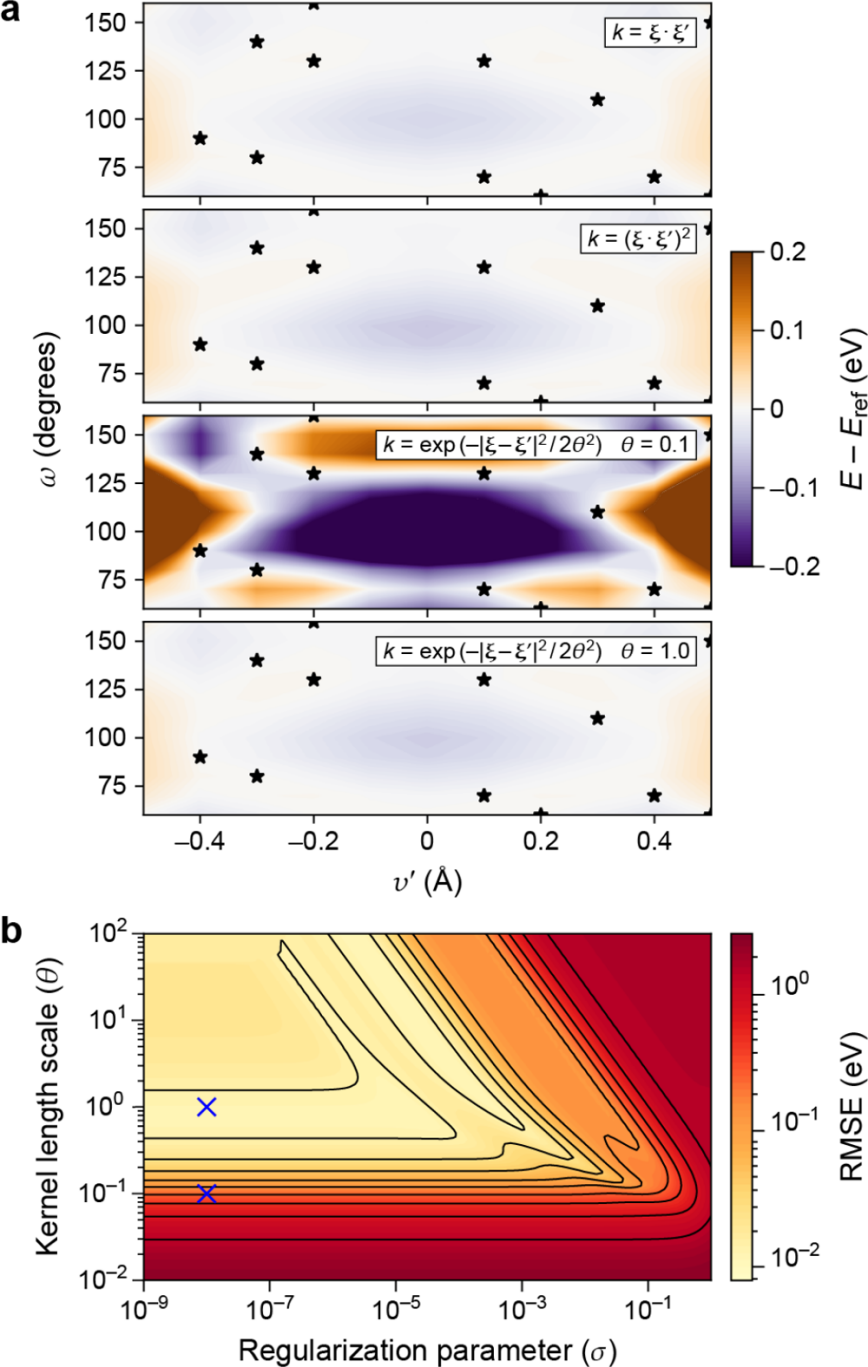
Effect of different kernels and hyperparameters for the H_2_O example. (a) Error in predicting the energy for the distorted H_2_O molecule using different kernels as noted in the legends. Stars denote the training point locations. For all kernels, the features, **ξ**, are the SOAP power spectrum components, centered on the oxygen atom, with *σ*_*a*_ = 0.5 Å and regularization σ^2^ = 10^–8^. (b) Test-set RMSE for the H_2_O energy model based on a Gaussian kernel, as a function of the regularization σ and the kernel length scale θ. The two blue crosses correspond to the hyperparameters used in the lower two graphs in panel a.

Even for this simple problem, the performance of a GPR model depends on the choice of the structure and hyperparameters of the model. The choice of the kernel itself can have a very substantial effect on the accuracy of the predictions and on its ability to fit (and overfit!) the targets. Figure 9a compares the error one incurs when using four different kernels. For simplicity, and to avoid confounding effects, for this figure we use a full (i.e., not sparse) kernel model, even though this is rarely the most effective choice in computational practice. For this simple system, linear regression based on SOAP features has an accuracy comparable to that of the nonlinear, square kernel. The Gaussian kernel, instead, leads to clear overfitting for a small length scale hyperparameter: the training points have zero error, but structures away from the data points in the (ω, ν′) space of Figure 9a exhibit a very large discrepancy (up to 1 eV) between reference values and model predictions. With a larger length scale hyperparameter, the fit accuracy is similar to those with the linear and quadratic kernels.

The effect of changing the hyperparameters for this dataset is shown in Figure 9b. The strongest dependence is on the width parameter of the kernel function used to define the GPR model covariance (here denoted by θ, to distinguish it from a spatial length scale, since the descriptors are the SOAP features). The optimal value is around 1, which is large compared to the typical distance between data points in the space of SOAP features, which is approximately 0.03 for this dataset. In this example, the Gaussian kernel performs well using a hyperparameter for which it is dominated by the first term in the Taylor expansion of the exponential and therefore in effect becomes very close to a linear kernel.

For this simple dataset, which has a low intrinsic dimensionality, the effect of regularization is minor, but one can still see that if the basis functions are wide enough (θ > 1), there is an improvement of the test-set accuracy for finite, nonzero regularization compared to the nonregularized (σ = 0) case. In more realistic scenarios, and particularly in the high-dimensional, data-poor, or extrapolative regime, a careful choice of σ can substantially improve the robustness of a model. Practical aspects of regularization in GAP models are discussed below (section 4.6), as are heuristics for setting other hyperparameters that influence the representation: the SOAP density smearing length scale (*σ*_*a*_), the cutoff radius, and others.

### 3.4 Symmetry-Adapted GPR

We now give an example of the construction of a regression model for a tensorial property, namely, the dipole moment of the water molecules, computed for the same set of distorted structures. Functionally, symmetry-adapted GPR is very similar to standard GPR, with eq 3 for the estimator replaced by
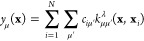
71for the μ component of the spherical tensor **y** (**x**). Given that the dipole moment is just a vector and that (real-valued) *l* = 1 spherical harmonics correspond to (*y*/*r*, *z*/*r*, *x*/*r*), we build the kernel using just the Cartesian components. Hence, the variable yl holds the *N* × 3 components of the dipole moments in the overall dataset.

To compute λ-SOAP kernels, we first compute the corresponding equivariant feature vectors, , implemented in the code as follows:

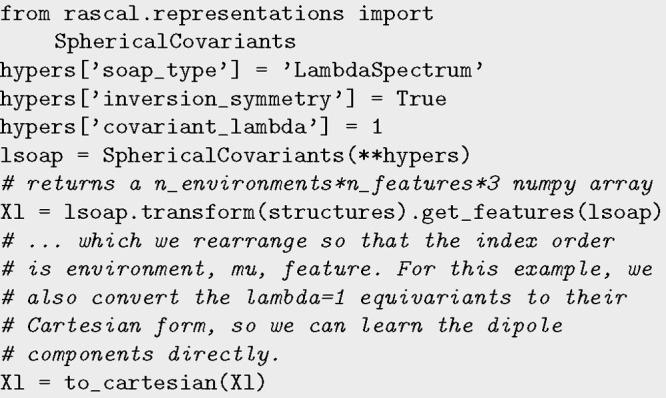



The hyperparameters are the same as for the invariant SOAP, except for covariant_lambda that identifies the required equivariant channel and inversion_symmetry that retains only components with the appropriate behavior with respect to inversion.

Kernels are composed of 3 × 3 blocks, computed using eq 68. This requires some careful indexing:

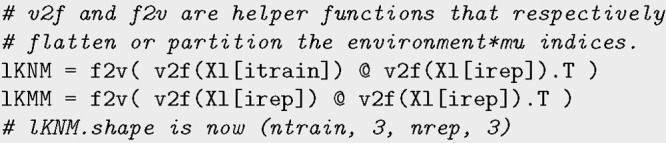



The tensorial expression for the GPR weights is
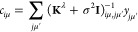
72Hence, in SA-GPR we are solving fundamentally the same problem—that of matrix inversion—as in standard GPR, but we now incorporate the intrinsic geometric correlations between the components of the target properties through the form of the covariance matrix. In terms of implementation, this also requires some bookkeeping:

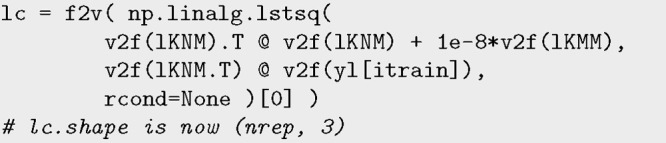



Afterward, it is possible to perform tensorial predictions by just applying eq 71:

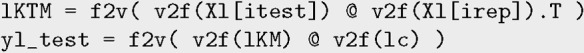



**Figure 10:**
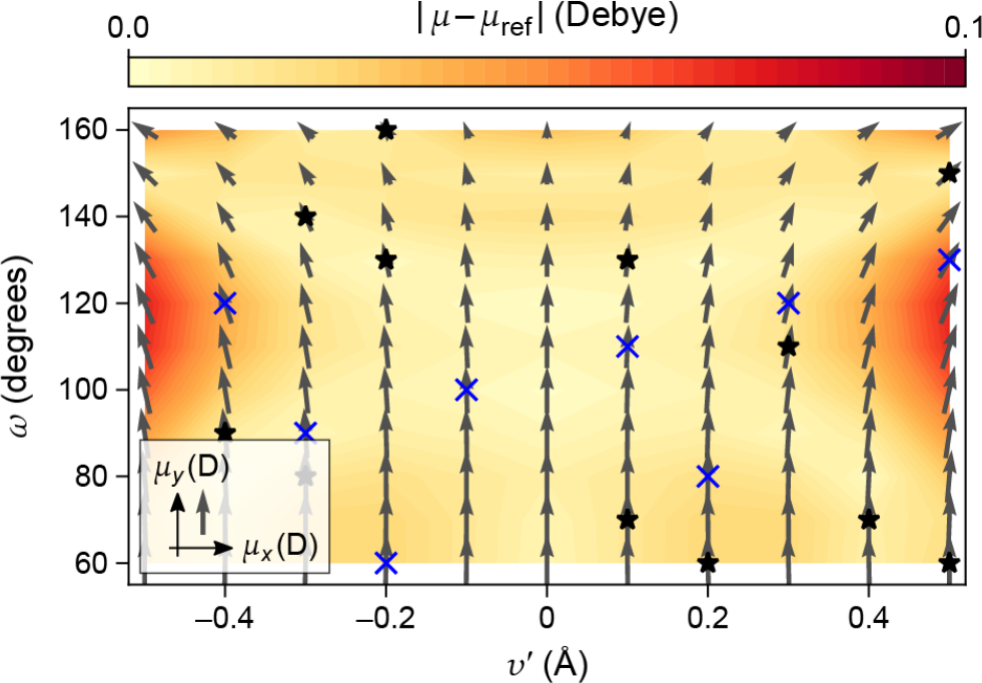
SA-GPR predictions of the dipole moment for the water molecule. The training data were generated using the Partridge–Schwenke model; the definition of the coordinates ν′ and ω, as well as the molecular structures, are the same as in Figure 8. Blue crosses and black stars indicate the representative and training point locations, respectively. Arrows indicate the magnitude and direction of the predicted dipole moment for each structure (the *y* axis corresponds to the *C*_2_ axis of the ideal molecule), and the background color scale indicates the magnitude of the model error. For reference, the typical scale of the dipole moment of a water molecule is 1.8 D (corresponding to the size of the gray arrow in the inset), and so the fitting errors are on the order of a few percent.

Figure 10 demonstrates the accuracy of the SA-GPR model. The predictions are symmetric across ν′ = 0, consistent with the geometry of the problem and a consequence of the equivariant framework. Note that the kernel we use here has a scalar-product form and so is equivalent to a linear ridge regression model built on the  features. As discussed in ref (99), nonlinear tensorial kernels *cannot* be built by manipulating the λ-SOAP block elementwise but should be constructed by combining a nonlinear scalar kernel with a linear tensorial part, e.g.
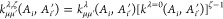
73for a polynomial kernel. (Note that *k*^*λ*^^=0^ corresponds to the original, scalar SOAP kernel defined in eq 57.)

## 4 Gaussian Approximation Potential (GAP) Framework

The introduction of ML methods for modeling the Born–Oppenheimer potential-energy function using suitable descriptors of atomic environments^49,66^ has opened up a new research field in materials science and chemistry. Although there was important early work using ML models (e.g., for the low-dimensional potential-energy surface (PES) of small molecules^100−102^ even near surfaces^103^), the key advance was the systematic description of the many-body environment of atoms, coupled with high-dimensional fitting techniques (neural networks^66^ and kernel methods^49^). The descriptors of Behler and Parrinello and the smooth overlap of atomic positions (SOAP) kernel^52^ obey all physical symmetries (translations, rotations, and permutation of like atoms) and represent the local environment with a high degree of completeness,^71^ while remaining smooth and continuous with respect to the movement of atoms.^51^ When combined with appropriate databases of quantum-mechanical reference data, these ML frameworks were demonstrated to be capable of providing highly accurate interatomic potential models for materials and molecules.

**Figure 11:**
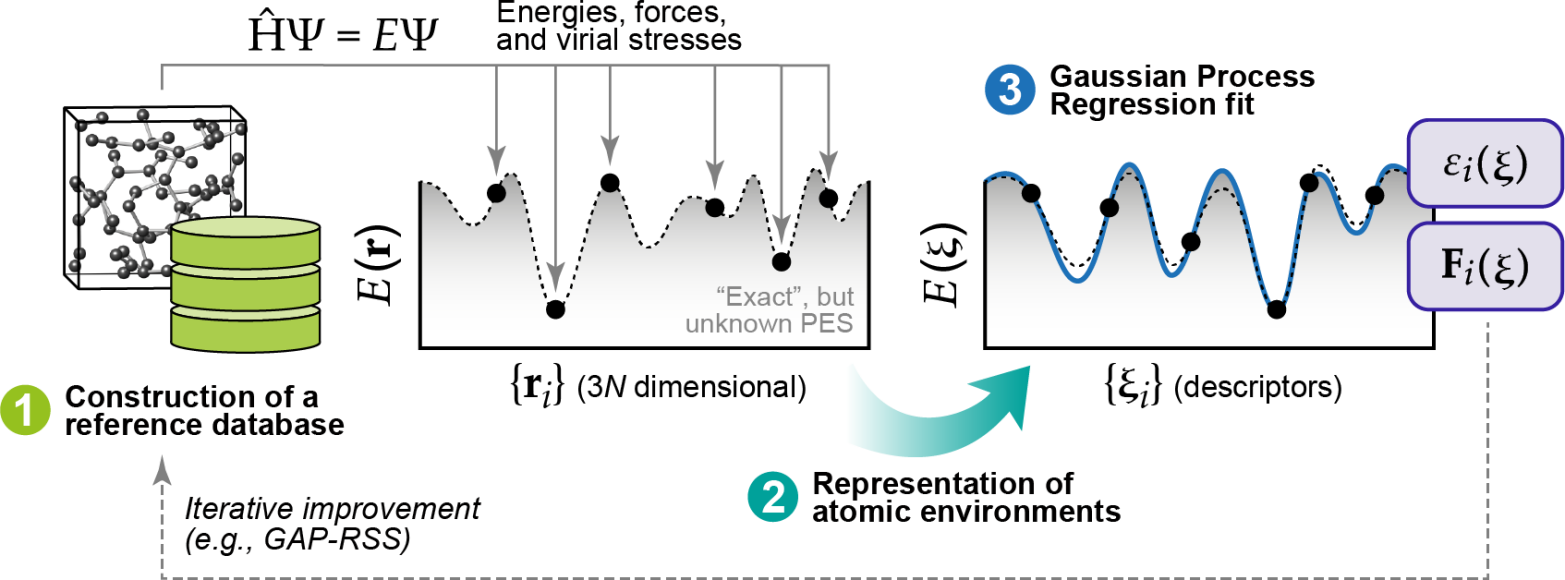
Three main components for GAP: (1) a reference database of quantum-mechanical data for suitably chosen structural models, (2) a representation of the atomic environments (typically using combinations of 2-body and SOAP descriptors; section 4.2), and (3) the GP regression itself. Adapted from ref (29) with permission. Copyright 2019 WILEY-VCH.

In the present section, we review the Gaussian approximation potential (GAP) framework, one of the schemes for generating ML-based interatomic potentials that have recently found widespread use. The software implementation is part of the QUIP code.^98^ Formally, GAP is an application of GPR to infer a decomposition of the total energy of an atomistic system into atomic (“local”) energies, from input data that can comprise total energies and their derivatives (forces and stresses). As with other ML potential fitting frameworks, the three components of GAP modeling are the reference database, the representation of atomic environments using suitable descriptors (including, but not limited to, SOAP), and the regression task itself which is here carried out in the GPR framework (Figure 11). We discuss at some length the methodological choices that we have made in developing and defining this framework, and we explain the reasoning that leads to them.

The following principles guide the construction of GAP models:

- All available data are used: total energy, forces, and stresses (for periodic systems), combined into a single ML fit. The design of the input database is critical to the success of the model and has been a cornerstone of all presently available general-purpose GAPs. The selection of reference data is as much an area of ongoing methods development as is that of representation and regression (section 4.1).
- The choice and specification of structural descriptors (representation) is tightly coupled with the choice of kernels, and both are an essential part of the user input. They incorporate prior knowledge about the nature of the potential-energy function—specifically, its regularity. Commonly used examples are distances and angles between atoms together with a squared exponential (Gaussian) kernel, or the many-body SOAP representation with a polynomial kernel. These are not mutually exclusive: low-dimensional kernel models can be fitted together with many-body ones, with appropriate weighting between them. All representations and kernels in GAP have finite distance cut-offs, typically about 5–6 Å, and therefore they represent the local environments of the atoms (section 4.2).
- Baseline models, determined *a priori*, are used where possible. The baseline could be a certain level of electronic structure (say, we fit the difference between DFT and coupled-cluster potential energies), or an analytical long-range potential, e.g., an electrostatic or dispersion model, or in fact any fast force field or even just a purely repulsive interaction. Hierarchical models, in which multiple fitted potentials are added together, are discussed in section 4.2.
- The atomic energy is written as a sum of a fixed number and type of kernel basis functions, irrespective of the type and exact amount of input data, making the model a sparse Gaussian process. Decoupling the number of input data points, *D*, from the number of basis functions (“representative points”), *M*, makes the prediction cost formally independent of the amount of input data (although in practice a larger *M* may be needed to represent a larger, more diverse training set). Therefore, the storage and cost requirements of using a GAP model scale with the number of representative points, not with the size of its reference database (section 4.3).
- Hyperparameters of the GAP model are chosen and fixed a priori as much as possible and optimized only where required. The main hyperparameters are (i) the relevant length scales, which define the cutoff radius and the smoothness of the kernel, and (ii) the expected errors (arising both from noise in the input data and limitations of the model, e.g., due to the necessarily finite cutoff radius; section 4.4), which determine the regularization of the fit (section 4.6). Practical choices for hyperparameters are discussed in section 4.5.

While the rest of the present section will expand on the details of GAP, we note here briefly that over the past decade, numerous other works have proposed many-parameter fitting schemes inspired by a variety of ML methodologies, blending them with a range of materials modeling approaches. Following the foundational work of Behler and Parrinello,^66^ feed-forward neural networks with a handful of layers are used in the ANI series of force fields for organic molecules,^104,105^ as well as the ænet,^106^ Amp,^107^ DeepMD,^108^ and Panna^109^ implementations, and have even been coupled with charge equilibration schemes.^110^ For more details, ref (111) provides a review in the present thematic issue. Independent implementations of GPR/KRR were also used with SOAP-like features for tests in bulk vanadium hydride^112^ and zirconium^113^ and also to directly predict force vectors rather than the potential energy.^114,115^

### 4.1 Reference Data

The quality of any ML model hinges on the quality of its input data, and interatomic potentials including GAP are no exception. The choice of reference data is particularly important because ML potentials are *nonparametric*: they lack a physically justified functional form, and thus they have enormous variational freedom that must be constrained by the input data.

A range of approaches have been developed for the construction of reference databases. These are primarily guided by the intended purpose of the potential. “General-purpose” potentials are intended to accurately represent a material under a wide range of conditions, whereas others might be fitted for a specific purpose, e.g., to study the transition between specific crystalline phases^116,117^ or the Li-ion mobility of a given compound.^118−120^ In the following, we show some examples of the development of different strategies for building databases, from hand-selected configurations to almost fully automated protocols. In keeping with the scope of the present review, we discuss these strategies in the context of GAP, although many ideas and methodological approaches are transferable to other fitting frameworks.

#### 4.1.1 Hand-Built Databases

**Figure 12:**
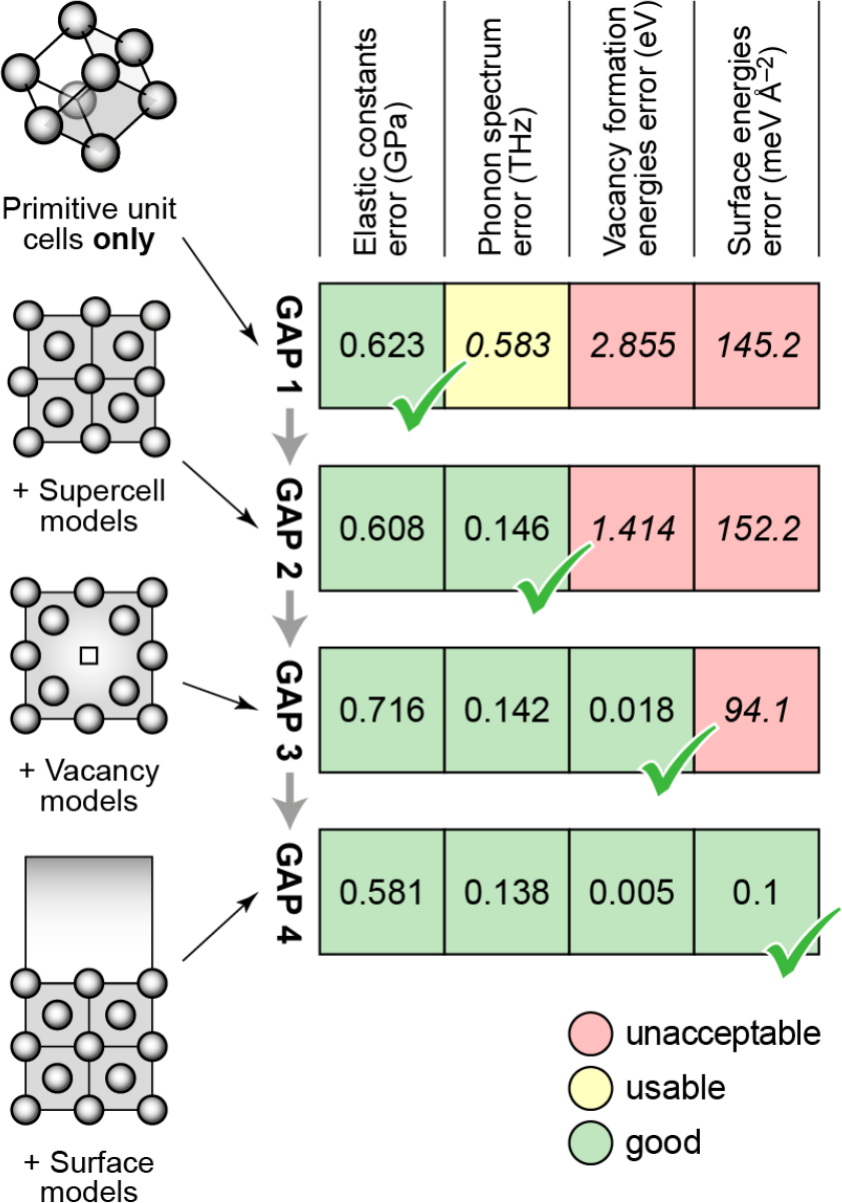
Accuracy of GAP models for tungsten fitted to a series of progressively more comprehensive, manually constructed databases (labeled as “GAP 1” through “GAP 4”). Numerical errors for four different properties are given: if the corresponding type of configuration has been included in the database, the GAP performs well in predicting this property (indicated in green). Figure drawn with data from ref (121) and adopting the color scheme from that work.

Early GAP fitting databases were developed by hand, using physical intuition to select relevant configurations. Among the first examples was a GAP for elemental tungsten, which was designed to describe the material in its ambient body-centered cubic (bcc) crystal structure with relevant low-energy defects, including vacancies, surfaces, generalized stacking faults, and dislocations.^121^ The fitting proceeded in stages, starting from a narrow range of configurations and gradually adding more structurally diverse ones (Figure 12). Initially, the GAP was fit only to snapshots representing the bulk bcc phase with small perturbations, and consequently it was accurate only for properties that depend exclusively on such geometries, such as elastic constants and phonon frequencies. Configurations with very different atomic environments, such as defects, had much larger errors in predicted energy because they had not been “shown” to the fit. As increasingly diverse configurations were added to the fitting database, the applicability range expanded: at each stage, adding configurations representing various defects improved the model prediction results for that defect type, without appreciably worsening its accuracy for the configurations considered at a previous stage. This desirable behavior is a reflection of the variational freedom of GAP, its locality in atomic-environment space, and the stability of GPR: fitting in additional regions of configurational space does not necessarily change the behavior for previously fit regions. Some care has to be taken to achieve this; for example, the number of representative configurations might need to be increased.

The design of a fitting database for a GAP must take into account the risk of unphysical predictions for structures that are far from the fitting configurations, due to its large variational freedom and the lack of constraints from built-in physics beyond symmetries and smoothness. A potential with only low-energy configurations in the fitting database may not correctly predict the true increase in energy at the boundary that separates the physically reasonable regions from inaccessible, high-energy configurations. Using such a potential in a configuration-sampling method, such as MD, may therefore cause the system to evolve into unphysical regions of configuration space. Thus, in order to obtain a usable potential, it is essential to fit not only the configuration-space region of ultimate interest but also its “boundary”. Note that the dimensionality of configuration space could make this a challenging task—even when the *n*-dimensional volume of interest can be adequately sampled, if the required boundary has comparable length scale, its volume would be of order 2^*n*^ times larger.

A practical way to address the requirement to fit the boundary is to make the fitting process iterative. A proposed potential is used in an MD or Monte Carlo sampling of configurations at conditions that are more extreme than those of interest (e.g., higher temperatures or a wider range of pressures). Configurations that will improve the fit must be identified and evaluated with the reference method for inclusion in the fitting database. The GAP is refit with the additional configurations, and the process is repeated until no more unphysical behavior is seen.^122^ Variance prediction can provide a useful tool to identify poorly predicted configurations for fitting (sections 4.1.2 and 5.2), although it has not been widely used for GAP model development so far.

#### 4.1.2 Iterative and Active Learning

One important contrast that we would like to draw is between what we describe above as iterative fitting and what is often referred to as “active learning” in the ML community. In iterative fitting, we add more fitting data points at each iteration, and convergence is determined by the performance of the model on some independent and physically meaningful property. The challenge is then to select the best (most informative) fitting data to add at each iteration and to develop a convergence test that ensures that the resulting model is sufficiently accurate and robust for future application. The goal is to approach a stable, “converged” potential, which can then be used in practice without having to continually refine it further. In the next subsection, we give an example of such a procedure with configurations generated by random-structure search.

Active learning, on the other hand, depends on the ability to efficiently *predict* the accuracy of the model for each configuration as it is generated during a simulation, for example using the predicted variance for a GP,^61,123−125^ D-optimality for a moment tensor potential (MTP),^126,127^ or model ensemble variation for a neural network.^128,129^ Details on how to obtain such error estimates are given in section 5.2.

When configurations that are expected to be poorly described by the existing model are encountered, they are evaluated using the reference method and added to the training set, and the model is refit. In practice, active learning is often used without the goal of developing a single general-purpose potential that describes the material under all conditions, but rather one that ends up being tailored for a specific simulation (material, crystal structure, temperature range, etc.).^124^ The process converges when a particular simulation stops producing configurations that are considered novel enough to be added to the training set—this may or may not be reached in practice.

Active learning was first proposed for interatomic potentials in the context of neural networks,^128^ where it was successfully applied to MD simulations of Cu bulk and surfaces. It is still being used in neural-network models with more complex architectures, for example in the development of “deep potential” models for Al–Mg alloys.^129^ In that work, the active learning loop was added to MD simulations of temperature ramps starting from known crystal structures at low temperatures and increasing to values above the melting point. The resulting models reproduce not only the PES sampled by the simulation but also structural properties such as the liquid radial distribution function, as well as energies of configurations that are unlikely to be represented in the MD trajectories such as free surfaces. Active learning in the context of reference-data selection for GAP was demonstrated for liquid and amorphous phases of hafnium dioxide^130^ and very recently coupled with experimental observations into a fitting workflow for this material.^131^

**Figure 13:**
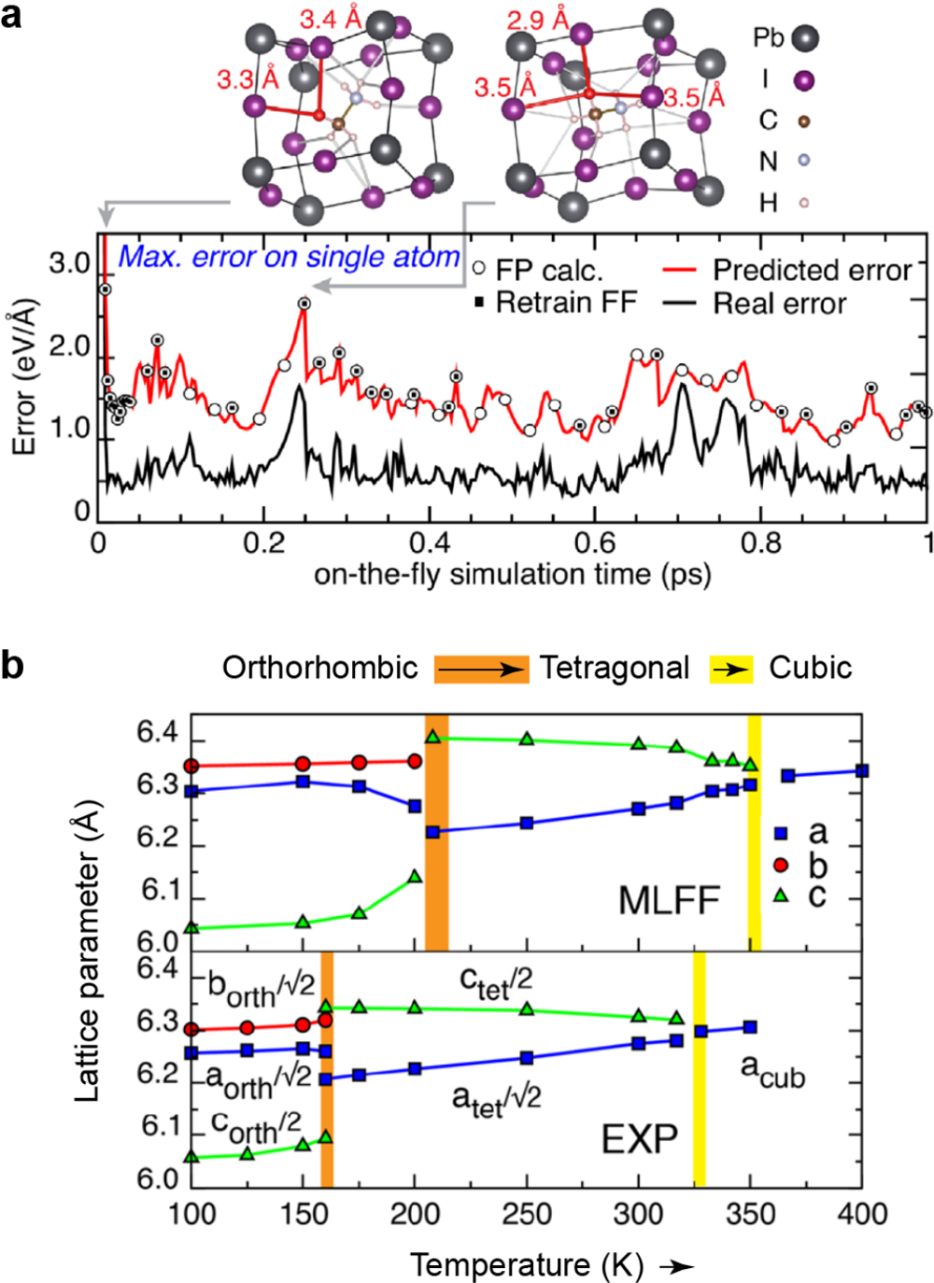
Fitting of a GPR-based ML potential as fully integrated with *ab initio* molecular dynamics. (a) Time evolution of measured and predicted force errors in an on-the-fly fitted GPR model for the methylammonium lead iodide (CH_3_NH_3_PbI_3_) hybrid perovskite during an MD simulation. (b) Lattice parameters of this material as a function of simulation temperature for the GPR model (MLFF), as compared to experiment (EXP), showing structural phase transformations as indicated by the orange and yellow vertical bars. Reprinted with permission from ref (123). Copyright 2019 by the American Physical Society.

The developers of the VASP first-principles simulation software^132^ integrated an automated GPR-based potential using active learning as a technique for accelerating their simulations.^123−125^ Using SOAP descriptors but slightly different expressions for regression than GAP, and using the GP to predict variances of forces and stresses as well as energies, they showed that predicted variances are good proxies for actual error, as shown in Figure 13a. Although a rescaling was required to bring them into quantitative agreement with the actual error, the predicted variances were effective for use in selecting fitting configurations for active learning. The authors applied their methodology to a wide range of systems, including metals, AB_2_ Laves phases, and hybrid perovskites: for example, Figure 13b shows the evolution of the lattice parameters of methylammonium lead iodide (CH_3_NH_3_PbI_3_), during the orthorhombic to tetragonal to cubic transitions, as compared to experimental results. This material has been widely studied with DFT.^133^ Tong et al. used a similar predicted-variance criterion for active learning of configurations during the search for low-energy structures of large boron clusters,^134^ culminating in the prediction of a new ground state structure for B_84_. The VASP code with this built-in SOAP-GPR-based acceleration technique has since been used by other groups, e.g., to study the atomic-to-electride liquid–liquid phase transition of potassium.^135^

Vandermause et al. employed GPR variance prediction to drive an active learning procedure for an interatomic potential, although they used two and three body descriptors, rather than SOAP.^61^ While this choice of descriptors led to a somewhat higher error relative to their reference data, the authors were able to map the resulting potential to a spline form for greatly increased computational speed. The method was applied to melting and point defect diffusion in aluminum, as well as a wider range of materials (metals, semiconductors, metal oxides) at a narrower range of temperatures (and therefore of geometries). In this case, the hyperparameters of the GP were optimized by maximizing the marginal likelihood, and it is likely that this is a key component of accurate error predictions. In fact, the dependence of the variance prediction on the fitting data values (not just fitting data *locations*, i.e. the geometry of the configurations) is only through this optimization—the predicted variance expressions themselves are only explicitly dependent on kernels between input configurations.

Finally, MTPs have been presented as part of an active learning loop explicitly based only on the volume of the input data space spanned by the training dataset, rather than explicitly predicting the error in the output,^126,127^ although these are related through the idea of D-optimality. The procedure was first applied to simple metals in the solid and liquid phase and later showed success in the much more complex and geometrically diverse process of structure search in a wider range of materials, including metals, semiconductors, and insulators.^127^ Several other applications of active learning to other types of interatomic potentials are listed in a recent overview.^125^

#### 4.1.3 GAP-RSS

The majority of ML potentials has been developed based on a knowledge of the relevant atomistic structures: crystalline phases added to the reference database by hand; liquid and amorphous structures taken from first-principles MD simulations; specified structures that serve as the starting point for “on-the-fly” potential fitting. These potentials are accurate in the sense that they reproduce the energetics of crystalline phases to within a few millielectronvolts per atom and often a range of other relevant properties (Figure 12). They can also be flexible enough to drive a global search for crystal structures that would normally be carried out with DFT—for example, in the widely used *ab initio* random structure searching (AIRSS) approach by Pickard and Needs.^136,137^ In the context of GAP, the ability to carry out structure searching successfully has been demonstrated for carbon^138^ and silicon,^139^ identifying low-enthalpy minima and describing the density distribution of energies in good agreement with DFT.

In the present section, we review a method for the *de novo* exploration and fitting of potential-energy surfaces *without* the prior inclusion of any known structures. Starting from randomized configurations, a GAP is fitted and used to carry out structure searching; the resulting minima are labeled with DFT and fed back into the training; the process is then repeated until convergence. We refer to this method, combining GAP fitting and random structure search, as “GAP-RSS”,^140^ in analogy to AIRSS. Here, we focus the presentation on GAP, in keeping with the scope of the present review article, but we note that other ML fitting schemes have also been successfully combined with different structure-searching techniques.^126,134,141^

Whether such a *de novo* approach would work at all is not obvious: in fact, AIRSS and related methods start from randomized structures that are highly dissimilar from experimentally known phases, and therefore, the exploration especially of the higher-energy regions of the PES requires sufficiently accurate energy and force evaluations, normally afforded by DFT. Why, then, would an ML potential find new lower-energy structures to which it has not been fitted and which it therefore describes rather poorly? The key is that the potential does *not* have to be accurate for a low-energy structure in order to find it: the combination of large structural diversity generated by the random-search algorithm and *sufficient smoothness* (of both the DFT potential-energy surface and the GAP fit) allow the potential to explore lower-energy regions in subsequent iterations, eventually converging to a good description of the PES.

The central idea behind GAP-RSS, namely that of starting with randomized atomic configurations and coupling fitting and exploration, was introduced in ref (140). The test case in that work was elemental boron, which is challenging because multiple structurally complex allotropes exist and need to be correctly described by the method, and even the simple α-rhombohedral structure is based on B_12_ icosahedra (see section 6.2). The search started from random configurations, created using the buildcell functionality of the AIRSS code,^137^ for which DFT reference data were computed and an initial GAP was fit. From searches (that is, structural relaxations) using this initial potential, structures were taken after 5 and 200 relaxation steps, corresponding to RSS “intermediates” and configurations closer to local minima, respectively. Iterative DFT computations, potential fits, and searches with the next potential version led to progressively improved GAP models, quantified using the energy error for the bulk allotropes which the potential had not initially “seen”. Of course, once the bulk structures *were* added, their description was improved much further. This initial work also explored the role of GAP atomic energies, showing that for a supercell model of β-rhombohedral boron with the relevant crystallographic sites all fully occupied, high (unfavorable) atomic energies are predicted for the B13 site that experimentally show a partial occupation; see ref (140) for details.

The approach was then expanded by a *selection step* in subsequent work, which focused on phosphorus as a test case:^142^ rather than feeding back all configurations in a given iteration, only the most favorable ones were selected. In this case, the criterion was that all atoms in a given structure needed to be 3-fold-connected,^142^ in accord with the crystalline allotropes of phosphorus and its location in the fifth main group of the Periodic Table. Indeed, in this study, the orthorhombic structure of black phosphorus was “discovered” after a few iterations, and once the corresponding snapshot had been fed back into the database, the energy–volume curve was brought into good agreement with DFT.^142^ The work furthermore explored GAP-RSS searches at high pressure, in this case showing how the As-type and simple-cubic allotropes can be recovered.^142^

Subsequently, for elemental systems, this process has been automated using general heuristics for the hyperparameters, RSS process, and structure selection criteria, so that only the chemical element needs to be specified.^143^ In this case, a length scale is set from a tabulated characteristic elemental radius (metallic or covalent) and a volume scale that is derived from this length scale and the geometry of typical open-network (covalent) or close-packed (metallic) structures. The length and volume scales are used to set all spatial hyperparameters, including the potential cutoff distance, SOAP smoothness *σ*_*a*_, and RSS initial structure density and minimum interatomic distance. In the initial step, a set of 10^4^ random structures is generated and 100 are selected for maximum diversity using leverage-score CUR,^144^ similar to that used for the selection of representative atomic environments in GAP fitting (section 4.3). In this case, the CUR algorithm is applied to the “average SOAP descriptors” that describe an entire structure by a single power-spectrum vector, built from coefficients corresponding to the local environments.^145^

**Figure 14:**
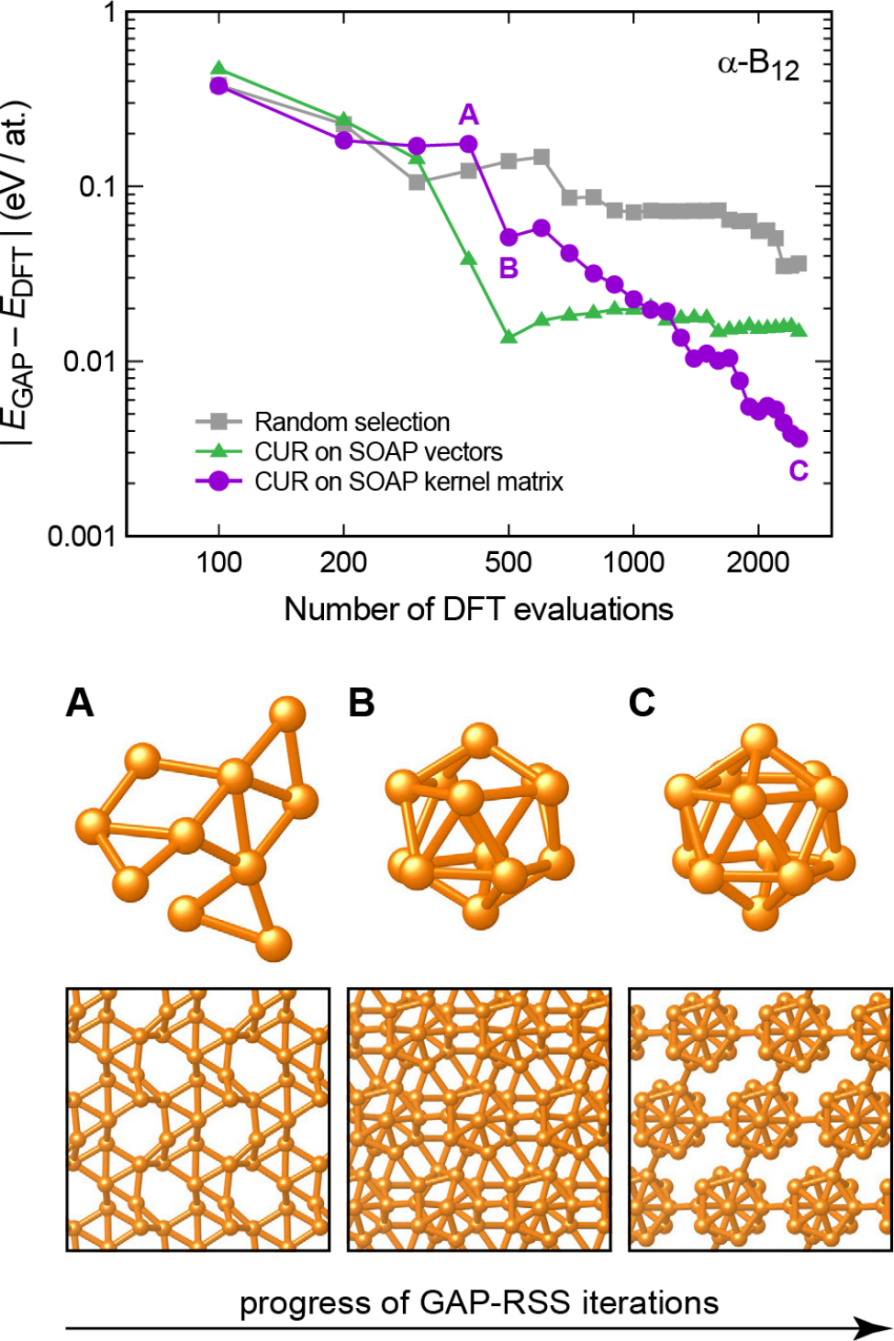
Exploring and fitting structural space from scratch with the GAP-RSS methodology.^140,143^ The example shown here illustrates how the structure of α-rhombohedral boron is discovered within a few iterations of GAP fitting and iterative random structure searching. Reprinted from ref (143). Original figure published under the CC BY 4.0 license (https://creativecommons.org/licenses/by/4.0/).

As in ref (140), an initial GAP is then fit to DFT reference value energies, forces, and stresses for the selected configurations. In the methodology of ref (53), for each subsequent iteration, the GAP from the previous iteration is used to find RSS minima from 10^4^ initial random configurations, with the minimization of enthalpy under a random pressure from a user-defined distribution. First, a set of relevant minima is selected with a two-step process. A Boltzmann-biased flat histogram in enthalpy is used to select a few thousand minima, to ensure that the set is independent of the probability density of the RSS minima population (through the use of the flat histogram) and biased toward low-enthalpy configurations (through the Boltzmann weight). A diverse subset of these minima is selected using CUR, as in the initial step, and the entire set of minimization trajectory configurations leading to these minima is used as a pool for the fitting configuration selection. From this set, 100 configurations are selected using the same flat-histogram and CUR process, evaluated with DFT, and added to the fitting database. This process ensures that the fitting database focuses on a wide range of diverse local minima as well as higher-energy configurations that might be encountered during a simulation; it retains the advantage of selection by CUR on the kernel matrix (purple in Figure 14) and avoids the computationally expensive task of computing the kernel matrix on the entire large set of configurations generated by the RSS minimization process (10^4^ trajectories with about 100 steps each).

The evolution of the GAP-RSS process is shown in Figure 14. With each iteration, the accuracy of the GAP prediction (compared to the reference DFT value) for the DFT minimum α-B_12_ structure improves, with the best convergence seen with the use of CUR on the descriptor kernel matrix. The evolution of the corresponding RSS itself is shown in the bottom panel of the figure, in the form of visualizations of fragments and plan views of the respective lowest GAP-energy structure found. Even the fourth iteration finds a structure with many three-membered rings, which are important in several low-energy B crystal structures. Subsequent iterations find structures that become increasingly close to the nearly ideal icosahedra in the DFT minimum energy structure.

The iterative combination of structure search and fitting has not been restricted to AIRSS and GAP: in fact it can be done with any combination of methods, in principle, as noted in ref (138). An evolutionary structure searching approach, implemented in the USPEX code,^146^ was combined with moment-tensor potentials (MTP) to accelerate the structure search process for a number of elements.^127^ Described as a way of accelerating the discovery of new crystal structures, the combination was successfully applied to C, Na under pressure, and B. The structure-search algorithm combined with the computational efficiency of the moment tensor potentials (MTP) enabled the construction of several 105–108 atom approximants of the β-B structure, which is highly complex with many partly occupied sites. In terms of nanostructures, it was shown, for example, how the fitting of a neural-network potential can accelerate evolutionary searches for the structures of nanoparticles on surfaces.^147^

Another algorithm, viz. crystal-structure searching by particle-swarm optimization^148^ as implemented in the CALYPSO software,^149^ was combined with a GAP model (using atom-centered symmetry functions rather than SOAP descriptors) to iteratively search for structures and refine the GAP.^134^ In one variation active learning was used, selecting configurations to be added to the fitting database based on predicted error from the variance of an ensemble of GAP models. The generated GAP models were shown to be effective for CALYPSO searches, and they were used to predict a new ground-state structure for the B_84_ cluster; examples of this search and others will be discussed in section 6.2. As stated above, presumably any ML potential could benefit from similar approaches, as long as the potential can take advantage of smoothness or other physical properties of the PES to have sufficient transferability to reproduce (at least semiquantitatively) the diverse range of configurations that appear in a random structure search.

#### 4.1.4 Automatic Training Set Selection

A common problem one encounters is that of extracting from a large set of configurations—for instance obtained from exploratory ab initio molecular dynamics or from simulations performed at a lower level of theory or with an empirical force field—a smaller set of configurations that exhibit maximum diversity, to be recomputed with a more accurate method, or just to discard redundant configurations to accelerate the fitting procedure. Both farthest-point sampling^150^ (FPS, a greedy algorithm that selects at each stage the structure that is most different from those that have been selected already) and CUR decomposition (a factorization that uses columns and rows of a matrix to approximate it) have been used for this task.^72,143,151,152^ Whenever the regression target, or an inexpensive approximation of it, is available for the large dataset, it is possible to use it to improve the quality of the selection, either with genetic algorithms^153^ or with extensions of FPS and CUR techniques^154^ inspired by principal covariate regressions.^155^

#### 4.1.5 General-Purpose Databases

**Figure 15:**
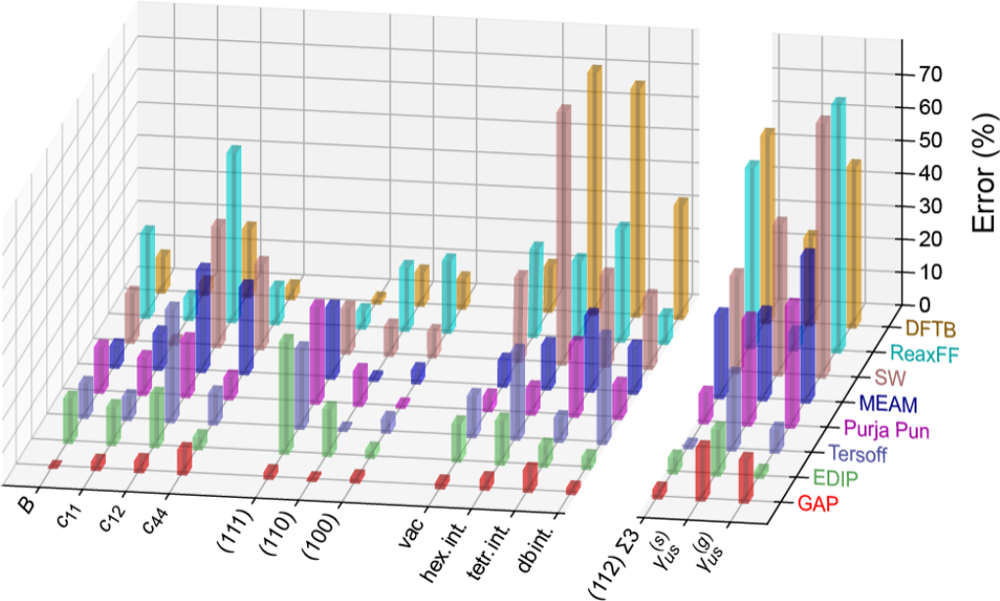
Accuracy of the general-purpose silicon GAP.^139^ The bar chart shows the percentage error of some basic material properties and the formation energy of selected defects with respect to DFT: elastic constants (*B*, *c*_11_, *c*_12_, *c*_44_); surface energies for the (111), (110), and (100) surfaces; vacancy (“vac”) and interstitial (“int”) formation energies in the hexagonal (“hex”), tetrahedral (“tetr”), and dumbbell (“db”) configurations. While the local environments relevant to the properties on the left side of the figure are well represented in the database, the (112)Σ3 symmetric tilt grain boundary and unstable stacking fault energies on the shuffle (γ_*us*_^(*s*)^) and glide (γ_*us*_^(*g*)^) planes, on the right of the figure, are not and therefore indicate a degree of transferability to new, unseen properties. Also shown are the errors of a number of empirical potentials: EDIP,^156^ Tersoff,^157^ Purja Pun,^158^ MEAM,^159^ SW,^160^ ReaxFF,^161^ and DFTB.^162^ Although some of these have not been fitted to DFT data for the relevant configurations, and sometimes not to any DFT at all, the variance between values obtained with different flavors of DFT (and even with experiments) for the properties shown is typically less than the errors of the empirical potentials. Reprinted from ref (139) under the CC BY 4.0 license (https://creativecommons.org/licenses/by/4.0/).

General-purpose ML potentials aim to describe a material under all reasonable conditions, including a diversity of phases, surfaces, relevant defects, etc. They require general-purpose databases that cover all this wide variety of local environments. The defining attribute of such a potential is that it can be used by other researchers, not involved in its construction, sometimes for new purposes that were not envisaged when the fitting database was assembled. The first such database was painstakingly built by hand using a combination of chemical intuition and “trial and error” for silicon,^139^ leading to a database that contains over 170,000 atomic environments. The GAP model fitted to this database provides near first-principles accuracy for a wide variety of properties. This is illustrated by the bar chart in Figure 15, showing the percentage errors with respect to DFT for a number of simple material properties, in comparison to several empirical potentials available for silicon. Beyond these, the GAP gives an accurate description of vibrational modes, thermal expansion, dislocations, and crack tips and complex surface reconstructions for diamond-type silicon, the equations of state for various relevant crystalline phases, and the structure of amorphous and liquid silicon.^139,151^ It has recently been used in a large-scale simulation to shed light on the behavior of amorphous silicon under high pressure, which we discuss at the end of this review.^164^

**Figure 16:**
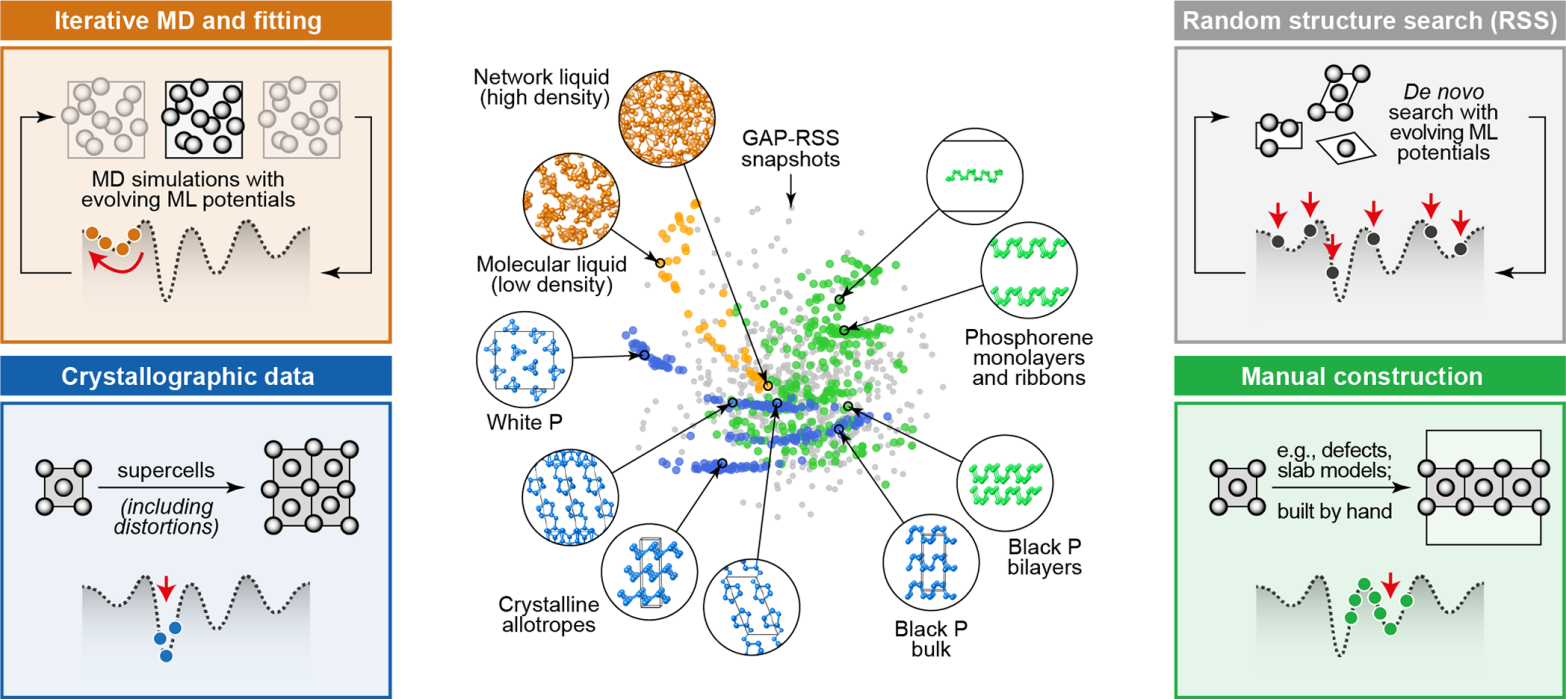
Different strategies for constructing reference databases for ML potentials, indicated by cartoons in the boxes. The center of the figure shows a database of phosphorus configurations used to fit a general-purpose GAP for this element. The structural map, visualizing the (dis-) similarity between different configurations, illustrates the connection between random structure search (gray), exploration with the potential using MD (orange), and manual database building (blue, green). Adapted from ref (163). Original figure published under the CC BY 4.0 license (https://creativecommons.org/licenses/by/4.0/).

The amount of manual work that was required to assemble the silicon database is clearly not sustainable if similar general-purpose potentials are to be developed for a wider variety of materials. Figure 16 combines several of the ideas discussed in the present section into a blueprint for making general-purpose potentials. The example case, shown at the center of the figure, is elemental phosphorus, a structurally highly complex system with multiple low-energy crystalline polymorphs: see, for example, ref (165) for the synthesis and characterization of monoclinic “fibrous” red P and ref (166) for a computational survey of the different allotropes. Phosphorus is also of application interest in terms of monolayers (“phosphorene”; ref (167)) and, more recently, nanoribbons^168^ derived from the layered structure of black P. This structural diversity, together with the need to describe certain regions of the PES highly accurately (in this case, for example, the exfoliation curve of phosphorene), places demands on the construction of the reference database that is used in the potential fit. The database developed in ref (163), for which a SOAP-based structure map is shown in the center of Figure 16, aims to achieve this goal. On the one hand, it enhances transferability by including a highly diverse set of structures from an earlier GAP-RSS search,^142^ and on the other hand, it ensures application relevance by including carefully chosen configurations that are relevant to specific physical problems that might be studied: here, for example, the description of phosphorene nanoribbons, which have been synthesized recently.^168^

### 4.2 Hierarchical Models

Having discussed the development of reference databases in some detail, we turn now to the other aspects of the GAP methodology which are concerned with the fit itself. While it is certainly possible to fit an interatomic potential using GPR and a many-body kernel such as the SOAP (eq 57) on its own, we suggest that this is almost always a bad idea. The reason is that there are at least two distinct energy and length scales in potential-energy surfaces: the attractive regime of interatomic bond formation on the length scale of Ångströms and energy scale of electronvolts (hundreds of kJ mol^–1^) and the repulsive regime between nuclei (including electronic exchange repulsion) on the length scale of tenths of Ångströms and energy scale of tens of electronvolts and higher. In most applications, we are interested in a detailed and accurate description of the former and just a rough approximation of the latter (one exception to this is the study of high-energy impact events, which will be reviewed in section 6).

We can augment the many-body model with low-body-order terms (as in eq 52), which are themselves fitted at the same time as the many-body model. It is convenient to retain the linear algebra framework of the kernel regression method, and this can be done if all the terms which we wish to fit are expressed as GPR models. All we need to do to achieve this is to define suitable descriptors and kernels for each term and use them in the “linear functional observations” framework introduced in section 2.4. For the pair potential, the distance between two atoms is the canonical choice. For the three-body term, either two distances and an angle or three distances are equally suitable. In both cases, permutational symmetry must be enforced, either by symmetrizing the descriptor or by summing the potential term over the permutation group of three particles (depending on the three element identities). The total energy expression of a combined two-body and many-body model, using Gaussian kernels for the two-body terms, is then
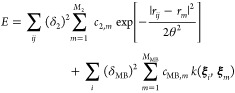
74where we have introduced weights δ_2_ and δ_MB_ for the two terms, which scale the relative contributions of the different terms and have units of energy. (Because kernels are unitless and the coefficients *c* have units of inverse energy, each term on the right-hand side has appropriate energy units.) The two-body term is a one-dimensional sparse GP with *M*_2_ representative points located at the interparticle distances *r*_*m*_, which in practice we often take to be a regular grid up to some cutoff. In this formulation, the different terms are not independent: a general many-body term of course can describe any two-body interaction too, but not efficiently, since it is intrinsically high-dimensional. So it is only in combination with the regularization of the fitting coefficients and specifying different weights, by using different δ prefactors, that we obtain the benefit of separating out these terms. (Note that it is actually possible to separate out the two-body contributions from the many-body SOAP descriptor explicitly.^169^)

**Figure 17:**
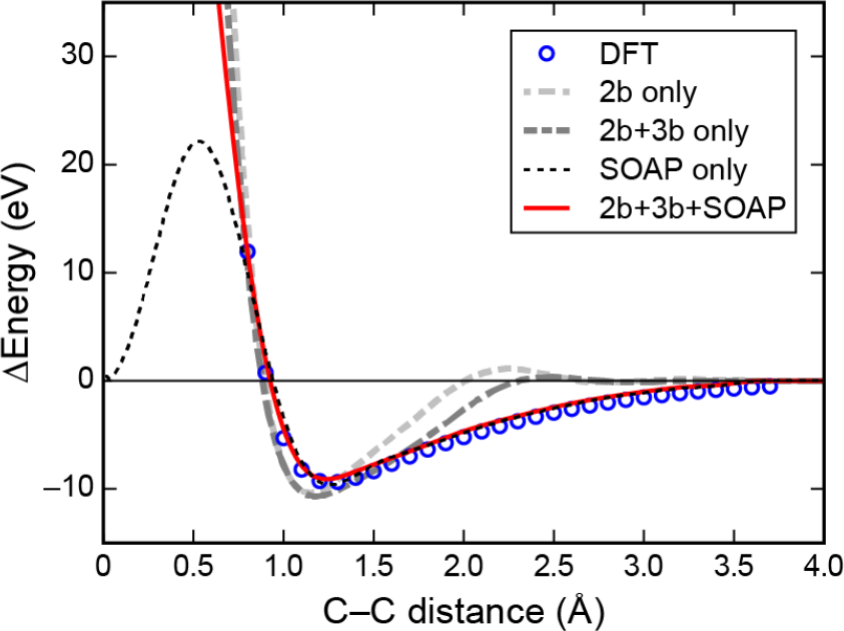
Hierarchical combination of different descriptors in GAP fitting. The figure shows a potential-energy scan for a carbon dimer in the gas phase, evaluated with different GAP models that have been fitted to a large database of bulk, surface, and dimer configurations (lines).^122^ DFT-LDA data for the dimer are shown as reference (blue circles). A model with just a many-body SOAP term (black dashed) matches the DFT dimer data well but has an unphysical local maximum at around 0.6 Å, whereas the 2-body (2b, light gray), combined 2-body and 3-body (2b+3b, dark gray), and a model with 2-body, 3-body, and a SOAP term (red) all extrapolate to high energies for small distances, with the last one also accurately reproducing the data. Reprinted with permission from ref (122). Copyright 2017 by the American Physical Society.

Figure 17 illustrates the trade-off between robustness, flexibility, and overall quality that is linked to the choice of descriptors or combinations thereof, here shown for the example of carbon.^122^ Increasingly complex models, viz. 2-body, 3-body, and many-body (SOAP) terms, capture the potential energy increasingly well, albeit requiring higher computational cost. A pure SOAP model (dashed black line) reproduces well the region where data are available but fails notably at very small interatomic distances. In contrast, the combined 2b+3b+SOAP model (red line) correctly captures the repulsion at very small interatomic distances and therefore is robust even in MD simulations of liquid carbon at 9,000 K (details may be found in ref (122)).

**Figure 18:**
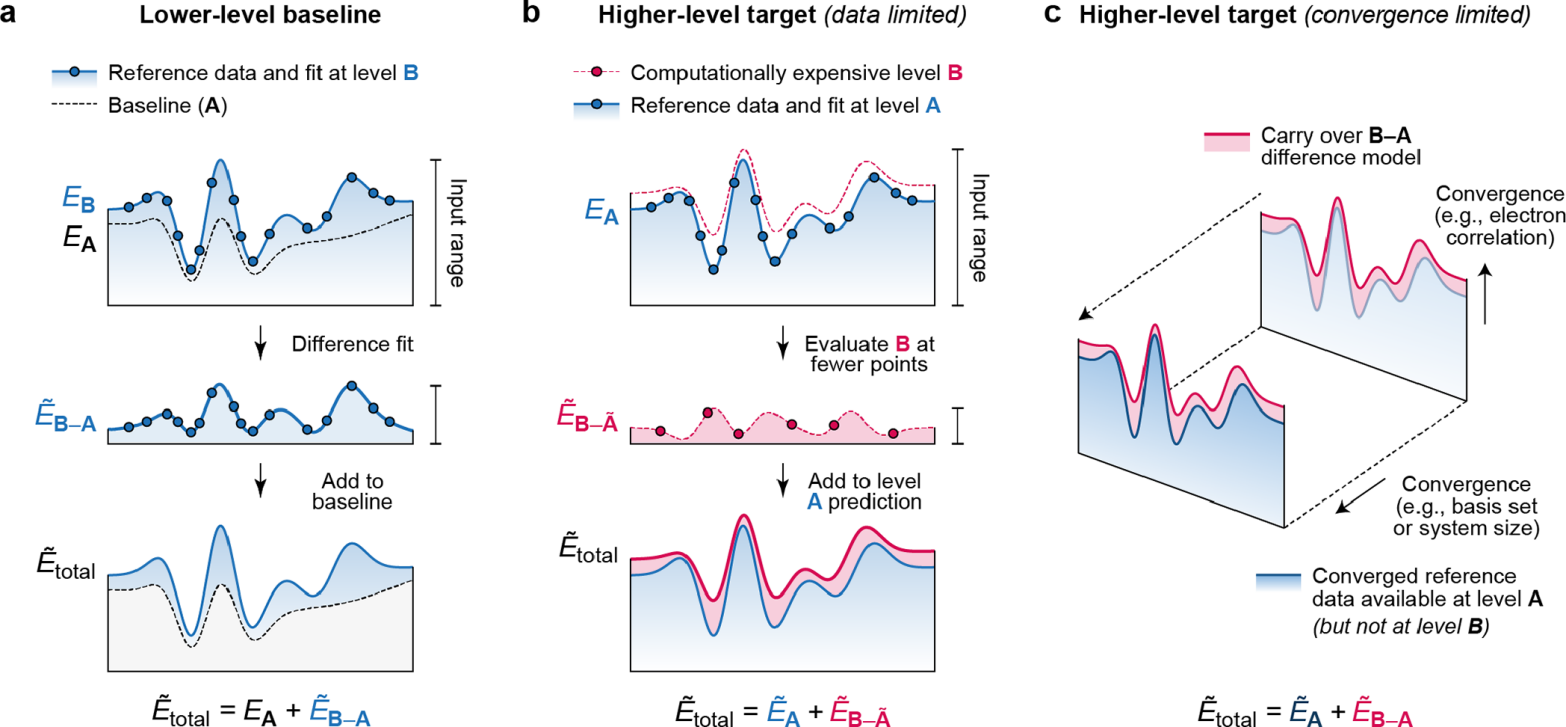
Overview of different approaches to the hierarchical fitting of potential-energy surface (PES) models. In this figure, the actual PES are labeled *E*; fitted models are labeled ; the indices **A** and **B** refer to different types of PES. Drawings are based on the presentation in ref (164). (a) Using a lower-level baseline model, which might be a simple analytical term that only describes certain aspects of the PES (e.g., pair repulsion, fixed-charge electrostatics, or London dispersion) or a fast semiempirical method. The baseline model is subtracted from the reference data before the fit, resulting in a difference model, _**B**–**A**_, to which the baseline model *E*_**A**_ is then added back when predictions are made. (b) Fitting a higher-level target: for a suitably chosen baseline, the difference fitting target is smoother (e.g., the range of input data is smaller, or the difference target varies on a larger length scale), and therefore fewer reference points are required. Here,  in the subscript of  indicates that the fit was made to a potential-energy difference where the fitting target was obtained by subtracting a *fitted model* of PES **A** from the actual PES **B**. (c) A more complex setup in which convergence (e.g., with basis set or system size) can be achieved for level **A** but not for **B**, which might be because **B** uses a higher level of treatment for electron correlation and therefore is more computationally costly.

An alternative way to describe core repulsion is to employ a simple analytic pair potential, *V*_2_(*r*), as a *baseline* that is constructed to be repulsive.^139^ This is data efficient, because less effort is spent collecting data and fitting configurations where *only* two atoms in a large structure are close to each other. There are other cases too in which a simple baseline model outside the GPR framework looks very advantageous, e.g., adding a fixed-charge electrostatic model^170,171^ or a 1/*r*^6^ pair potential to describe the long-range part of (van der Waals or London) dispersion.^152,163,172^ The energy expression to be fitted is then the sum of the fixed pair potential and the many-body term that depends on the many-body descriptor for each atom, **ξ**_*i*_,
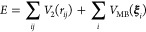
75The training of such a hybrid model is identical to that of a pure many-body model—except that the energy, forces, and stresses of the pair potential are first subtracted from the input data, and the *difference* is then fitted by the ML model, rather than the total potential. The central idea is sketched in Figure 18a, with the baseline model denoted by the letter **A** and the ultimate target of the potential by the letter **B**. The baseline does not have to be as simple as a pair potential. Using a polarizable electrostatic force field as a baseline to augment a short-range many-body ML model also fits into this category.^173^

The baseline could be even more complex, e.g., when the target of the fit is the energy difference between *two different electronic-structure methods*.^419^ These can differ in their treatment of electron correlation (e.g., DFT versus wave function methods) or basis set (e.g., the minimal basis set of tight-binding or LCAO methods versus the complete basis set limit). Although formally this type of modeling does not differ from using a simple analytic baseline, in practice the hyperparameter choices for the fitting can be rather different. This is because the simple analytic potentials are used as a crude estimate of the energy for configurations that are not well covered by the dataset or interactions that are not described by the finite-range many-body model. In contrast, even approximate electronic-structure methods are expected to give a rather good description of the total energy (in an absolute sense) for all configurations. The ML model which is added on top is used to capture delicate details of the potential, fractionally much smaller than the binding energy, perhaps also varying on a longer length scale than the typical Ångstrom scale of covalent bonding. These differences in turn affect how one chooses the descriptors and hyperparameters of the ML model.

The use of an electronic-structure method as a baseline can lead to a combined model whose total computational cost is dominated by that of evaluating the baseline. Such models are not force fields but can be thought of as “corrected” or “enhanced” versions of electronic-structure methods, and depending on the application, such models can be highly effective. An early example of such an ML correction was used to obtain an accurate description of bulk liquid water (with respect to the experimental oxygen radial distribution function and the diffusivity), based on a DFT baseline, corrected with a GAP model for each pair of water molecules fitted to the difference between DFT and CCSD(T).^174^ See also section 6.6 for a more recent example, fitting the difference between DFTB and DFT for organic crystal-structure prediction.^175^ (A completely different way of using reference data on multiple levels of electronic-structure theory is in ref (176) where the electron density is used as an intermediate “descriptor” in improving DFT energies to CCSD(T) level.)

A variation on the difference fitting approach is illustrated in Figure 18b. Here, the baseline model **A** is also fitted by an ML model, perhaps using a much larger dataset afforded by the lower cost of evaluating model **A** in comparison with **B**. When the database for the difference fit is constructed, **Ã**, i.e. the fitted model for **A**, is subtracted from **B**. A more systematic study of many “difference models” on top of each other, capturing each intricate term (with cm^–1^ or 0.1 meV accuracy) separately in a perturbative wave function approach, was used to significantly reduce the total cost of building the reference database of electronic-structure calculations for the CH_3_Cl molecule.^177^

Figure 18c illustrates a more complicated setup, in which again two levels of theory are used for reference calculations (e.g., with different treatment of electron correlation), but also some other aspect of these calculations needs to be converged (e.g., the basis set employed). Here, a database and a corresponding ML model is created with the lower level of theory, **A**, and a high level of basis convergence. To this, a second ML model is added, which is fitted to the difference between method **A** and **B** calculated at a *low* level of basis convergence—because a high level of convergence is unfeasible using the more expensive method, **B**. This approach was used in ref (174) for modeling water dimers, the two levels of theory being MP2 and CCSD(T), and also in ref (164) for silicon where the two levels of theory were DFT and RPA.

The latter case is an example from materials modeling, where the limitation due to computational costs associated with model **B** was not the basis set employed but rather the system size. Large amorphous silicon structures were described at the DFT level (**A**) based on reference configurations of up to 216 atoms per unit cell, whereas the structures used for constructing the correction up to the RPA level (**B**) contained only 16 atoms per cell at most. The latter structures would certainly not have been sufficient on their own to create a stand-alone fitting database capable of accurately describing amorphous silicon; however, they suffice for constructing the difference model. The small structures were taken from a GAP-RSS database (ref (143)), thus illustrating the usefulness of random structure search for generating structurally diverse yet computationally feasible reference data for ML potential fitting in a variety of contexts.

**Figure 19:**
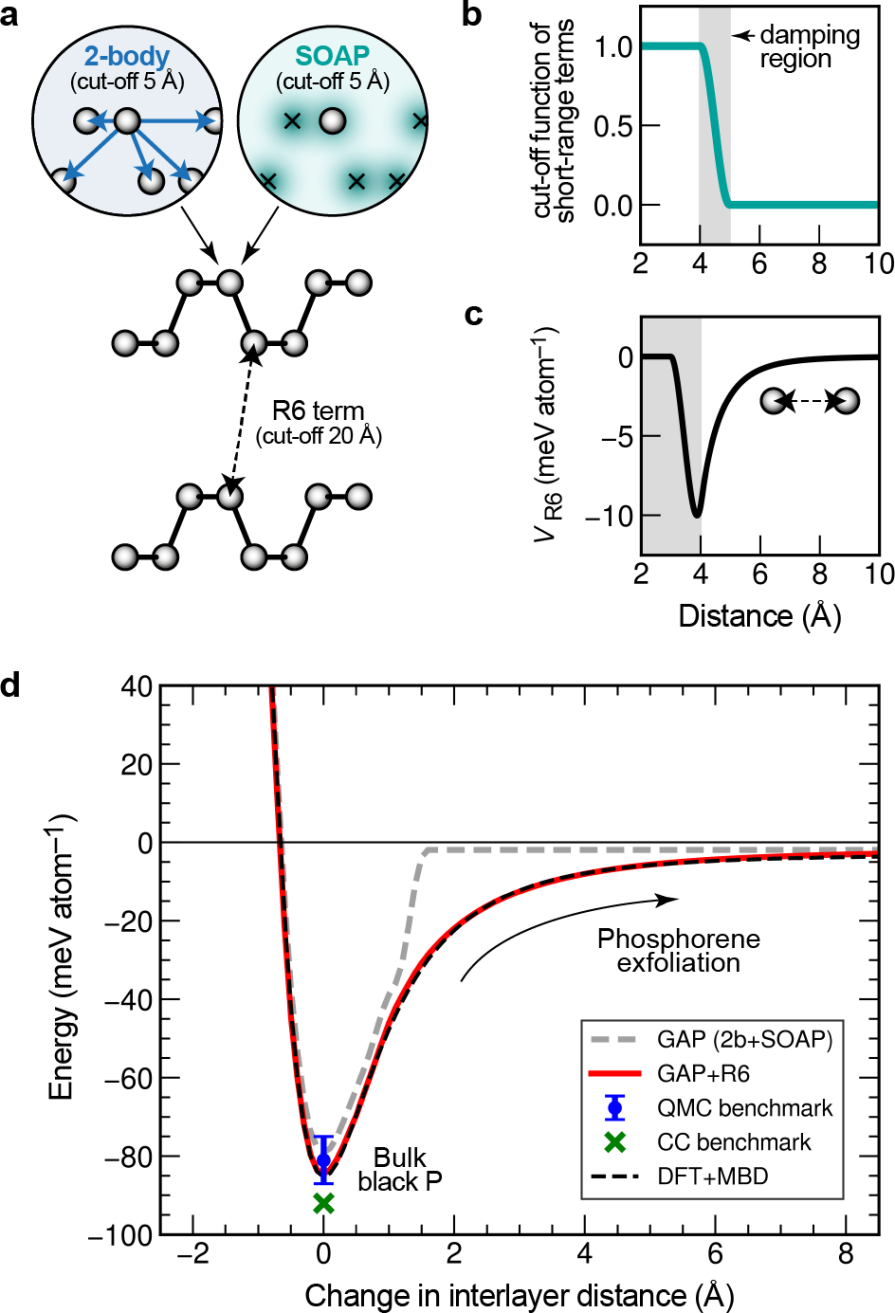
Hierarchical descriptors and fitting at the example of a general-purpose GAP for phosphorus.^163^ (a) Combination of 2-body and SOAP terms constitutes the short-range GAP model—both terms being fitted simultaneously, with appropriate scaling factors, similar to ref (122). In addition, a 1/*r*^6^ (or “R6”) term is used, with a much longer cutoff. (b) Illustration of how these terms are smoothly brought to zero in the region up to the cutoff. (c) *V*_R6_ term for longer-range interactions. (d) Phosphorene exfoliation curve, showing the performance of the combined “GAP+R6” model (red) compared to a short-range GAP (gray dashed line), the DFT+MBD reference (black dashed line), and high-level quantum chemistry benchmark data (blue and green markers). Reprinted from ref (163), where more detail and references may be found. Original figure published under the CC-BY 4.0 license (https://creativecommons.org/licenses/by/4.0/).

Figure 19 illustrates several of the concepts discussed in the context of hierarchical GAP fitting, using as example the general-purpose phosphorus potential of ref (163). Here, both aspects discussed in the preceding paragraphs are now relevant: the combined 2-body and many-body GAP fitting (which are both used to describe the atomic neighbor environments up to 5 Å) and the use of an additional, longer-range empirical baseline. The reason for the latter is the importance of van der Waals (vdW) dispersion in various phases of phosphorus: this includes interactions between P_4_ molecules, phosphorene sheets, or tubular motifs, and even an accurate energy ranking of the bulk allotropes that requires vdW effects to be included in the computational treatment.^166^ A benchmark study illustrated how the interlayer spacing and exfoliation energy in the structurally comparatively simple black phosphorus is described in very different ways by a range of computational methods, and sophisticated approaches are required to achieve even satisfactory behavior.^178^

### 4.3 Sparse GPR

All GAP models are sparse kernel models (see section 2 for a detailed exposition of the distinction between full and sparse GPR), which means that the basis functions for the linear expansion of the atomic energy do not directly correspond to the set of input data to which the model is fitted. This is rather natural for fitting a model of atomic energies, since that is not a quantum mechanically defined observable; only the total energy is. The total energy, as well as many other microscopic observables to which we wish to fit, are *linear functionals* of the atomic energy—e.g., the Hellmann–Feynman forces are derivatives of the total energy with respect to atomic positions, and therefore also sums of derivatives of the atomic energy function, and so are stresses.

For the case of a single and fixed system size, one could develop a non-sparse (full) GP model, in which the total energy that we ultimately want to predict is written using a linear combination of basis functions each of which precisely corresponds to an observed data point (irrespective of whether it is an energy or a force component), the linear algebra (as outlined in section 2, both in the kernel learning framework and the GP framework) is straightforward, and indeed the sGDML model^179−181^ does exactly this, very successfully, to obtain potential-energy surface models of specific molecules using a few thousand input data values. However, such a model is not applicable to a different sized system (even one composed of copies of exactly the same set of atoms). For most materials modeling applications, transferability to different system sizes (in fact exact size extensivity) is a fundamental requirement. Furthermore, it is empirically the case that vastly fewer (≈10^4^) basis functions than observed data values (≈10^5^) are *sufficient* for the construction of very accurate interatomic potentials for materials. Since solving the linear algebra problem of fitting sparse GP scales with the square of the number of basis functions and linearly with the number of data points, using the sparse model results in an enormous saving compared to a full GP. In the GAP framework we choose individual atomic environments as the elements of the representative set, and the corresponding kernel basis functions are used to expand the atomic energy.

Given a fixed training dataset, we consider the number of basis functions (or equivalently the size of the representative set) to be a convergence parameter. In practice, it is clear that for small basis set sizes, the accuracy of the model improves dramatically when the basis set is increased but eventually levels off: the remaining error is dominated by a combination of locality error (see below) and lack of input data diversity. As well as the total number of entries, the critical point is that the representative set needs to encompass the diversity of the training set. One could just pick the representative set randomly from the available training configurations. The disadvantage of uniform random selection is that the chosen basis set is heavily influenced by the way the training set is put together. For example, we would like it to be the case that putting more data of a particular phase or a particular type of molecule should not make the fit worse for other unrelated types of configurations. By skewing the distribution of the basis set, uniform random selection can easily result in some types of configurations to not make it into the basis set at all and thus reducing the diversity of the representative set, leading to a significantly worse model performance for the corresponding types of configurations.

To ensure diversity in the representative set, we experimented with a number of strategies. For low-dimensional descriptors, such as 2-body terms, it is sufficient to ensure that all interatomic distances (within the cutoff) are well represented, and therefore a uniform grid in the one-dimensional space of the descriptor is chosen. Such a strategy is not efficient for the high-dimensional representations such as SOAP, so here we recommend the leverage-score CUR algorithm,^144^ which maximizes the span of the basis set in a linear sense in the high singular value subspace of the full training set. Note that leverage-score CUR was designed as an alternative to PCA that guaranteed that the selected points were in fact real data points, which is not actually required for sparse GPR models. Nevertheless, we have empirically found it to be a good algorithm for use in constructing SOAP-GAP models. Whether basis functions are centered on data points or not can, in principle, have some effect on the quality of the fit (especially for derivative observations), as seen in Figure 6—but for the SOAP hyperparameters we recommend here, we do not expect that to be the case. In a loose sense, selecting representative points using CUR from a much larger set can be viewed as a cheap proxy for optimizing their location.

### 4.4 Locality

**Figure 20:**
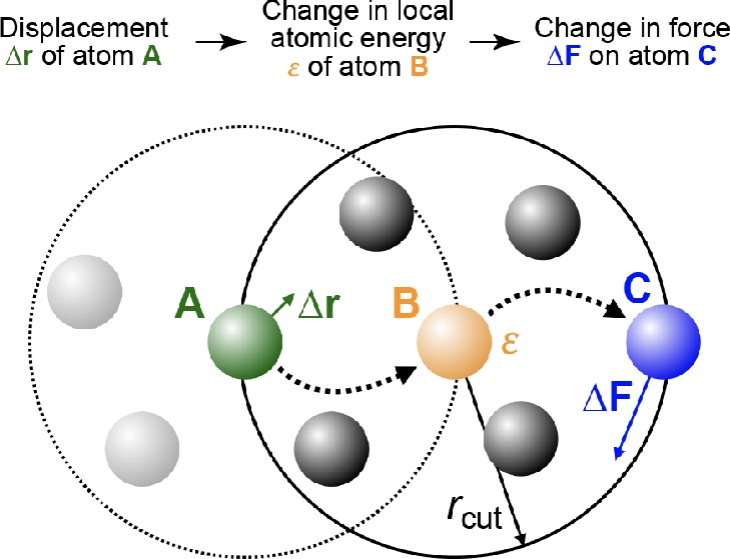
Locality of forces in case of atom-centered three- or higher-body order potential terms. The displacement of atom **A** affects the local atomic energy of atom **B**, which in turn affects the force on atom **C**. Both atoms **A** and **C** are just within the cutoff radius of atom **B**, and thus the locality of the forces in the model is *twice* the cutoff radius.

In general, atomic interactions are expected to be long-ranged, due to electrostatics, charge transfer, and dispersion. Despite this, interatomic potentials with finite cutoff radius have been successful in describing many materials, due to the effects of screening. Formally, for an interatomic potential model with three- or higher-body interaction, displacing an atom affects the force on other atoms in a range of up to twice the cutoff radius of the model, as illustrated in Figure 20.

This assumption of locality imposes an inherent limitation on the accuracy of the interatomic model: any long-range effect that would otherwise be observable from the quantum-mechanical description of an atomic system will not be captured by the model. In the context of ML potential fitting, this nonrepresentable contribution to the interactions between far-away atoms is manifested as noise, or uncertainty, in the input data because two atoms with locally identical configurations might still experience different forces. Knowing the magnitude of this uncertainty for a material is useful: it corresponds to the smallest attainable error of a potential model with a given cutoff radius, *entirely independently* of what descriptor or fitting method is used to make the potential. In other words, no finite-range potential can be more accurate, regardless of the amount of training data or degree of model complexity.

**Figure 21:**
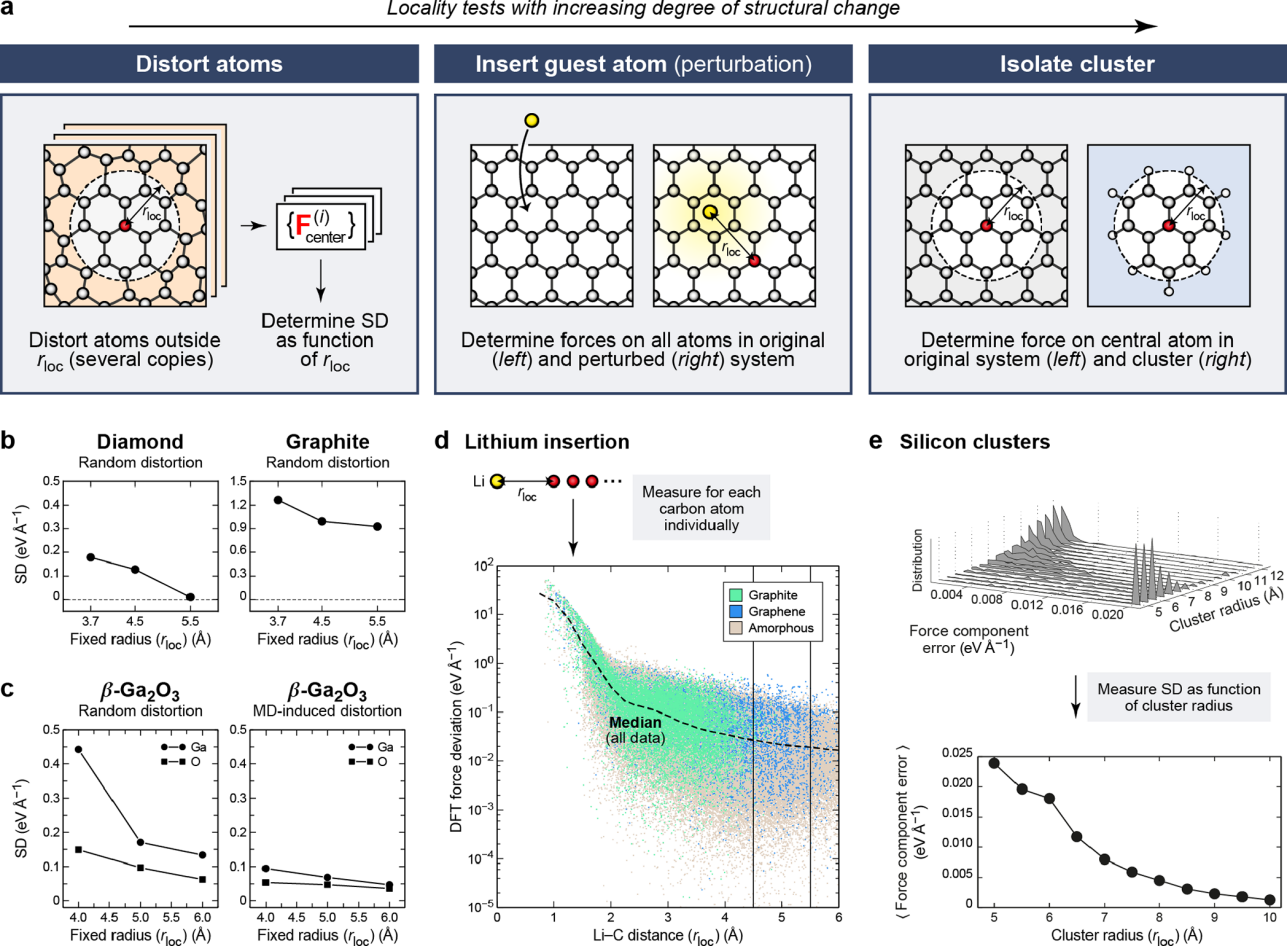
Protocols for quantifying force locality. (a) Schematics of three approaches that make increasingly drastic changes to the structure up to a characteristic radius, *r*_loc_—drawn following ref (122), with the left panel adapted from that work. From left to right: (i) distortions of atoms outside *r*_loc_ around a central atom, estimating locality by measuring the standard deviation (SD) of the force on this atom as a function of *r*_loc_; (ii) insertion of a guest atom, estimating locality by measuring the change in the forces on all atoms depending on their distance from the guest atom, taken to be *r*_loc_; (iii) isolation of a cluster fragment with radius *r*_loc_, estimating locality by determining the force difference for the central atom between the cluster and the original system. (b) Results of locality tests for diamond and graphite, highlighting qualitatively different behavior: in diamond, the interactions decay quickly, and perturbing atoms more than 5.5 Å away from the center does not substantially influence the force on the central atom. In graphite, on the other hand, there is a high degree of nonlocality. Reprinted with permission from ref (122). Copyright 2017 by the American Physical Society. (c) Same for β-Ga_2_O_3_. Two different strategies were used: random distortions, as in the panels above, or MD-induced distortions. Adapted from ref (182). Copyright 2020 AIP Publishing. (d) Force locality in graphitic and other carbon structures, where the perturbation is the addition of a Li atom. Adapted from ref (119). Copyright 2018 AIP Publishing. (e) Force locality in bulk silicon configurations, estimated via the force component differences on the respective central atom between clusters of different radii and the corresponding original structure.^183^ Republished with permission of IOP Publishing, from ref (183); permission conveyed through Copyright Clearance Center, Inc. © 2005 IOP Publishing. Reproduced with permission. All rights reserved.

One can quantify the degree of locality in a material directly using quantum-mechanical calculations. The following procedure provides an estimate of the lower bound of the force localization. Given an atomic configuration *A*, the environment *A*_*i*_ around atom *i* is fixed, and the positions of the remaining atoms in *A* are perturbed, resulting in configurations *A*′. The standard deviation of the quantum-mechanical force **F**^(*i*)^, measured on atom *i* as embedded in different configurations *A*′, provides the lower bound on the force locality. This procedure is illustrated in Figure 21a.

The magnitude of this standard deviation will, in practice, depend on the magnitude and type of perturbation of the other atoms outside the environment *A*_*i*_. The ensemble of perturbations may be motivated by the physics of the system and the configuration space intended to be studied. For example, the locality of forces in diamond and graphite were determined by applying uniformly random perturbations or MD simulations that selectively moved atoms outside the fixed radius. Figure 21b presents the measured locality of quantum-mechanical forces at the DFT-LDA level, for different radii of the fixed environments in diamond and graphite.^122^ These results show that locality can be highly structure dependent, and materials of the same composition can display a large disparity in the locality of the atomic interactions for different phases. Indeed, given the lack of significant charge transfer (and hence no long-range electrostatic interactions) in these systems, the main qualitative difference is in the nature of the electronic structure, with diamond being an insulator and graphite a semimetal.^184^

Forces due to uniformly random perturbations of a crystalline structure convey essentially the same information as the orientation-averaged force-constant matrix, but in a disordered system a different ensemble of perturbed configurations conveys more relevant information on the locality. For instance, in a liquid, a much larger configuration space is available for the atoms outside the fixed environment *A*_*i*_, which can be conveniently sampled by molecular dynamics. Figure 21c shows the results of using uniform random distortions as well as MD for the case of crystalline β-Ga_2_O_3_.^182^ The absolute magnitudes are dependent on the size of the distortions, and MD will sample the Boltzmann distribution and therefore generally shows smaller force deviations than uniform random distortions. For any given application, the ensemble of perturbations should be chosen bearing in mind what kind of distribution will be sampled once the potential is being used for making predictions.

Other than moving atoms outside the atomic environment *A*_*i*_, it is possible to perturb the configuration by adding atoms to the configuration. It is expected that the addition of an atom affects those closest, and the effect decays with increasing distance. Fujikake et al. studied the intercalation of Li in carbon structures and quantified the localization error of forces on carbon atoms due to the presence of a Li atom.^119^ Quantum-mechanical force components computed at the DFT-LDA level were compared on the same structures with and without an interstitial Li atom, respectively, and the deviations are shown in Figure 21d as the function of distance from the Li site.

A much more drastic perturbation is to isolate a finite cluster corresponding to the fixed environment *A*_*i*_ and comparing the quantum-mechanical forces obtained on atom *i* in the cluster with open boundary condition to that of the periodic reference calculation. Such a study was performed on bulk silicon with a defect.^183^ To minimize the effect of metallic states due to the surface atoms on the clusters, they were terminated by hydrogen atoms. The locality of the forces improves suddenly for a cutoff beyond 6 Å, as seen from the results in Figure 21e, suggesting that this is the length scale of electronic locality in this material.

Overall, such tests objectively inform the developer of a potential what force accuracy is achievable using a given cutoff radius. However, it is important to note that different body-order interactions may have significantly different locality properties, and these tests only present the locality in terms of fixed many-body environments, the worst-case scenario. It is often feasible to use different cutoff radii for different body-order terms as dictated by the locality of the specific interactions.

We only considered the locality in covalently bonded systems, but similar questions are worth asking about other materials. Interestingly, although electrons are highly delocalized in metals, the very short screening lengths give rise to favorable locality properties, which is evidenced by the long history of useful short-range empirical potentials and also successful GAP models (see section 6.1). When modeling liquids that are strongly ionic or polar, the traditional wisdom is that the explicit treatment of long-range electrostatic interactions is essential—nevertheless, successful potentials have been made using short-range cutoffs for water^185^ and even ionic melts such as LiCl^186^ and HfO_2_.^130^ The rising level of interest in short-range many-body ML potentials has led to a dedicated study of locality for water.^187^

Finally, the locality test described above is not only useful when assessing the limitations of a short-range model. When the long-range interactions in a material need to be included explicitly, these are often described by an analytic baseline model (see above in section 4.2). Once such a baseline model is chosen, the locality of the original target potential with baseline subtracted can be measured, since this is the difference potential that will be fitted with the short-range ML model. The logic can be reversed too: the optimal baseline model for the purposes of hierarchical ML fitting is the one which, after subtracting it from the original target, leads to the best force locality.

### 4.5 Practical Choices for Hyperparameters

Many hyperparameters are required to specify the regression problem precisely—this is a common feature of all nonparametric modeling approaches. It is common to treat these degrees of freedom by optimization. While naively it might seem that simply minimizing the fitting error on the training set is how one should proceed, this is not the case. (This is in fact the reason for the notion of *hyper* parameters as opposed to regular parameters.) The issue is that the total number of degrees of freedom is so large (in the GPR framework, the coefficients of the representative points; in neural network fits, the connection weights and biases) that there is always a danger of overfitting to the training set—yielding a model that is useless because it would give uncontrollably large errors on any *test* data that have not been included in the training set. This is most easily demonstrated for simple GPR with the Gaussian kernel: if the length scale of the Gaussian is chosen to be very small, the kernel matrix becomes diagonal. In this case, the fitted function is a sum of extremely narrow Gaussians, each with a magnitude equal to its corresponding training data point, and therefore giving nearly zero value away from any training point. See Figures 3 and 9a for two examples of this overfitting behavior.

The two most common ways to optimize hyperparameters while avoiding overfitting are cross-validation and marginal-likelihood maximization (section 2.6). While these techniques are very general and often work well, in the specific case of fitting interatomic potentials, we can usually do without them. Good values can be chosen *a priori* using physical and chemical principles and specific knowledge about the target functions. There are several advantages to doing this, beyond the obvious one of saving computational effort. First, our choices are not contingent on having a sufficiently large training set or a sufficiently diverse test set, which are needed for the general methods to work effectively. Second, the above methods only make sense when the complete dataset with which we work is fixed (prior to splitting it into training and test sets). But in our case, this is not so: we can and should consider the composition of the dataset to be open to optimization too! So the problem is turned upside down: instead of finding the best hyperparameters for our training set, we choose the hyperparameters that express our prior knowledge on the nature of the function we are fitting, together with a target accuracy (which is intimately related to some of the hyperparameters, see below), and then build our dataset in such a way that our accuracy goal is achieved.

In the context of GAP, we distinguish two classes of hyperparameters. On the one hand, there are those of the kernel itself, whose choice is driven by the underlying physical modeling assumptions such as the cutoff radius and the basis truncation coupled with the length scale of the mollification of the neighbor density that together control the smoothness of the kernel. On the other hand, other hyperparameters have more to do with the composition and nature of the dataset itself, such as the selection of representative atomic environments that correspond to the basis functions in the sparse kernel regression model and the regularization parameters that act like weights on the different parts of the dataset.

#### 4.5.1 Cutoff Radius

We discuss the kernel hyperparameters first. The most important parameter, which appears in every short-range interatomic potential, is the radial cutoff distance. This applies not only to interatomic potentials but to any atomistic model that is describing how a property of an atom depends on its neighborhood, e.g., a model of NMR chemical shifts or atomic polarizability. It does not however apply to models that are not explicitly range restricted, e.g., models of the intramolecular energy of isolated molecules or clusters that are built based on a representation of the entire system. Examples are the PIP models of Bowman and Braams^63^ and Paesani,^188^ many other expansions of molecular potential-energy surfaces (see references in section 5.4), and also the GDML models of Chmiela et al.^179^

Every finite-range potential can be cast in the form of a sum over *site energies* or *atomic energies*, and the cutoff radius defines the range of this local term. The actual interaction range is twice the cutoff radius, because atoms up to this distance can potentially interact with one another via a many-body term centered on an atom in between them (Figure 20). As detailed in section 4.4, when we approximate a quantum-mechanical potential energy (which is not formally local) using a local atomic energy with cutoff radius *r*_cut_, the error we necessarily incur can be characterized in the form of a force variance. In section 4.4, we had therefore described direct tests to measure the *possible* accuracy of a local model^122,183^—irrespective of the representation, regression, or other aspects of the model. We propose to use the measured force variance, which we call the “locality error” for a given cutoff, as a benchmark against which the ML model (or indeed any model with that cutoff) should be tested. Once this accuracy has been reached, the model can be considered fully trained, and the only way to make it better is to increase the cutoff radius.

In practice, this concept of the locality error is often used in reverse. We set a target prediction accuracy before the model is created (e.g., we wish to achieve 0.1 eV/Å accuracy on the force components) and determine the required cutoff distance that results in a locality error below our target. We are not aware of successful fits with cutoffs much beyond 6–8 Å with descriptors that have full atomic resolution and aim to retain all geometric information. Thus, if the locality error suggests that larger cutoffs are necessary, then either the accuracy target needs to be revised or multiple hierarchical models need to be used that, with some range separation, describe long- and short-range interactions (section 4.2).

#### 4.5.2 Kernel Regularity

Part of the success of kernel fitting can be attributed to the fact that well-chosen kernels impose regularity on the model, complementing the usual regularization practice (which will be discussed in section 4.6). Having fixed the cutoff, and therefore the local atomic neighborhood that constitutes the input to the potential, the next set of hyperparameters to think about are the ones defining the spatial resolution or, equivalently, the *regularity* (smoothness) of the representation. For two-body and three-body kernels, this might be the spatial length scale of the basis functions (e.g., Gaussians) that are used to expand the model or, in the case of the SOAP representation, the length scale of the Gaussian that is used to mollify the neighbor density, *σ*_*a*_. A larger length scale will smear the density more and result in a smoother potential (for a fixed number of representative points in the GP) but at the cost of reduced accuracy, perhaps compensated by reduced overfitting.^140^ In practice, for fitting interatomic potentials to quantum-mechanical potential-energy data, the appropriate length scale is about *σ*_*a*_ = 0.3 Å in the presence of hydrogen atoms and *σ*_*a*_ = 0.5 Å for atoms up to the third row in the Periodic Table (with no hydrogen present). Larger length scales could be used when fitting potentials for structures solely containing elements with large atomic radii. A larger SOAP-kernel length scale—that is, a smoother description of the structure—was found to be important in the initial work on GAP-RSS: boron, despite its small atomic radius, was described with a setting of *σ*_*a*_ = 0.75 Å, enabling iterative exploration of the potential-energy surface from randomized configurations only (section 4.1.3).^140^

SOAP uses an expansion of the neighbor density in spherical harmonics and a radial basis, and once the density has been mollified, it makes sense to truncate this expansion, which is achieved using the band limits *n*_max_ in the radial and *l*_max_ for the angular part. In contrast to the density mollification length scale, these band limits are not true hyperparameters, but *convergence parameters*, because higher band limits will always result in a more accurate representation of the mollified density.

**Figure 22:**
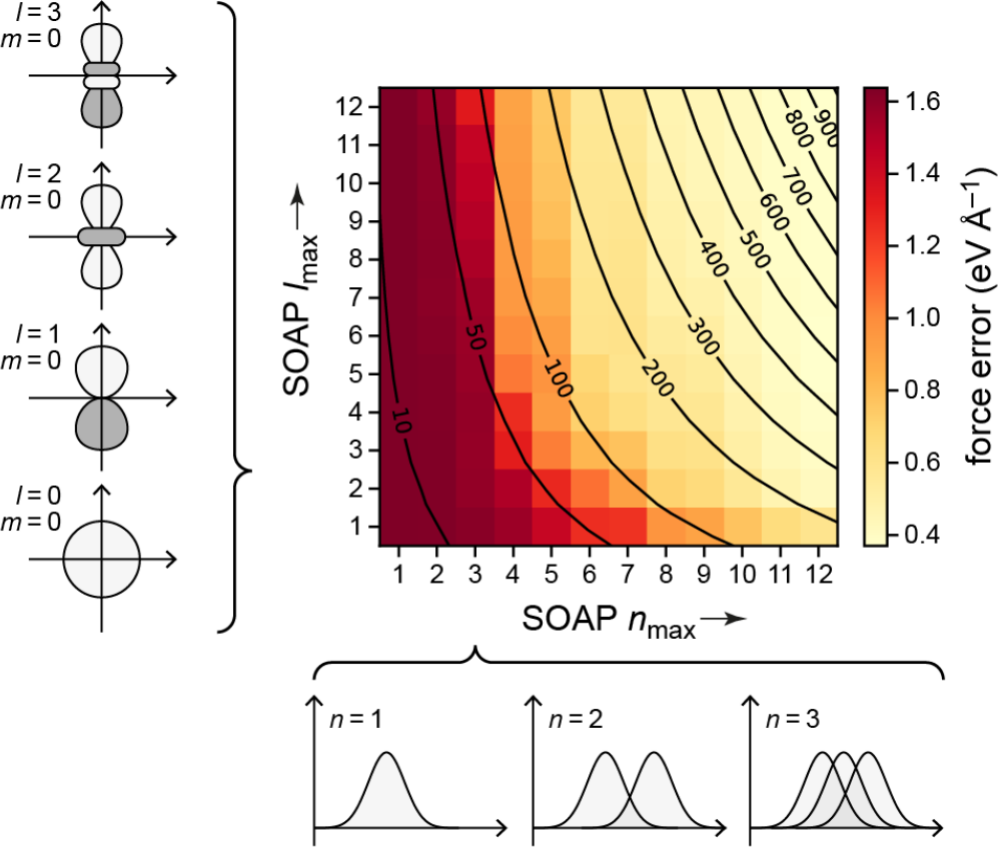
Performance of a SOAP-GAP fit to a database of carbon configurations, as a function of the number of maximum radial (*n*) and angular (*l*) components in the SOAP kernel.^152^ The color map indicates the force error. Here, the black contour lines approximately indicate parameter choices resulting in equal length SOAP descriptor vectors, corresponding to roughly equal computational cost of prediction. Schematic drawings illustrate the role of the different functions that are affected by the *l*_max_ (angular functions) and *n*_max_ (radial functions) convergence parameters, respectively; the equidistant Gaussians that are shown in the lower part of the figure are subsequently orthogonalized in the construction of the SOAP kernel.^52^ Drawn with data from ref (152).

The convergence of the accuracy of potentials in terms of the band limits for a fixed cutoff and mollification length scale is shown in Figure 22. The important result is not so much the absolute value but the *relative* accuracy. While many early GAP fits used equal values for *n*_max_ and *l*_max_ for simplicity, e.g., (*n*_max_, *l*_max_) = (8, 8) for C-GAP-17,^122^ the figure shows that while giving reasonable force errors, this choice is clearly not optimal: higher radial band limits (*n*_max_ > *l*_max_) give better accuracy at the same total cost. In this case, for example, a lower error would be expected for (*n*_max_, *l*_max_) = (12, 3)—note that the contour lines in Figure 22 provide an estimate for the computational cost of the prediction. Generally, a setting of (6, 2) would correspond to a low accuracy potential with a short descriptor vector, while (12, 6) would lead to a very accurate potential. These numerical results, shown here for the implementation of SOAP in the GAP code, depend strongly on the particular choice of radial basis functions and might well be different in other implementations of SOAP, such as in Dscribe,^189^ librascal,^95,96^ soap++,^190^ TurboSOAP,^191^ and the implementation in VASP.^123^

### 4.6 Regularization in GAPs

The regularization of the linear expansion coefficients is a key part of successful kernel ML models. Purely in the linear algebra context, it is simply considered a trick to help with the ill-conditioning arising from the near-linear dependence of the basis functions; this does not offer any guidance on what the size of the regularization term should be. In the formally equivalent GPR view, the same role is played by the hyperparameters corresponding to the variance of the stochastic noise that we assume to be present in the input data. This view suggests that if we use a Tikhonov regularizer (eq 5 in section 2.1) of a given value, we are assuming noise in the input data of about the same size, and we should not expect an accuracy better than this level. Indeed, if our model *appears* to be more accurate, that is an almost sure indicator of overfitting, an inadequate test set, or some other shortcoming of the procedure. Therefore, if we can estimate the actual level of noise in our data, the theory of GPR suggests using that value as the regularization hyperparameter—and to add data to the model until the corresponding level of accuracy is reached.

#### 4.6.1 Noise in the Input

Is there noise in the electronic-structure calculations that describe potential-energy surfaces? The answer is subtle and somewhat surprising. Once the parameters of a quantum chemistry or plane-wave DFT calculation are specified, including perhaps the pseudorandom number that initializes the computation, one might consider the ground-state energy and its derivatives (forces, stresses) to be deterministic functions of the inputs, and therefore free of noise. There are three reasons why this is a misleading view in the context of fitting interatomic potentials.

The first reason is the locality error to which we have alluded above: our model uses a finite cutoff to describe atomic properties, assuming “near-sightedness” and sufficient screening, and the extent to which this is not accurate is an indeterminacy of the target function (the atomic energy) in terms of its inputs (i.e., the local environment). In the case of GAPs, and in fact any interatomic potential with a finite cutoff, the reference (typically DFT-computed) force on an atom is not exactly determined by the positions of the neighboring atoms within the cutoff, and so when it is modeled as such, it appears to have some component of noise.

The second reason is to do with inconsistency between different pieces of data. Typically we fit potentials to both energies and forces, and the extent to which the calculated forces are the true derivatives of the energy (or indeed, if we do not explicitly fit to energies, then the extent to which the forces are curl-less, i.e. the directional derivative of a well-defined scalar function) hinges on numerical approximations. The details depend on the particular electronic-structure method and can often be adjusted by choosing convergence parameters. The level of this noise, understood as the difference between the observed data and the true values that would correspond to perfect data consistency, can be measured by numerical experiments. We find that input data for fitting potentials must be significantly more stringently converged than what is typically used for direct studies of electronic structure, because the latter often benefit from error cancellations.

One aspect of this convergence requirement, namely *k*-point sampling in periodic calculations, requires special attention, because the corresponding errors are often underestimated. As the cell parameters and lattice vectors are varied (as is typically the case in databases for materials; section 4.1), the *k*-points at which the Brillouin zone is sampled also move around, and two slightly different simulation cells might end up having dissimilar *k*-point grids. The resulting data inconsistency is of the same order of magnitude as the overall convergence error of the finite *k*-point grid; it depends on the particular scheme to generate the grid and on the symmetry and shape of the cell. Morgan et al. recently characterized this error^192^ and found that a linear *k*-point spacing of 0.1 Å^–1^ is needed to reliably converge the error below 1 meV per atom; this corresponds to about 1000 irreducible *k*-points per Å^–3^. (These are spacing and density units of VASP and may be divided by 2π to obtain the corresponding values for Castep.) Such high *k*-point densities are rarely affordable, especially when larger unit cells are involved. Using a variety of different cell sizes and therefore different *k* grids is often required in practice. The resulting inconsistency appears as noise from the model’s point of view, since the exact same local environment, when part of different periodic unit cells with different *k*-point grids, will appear to have different energies and forces. Even with highly converged grids, depending on the system, the corresponding error may exceed that due to locality and therefore should inform the choice of regularization.

#### 4.6.2 Dealing with Inhomogeneous Data

All the above considerations help to quantify the lowest achievable error and can therefore be used to set the *minimum values* of the regularizers for energy, force, and virial stress data. But the actual values we set might very well be larger. Apart from the simplest cases, the datasets to which we fit are not homogeneous: they include samples from multiple phases (say, liquid and solid) and may in fact range from nearly random (e.g., in GAP-RSS; section 4.1) to further relaxed configurations that are much closer to low-enthalpy crystalline structures. It is not practical, or indeed desirable, for our potential to aim to have the same accuracy for all these disparate configurations. This is because we care about accuracy for *properties* more than the pointwise accuracy of the potential energy for each configuration (the rather intricate question of what makes the whole GAP model “accurate” will be discussed in the following section). The elements of the regularizer, **Σ**, control how closely the fitted potential is constrained by the corresponding data. Again, consider the GPR view of the regularizer: all else being the same, a larger regularizer corresponds to assuming a larger observation noise variance, and hence it loosens the fit to that data item.

The relationship between the accuracy of the fit to the PES for a group of configurations and the accuracy of observables that depend “mostly” on those configurations is complicated (and largely unexplored, both theoretically and computationally). Nevertheless, it is easy to make qualitative statements. For example, we would like to have lower absolute error for solid configurations (close to local minima of the potential) than for liquid configurations, where the interest is in radial and angular distributions or diffusivities, which are statistical properties that are empirically observed to be well converged already while the pointwise errors on energies and forces remain larger. We express such empirical knowledge by setting larger regularizers for groups of data expected to tolerate larger errors without compromising the accuracy of observables. In turn, this will allow the fit to use its flexibility to achieve lower error for configurations where that is needed. Typical values that have worked well are (σ_E_ = 0.001, σ_F_ = 0.05, σ_V_ = 0.05) for a crystal and (σ_E_ = 0.03, σ_F_ = 0.2, σ_V_ = 0.2) for a liquid configuration, with units of eV/atom for energies (σ_E_) and virial stresses (σ_V_) and eV/Å for force components (σ_F_). A loose approximate heuristic for solid configurations with well-defined local minima (valid when using units of eV and Å) is that the target accuracy on energies (which scale with the square of the displacement) is the square of the target accuracy on force and virial stress components (which scale linearly with displacement).

The above argument underscores why the regularizers of GAPs that are fit to diverse datasets are not set using conventional cross-validation procedures by measuring the RMSE on a small test set: the *actual* errors that we wish to minimize are very costly to evaluate, perhaps requiring large-scale MD. In principle, it might be possible to set up an automated procedure that computes the complex observables and adjusts the regularizers accordingly, thereby improving the model further. In the absence of such a procedure, we have found that simple heuristics work effectively and produce very accurate potentials. Examples (with the quality measure being, say, the accurate description of an amorphous structure which can be validated against experimental observables^193^) will be given in one of the following sections.

#### 4.6.3 Implementation

Once the appropriate regularizers are chosen for each energy (E), force (F), and virial stress (V) data item in each group of configurations in the reference database, their values, σ^2^, are collected into the diagonal matrix **Σ** that scales the Tikhonov regularization term (eq 5),
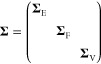
where **Σ**_E_, **Σ**_F_, and **Σ**_V_ are themselves diagonal matrices and correspond to total energy, force, and virial data components. For simplicity of presentation, we assumed here that the data items are sorted in such a way that all total energies come first, then all forces, and finally all virial stresses. The matrix corresponding to *N* total-energy data points is then
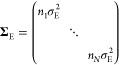
where *n*_*i*_ refers to the number of atoms in each of the *N* structures, which are not necessarily all of the same size; that is, we scale the energy (and similarly virial stress) terms by *n*_*i*_. To understand this scaling, recall that these regularization terms represent the expected deviation of our fitted function from the data due to all of the effects discussed previously. The total energy and total virial stress are extensive quantities, so all else being equal, they will scale linearly with system size, i.e. the number of atoms, *n*. If all atomic environments in a structure were the same, the variance of the total energy (which in this case would be just *n* times the atomic energy) would be *n*^2^ times the variance of the atomic energy. We scale the regularizer by *n* and not *n*^2^ because most of our configurations are far from equilibrium, so each atom that contributes to the energy and virial stress has a different local environment, and we expect some error cancellation when their contributions are added up. A more precise implementation could indicate whether the individual atomic environments in an input structure are expected to be correlated or not and adjust the scaling with system size accordingly to either *n*^2^ or *n*, respectively. This could even be determined automatically by considering the diversity of descriptor values in each configuration.

**Figure 23:**
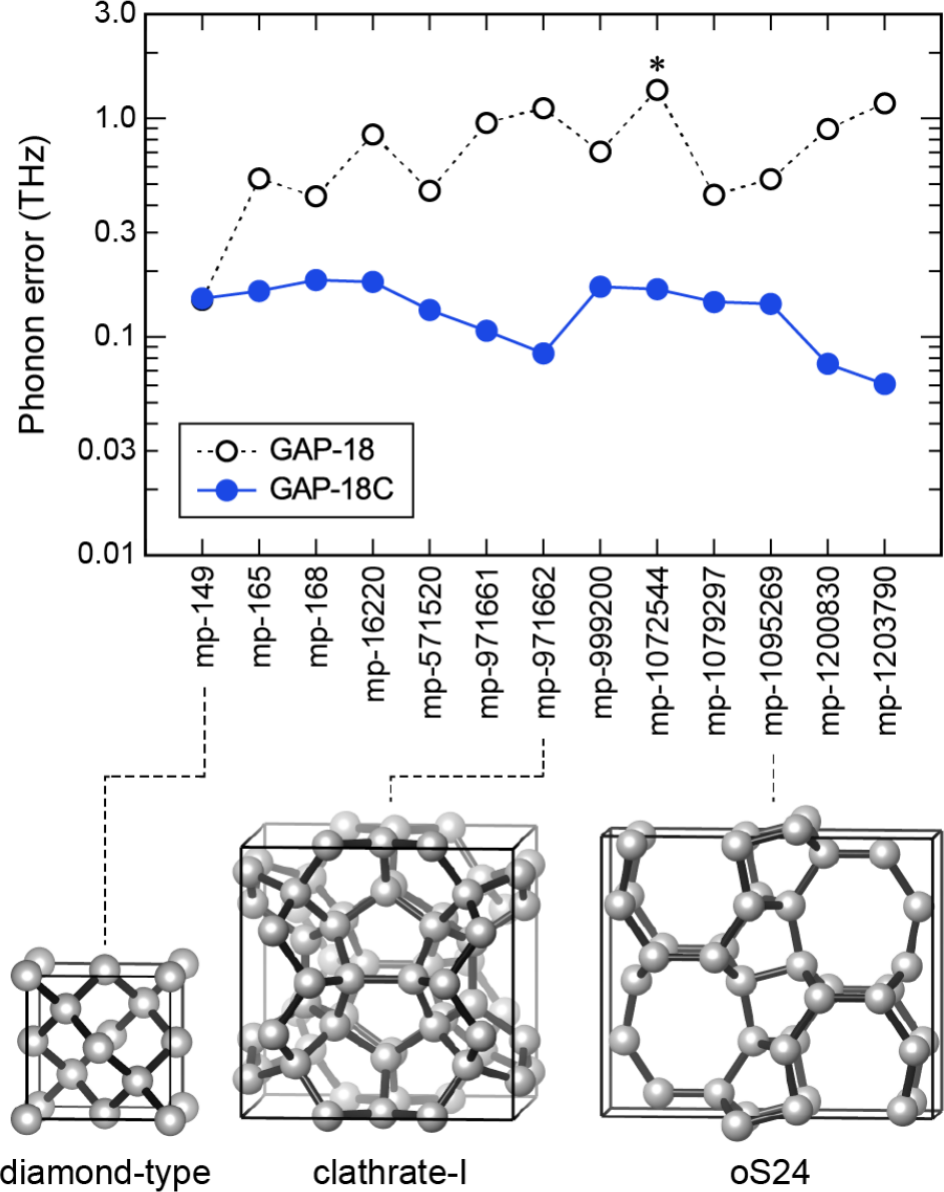
Atom-wise force regularization leads to high accuracy for phonon computations in silicon.^194^ The general-purpose GAP-18 model already predicts accurate phonon frequencies for diamond-type silicon (“mp-149” identifier in the Materials Project database;^195^ < 0.2 THz RMS error) but performs substantially worse for other allotropes, because it has not been fitted for those. The asterisk (*) indicates a structure which is erroneously predicted to be dynamically unstable. The extended GAP-18C model, which added specifically selected crystalline configurations, including supercells describing individual displacements with atom-wise force regularization, shows accurate phonon predictions throughout. Adapted from ref (194). Original work published under the CC BY 4.0 license (https://creativecommons.org/licenses/by/4.0/).

We can go further and have even finer control over the fitting weights. Rather than grouping configurations together depending on how they were generated or what structure they represent, we can set the regularization of each force component datum on each atom proportional to the size of the force on that atom. The result is a small regularization value (and corresponding large weight in the solution of the linear least-squares system) for atoms with small forces on them and a loose regularization (small weight) on atoms with large forces. This idea has been used in ref (196) and explored in a systematic fashion in ref (194). In the latter work, the aim was to extend an existing general-purpose potential introduced in ref (139) and denoted “GAP-18” for accurate phonon predictions, which is done by adding supercell configurations with small random displacements of atoms out of equilibrium.^194^ Such reference structures correspond to what would normally be used for finite-displacement phonon computations with DFT, and in fact the structures were generated using the widely used phonopy code.^197^ For this part of the extended database, we set the regularization for the force components on the *i*^th^ atom, σ_F_^(*i*)^, according to^194^
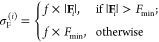
76Using numerical experiments, we found *f* = 0.01 and *F*_min_ = 0.01 eV/Å to give good results; the regularization for these “small displacement” configurations is therefore much smaller than what has been used in typical potentials that use a single value for the force regularizer. This approach was shown to lead to potentials which can very accurately predict phonons in a wide range of silicon allotropes, with an RMS phonon frequency error of about 0.1–0.2 THz for the different structures (Figure 23). It was also demonstrated that too small a value (*f* = 0.001) leads to unstable potentials, as tested by a diagnostic MD simulation: this is an example of “overfitting”, because the potential now has been made to very accurately reproduce the forces in the reference data but exhibits uncontrolled errors for other configurations.^194^ Further studies of the role of such atom-wise regularization in GAP fitting are expected to be worthwhile.

## 5 Validation and Accuracy

Once an interatomic potential model has been fitted, it must be validated before it can be broadly applied. This is particularly important in the case of ML potentials where there is no inherent physically motivated functional form. As a consequence, validation is a critical and highly nontrivial part of atomistic ML model development, particularly so for the case of ML interatomic potentials. The items reviewed here are rather specific to GAP models, but many ideas are expected to be applicable more generally.

### 5.1 Physical Behavior versus Numerical Errors

There are two related but distinct issues when evaluating the accuracy of GAPs (or in fact any ML-based interatomic potential). The more obvious one is that of goodness of fit, including numerical prediction error on the available test data. However, in practice the more serious concern is whether an MD or Monte Carlo simulation *using the potential* generates the correct probability distribution over configuration space—and therefore, whether they lead to a physically and chemically correct result. The highly flexible form of a data-driven potential means that in such a simulation many of the explored configurations are inevitably in the extrapolative regime. While prior assumptions such as smoothness help, they are not sufficient to fully control the behavior of the model outside the region represented by the fitting data, and energy errors of either sign can occur, leading to incorrect over- or undersampling in a simulation of thermal equilibrium.

It is neither practical nor necessary to achieve a uniform data coverage either in the training or the test dataset. This is only partially due to the high dimensionality of atomic configurational space, which would require extremely large quantities of data to place data points uniformly. The other reason for data sparsity is the fact that large regions of configurational space might not be *relevant*, if the corresponding potential energy is so high that an equilibrium simulation at reasonable temperature will not visit them with appreciable probability. For example, for a configuration in which at least two atoms are very close to one another, the energy is dominated by repulsion due to the Pauli exclusion principle. While such a configuration is not relevant, and therefore one might conclude that the accuracy of the model is not important here, this is not entirely true. If the prediction of the potential energy is unphysically *too low*, a simulation using the potential with such a “hole” *will* visit this region, leading to unphysical configurations with very small interatomic distances. In a simulation with the potential, the likelihood of the system finding such unrealistic regions (if they exist) monotonically increases with the length of the simulation.

If the configurations for the fitting database are generated by sampling the target potential energy (e.g., finite-temperature ab initio MD), which is naturally biased away from such configurations, it will be hard to generate sufficient data to avoid having holes in the potential. The effect of inadequate data coverage of repulsive configurations in the training set can be mitigated by adding a *baseline* model to the ML potential,^139^ as described in section 4. Such a baseline potential can be very short-ranged, serving only the purpose of imposing a sufficient repulsive interaction to prevent the system from exploring unphysically low interatomic distances.

A similar sampling problem leading to errors of the opposite type can also occur. The method used to generate fitting configurations can fail to explore important basins in the PES, for example due to energy barriers with a low transition probability in a finite simulation. This can happen if the simulation generating the fitting data is very short or if it uses a potential that overestimates the barrier (or even qualitatively fails to reproduce the existence of the missed local minimum). Since those regions would not be represented in the fitting database, the model may predict erroneously too high energies. Potential-energy errors of this type would lead GAP-driven simulations to also fail to sample the same regions, even during much longer simulations than those used to generate the fitting data.

**Figure 24:**
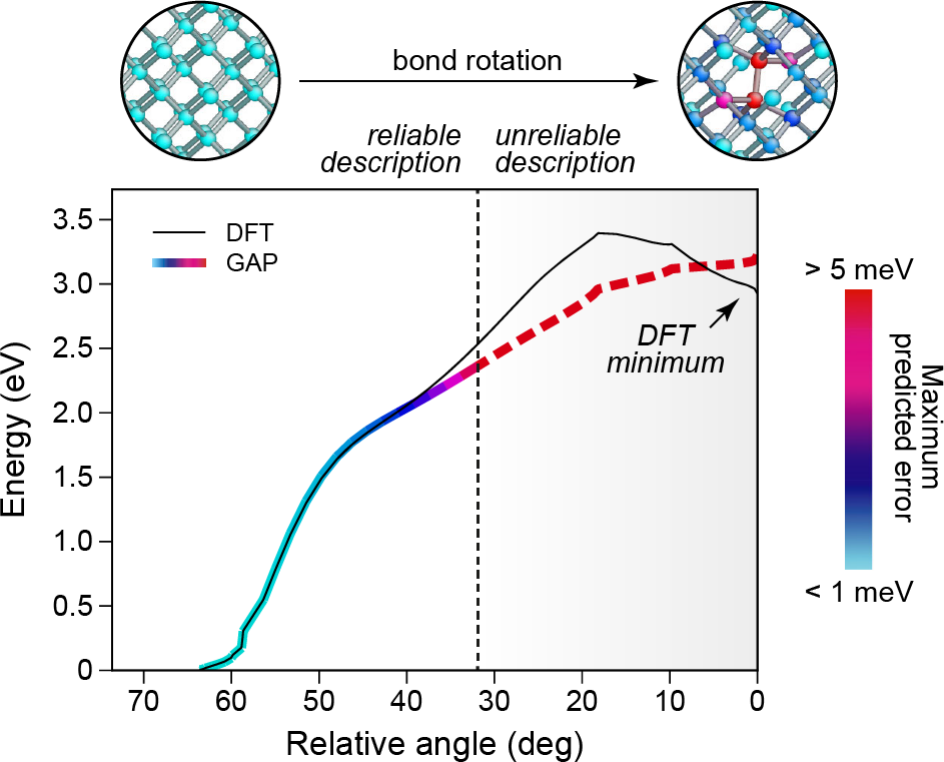
Predicted errors of a GAP model. The figure shows the energetics of the pathway leading from perfect diamond-type silicon (left) to the formation of a 4-fold defect (right); the color of the curve corresponds to the largest predicted atomic energy error in the simulation cell, given by the Bayesian error estimate. For small distortions, the GAP prediction is in practically quantitative agreement with a DFT reference (show as thin black line); for larger distortions, roughly to the right of the dashed vertical line, the prediction deviates from the DFT result, and concomitantly the predicted error rises notably. Note that DFT predicts the 4-fold defect as a local minimum (highlighted by an arrow), whereas the GAP does not. Adapted from ref (139), where details, as well as other example cases, may be found. Original figure published under the CC BY 4.0 license (https://creativecommons.org/licenses/by/4.0/).

Figure 24 shows a situation in which the GAP model is accurate to within 10 meV/atom but fails to capture an important subtlety of the DFT potential-energy surface. If the practitioner was unaware of the existence of the local minimum corresponding to the 4-fold defect in diamond-type structure silicon, its existence will not be revealed by simulations using this specific GAP model.

Exacerbating the problems of both falsely identified and falsely missed minima is the common practice of using a single dataset, generated by sampling a particular region of configuration space using a particular method, that is then partitioned into training and testing sets randomly, which therefore represent the same single region and correspondingly fail to include configurations from regions not represented by the original dataset. Achieving a low error on such a test set appears to indicate that the quality of the model is sufficient, but its transferability can be poor. As a result, rather than merely inspecting energy and force errors, a more reliable way to assess transferability of ML potentials is by performing extensive and wide-ranging explorations of atomistic configurations, such as random structure searches,^139^ MD simulations at high temperatures,^122^ or transition path calculations.^198^

**Figure 25:**
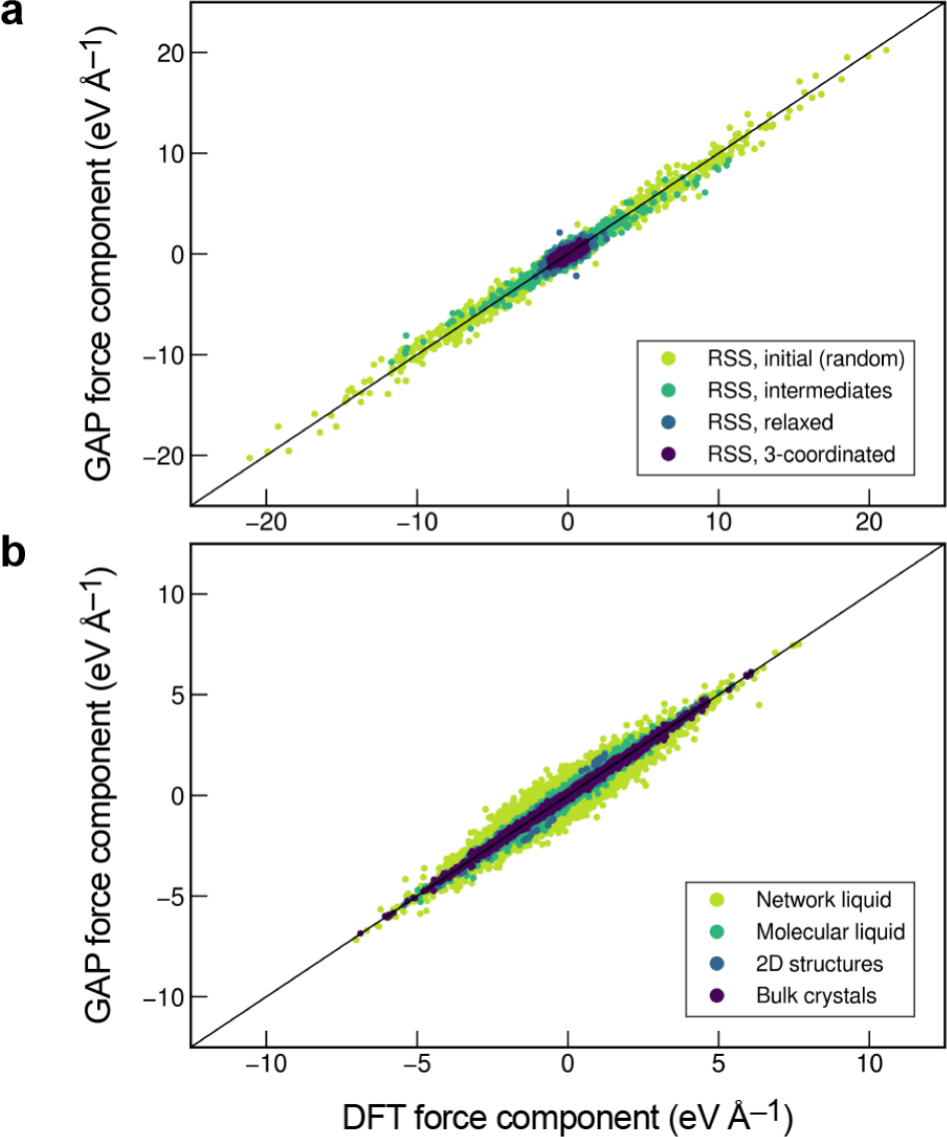
Force errors for the general-purpose phosphorus GAP of ref (163). Measuring the error in different types of configurations illustrates two aspects: the different spread of data for randomized and progressively relaxed configurations in random structure search (RSS) (panel a) and larger error for liquid than for crystalline configurations (panel b), associated presumably with a larger structural diversity in the liquid. Adapted from ref (163). Original figure published under the CC BY 4.0 license (https://creativecommons.org/licenses/by/4.0/).

Even the apparently more straightforward question of prediction error on available data is, in fact, also affected by sampling issues. In line with the standard procedures of broader ML research and applications, the most basic validation test of machine-learned potentials is the comparison of directly predicted properties, such as total energies and forces, to those obtained from *ab initio* calculations, on a test set of configurations not used in the fit. However, benchmark results such as the RMSE depend on the circumstances of the sampling from which the testing configurations were obtained. Tamura et al.^199^ computed the MAE of the force errors of ML potentials of Si and Ge on configurations sampled at different temperatures. As different parts of the configuration space are sampled, the variation of the absolute values of the forces is significantly different at different temperatures, and as a consequence the magnitude of the MAE increases with the temperature. A conceptually similar result is shown in Figure 25, where tests have been done separately for various types of configurations of very different structural nature, ranging from highly random (GAP-RSS) configurations to snapshots of phosphorene and bulk crystalline allotropes, all covered by a general-purpose GAP for phosphorus (cf. Figure 16).^163^ The different aims of the potential are reflected in the qualitatively different distributions in panels a and b of Figure 25. In the former case, the GAP-RSS snapshots serve to construct a flexible model, at the cost of a substantial residual numerical error, even for the further relaxed structural snapshots (purple). In the latter case, structures have been added by hand, and the overall magnitude of the force-component errors is about half of that in panel a. That said, the range of absolute force components is substantial for the crystalline phases as well, because distorted copies of the respective unit cells at various volumes have been included; the *errors* show a clearer trend with the degree of structural complexity, and both are largest in the network liquid (with highly diverse bonding environments) and smallest for the crystalline phases. Studying such variation is a necessary but not a sufficient test for the validity of any new ML potential, as discussed above.

Based on the preceding discussion, we argue that the validation of GAP and similar ML potential models needs to go beyond simple out-of-sample testing, and protocols should involve testing on “self-consistently” generated configurations, i.e. sampled using the potential itself. In practice, the potential can then be improved iteratively, by adding newly generated configurations using the current version of the potential to define a new potential, until it is accurate on the samples generated by itself. This design helps to eliminate “false positive” regions (overly stable or fictitious local minima), but “false negative” regions, or missed minima, are even more challenging to detect. Various approaches to using iterative fitting to build up the reference database are discussed in section 4.

### 5.2 Predicted Errors in GPR

Gaussian process regression is a statistical learning technique which generates an ensemble of functions, based on *a priori* assumptions. The prior distribution of these functions is modified by the reference dataset, resulting in a posterior distribution of functions. The mean of these functions is the GPR prediction, but it is also straightforward to compute the variance, providing an error estimate in addition to the function value. As introduced in section 2, the posterior distribution of the prediction, given a dataset , is
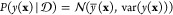
77where the mean and the variance are obtained from the analytical expressions
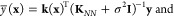
78
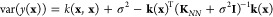
79where we have shown for emphasis the explicit dependence on the predicted values on the location **x**. Note that while the predicted mean can be calculated in time and memory that scales as the number of data points, the computational cost of the predicted variance scales as the square of this number. Using sparse GP will reduce this scaling, and analogous expressions for the predicted variance are derived in ref (43). In practice, we often use eq 79 but with the kernel matrix evaluated only on the representative set, **K**_*MM*_. The expression for the variance in eq 79 does not explicitly depend on the observations, only on the set of data locations, but it does depend on the hyperparameters (σ and also those in the kernel function). If the hyperparameters are optimized based on observations, then that brings an implicit dependence of the predicted variance on the observations.

GAP models for materials based the SOAP representation that we presented earlier essentially inevitably require the use of a sparse GP and have not been shown, in general, to lead to a quantitative prediction of the energy error. Nevertheless, for well-converged models such as the general-purpose silicon GAP in ref (139), the predicted variance was a good indicator of large actual errors. As the example in Figure 24 shows, configurations near the peak of the atom exchange pathway have large predicted error since they were not represented in the fitting database, and the corresponding actual error is also (relatively) large. In that paper, similar results were also shown for generalized stacking faults, vacancy migration, and brittle-crack tip configurations.^139^ Recently, the predicted error has been used as a tool for assessing the quality of the prediction for various regions in a large and realistic amorphous carbon film deposition simulation:^200^ it was shown that the surface regions, while being structurally highly disordered, are described by C-GAP-17 with low predicted error, because small-scale structures that are representative of disordered surfaces had been included in the construction of the reference database.^122^

**Figure 26:**
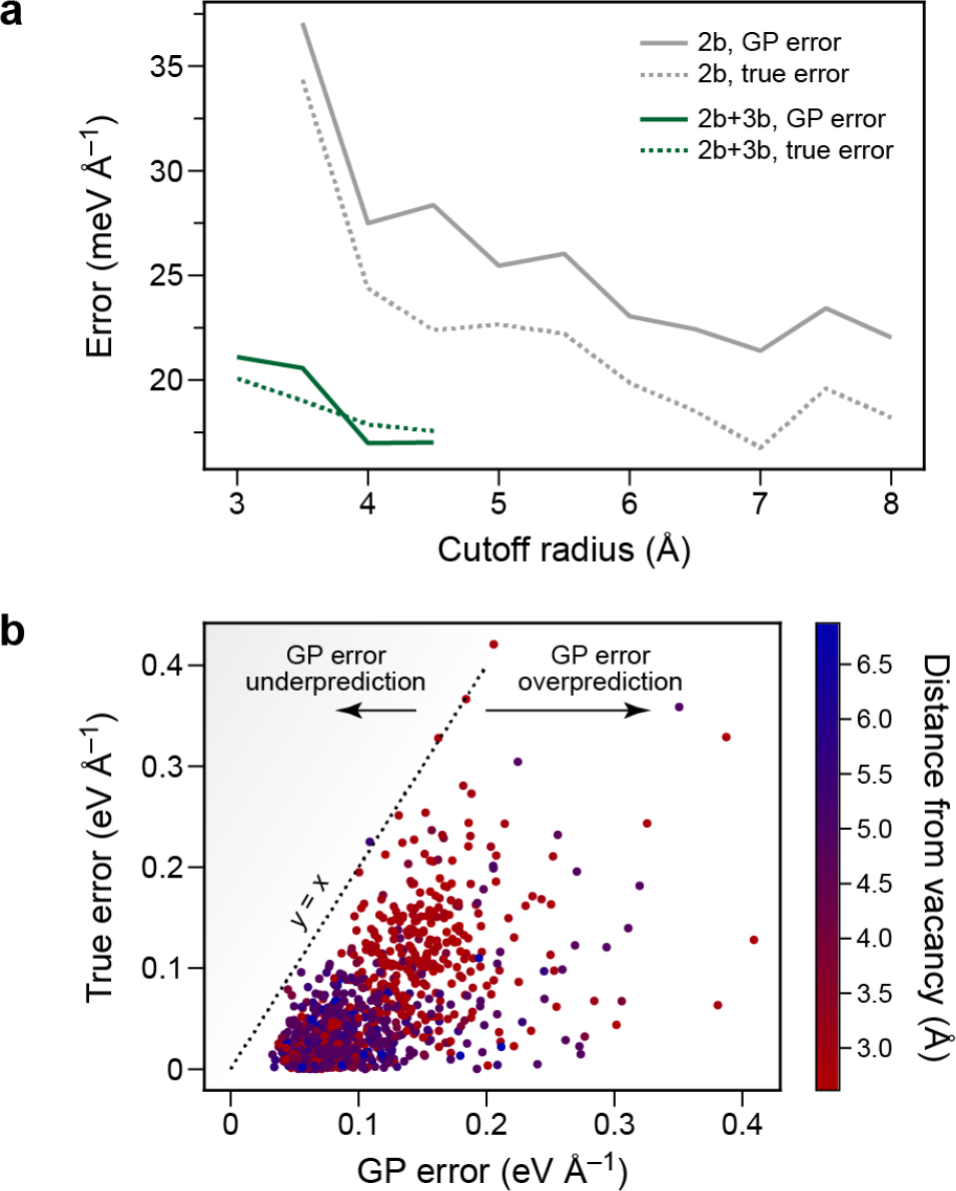
Relationship between true and predicted errors for low-dimensional GPR models. (a) GP model errors for bulk fcc aluminum for a 2-body (“2b”) and a combined 2b+3b model, as a function of the cutoff radius of the model. (b) True versus predicted model error (for a 2b+3b model) for atomic configurations near a vacancy, with the distance of each individual atom from the vacancy indicated by color. Drawn with data from ref (61).

Some work on GP-based potentials used error predictions in quantitative ways. As already discussed in section 4.1.2, a recent study in ref (61) showed good agreement between predicted and actual force errors and this was used for active learning. In this case, the number of descriptors was small, and consequently the model needed only a small number of fitting configurations which enabled the use of full GPR. Figure 26a shows the results for GPR models with low-body-order descriptors for bulk aluminum, emphasizing how the true errors (dashed lines) and predicted errors (solid lines) follow similar trends, with models using only 2-body descriptors showing higher overall error than those that combine 2- and 3-body terms. The figure suggests that the GP error tends to overpredict the true error, and this is mirrored by the results in Figure 26b: in this case, atomic environments near a vacancy defect were studied, and the GP model showed an overprediction for practically all environments, irrespective of their distance from the vacancy. The figure also illustrates how atoms near the vacancy (red) tend to have higher absolute force errors than those that are further away and therefore more bulk-like (blue). A more qualitative relation for predicted and actual *force* error has been shown for SOAP-like descriptors,^123−125^ enabling a different active learning method (Figure 13). In this case, the descriptor space was much larger, but the intended range of applicability was narrow, again making it practical to use a full GP based on a relatively small number of samples.

A more general study (including GPR as well as other types of ML methods) of uncertainty quantification with relevance to physical sciences was reported in ref (201). This study also includes a didactic overview of uncertainty quantification methods.

### 5.3 Committee Models and Uncertainty Propagation

Another approach to the determination of the uncertainty of a ML prediction involves the generation of a committee model,^205^ i.e. a collection of models that differ in the choice of hyperparameters,^206^ in the initialization of the fitting procedure,^129,207^ or in different subsampling of the training set.^202,208^ The gist of the idea is that the spread in the predictions can be linked to the reliability of the predictions: if changing details in the model leads to large changes in the predictions for a configuration, then this model is likely not trustworthy for that particular configuration.

If the different models are created by resampling the original dataset, there is considerable freedom in how that is done. One approach, commonly referred to as bootstrapping,^209^ keeps the size of each dataset the same as that of the original set, by randomly drawing data points from  while allowing replacement. The subsampling technique,^210^ on the other hand, creates datasets that are smaller than the original set and does not include replacement. It should be noted that bootstrapping introduces duplicate data points to the samples, thereby altering the distribution of the data points, whereas in subsampling individual predictions have larger uncertainty due to the smaller size of individual data subsets.

These ideas have been used for some time in the context of neural-network potentials^128^ but can also be shown to provide a rigorous estimate of the uncertainty,^211^ in a similar probabilistic sense as that given by the GPR variance. In fact, committee models are appealing for use in a GPR framework, particularly in combination with a sparse GPR model: evaluating the uncertainty entails a small overhead over a straightforward model evaluation, and it is simple to propagate uncertainty from the quantity that is directly predicted by the model to derived quantities that can be arbitrarily complicated combinations of predictions.

**Figure 27:**
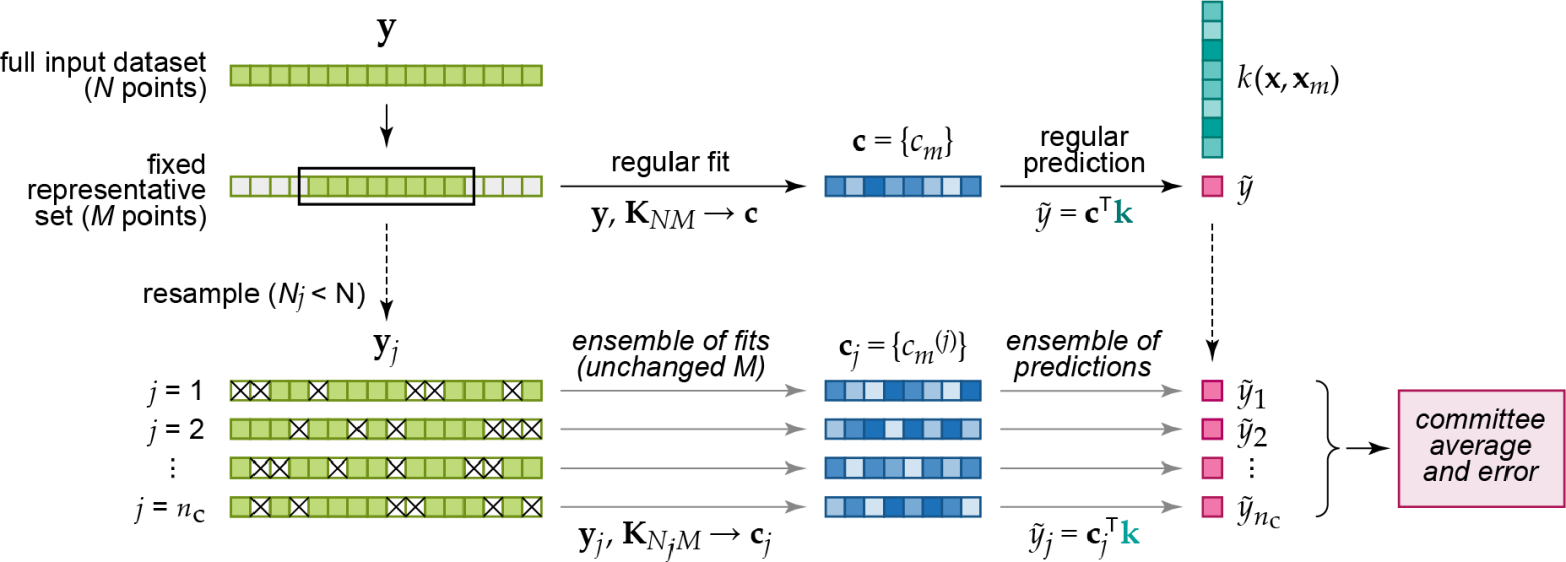
Schematic of the construction and use of a committee model for uncertainty estimation in sparse GP models, as described in ref (202). Similar graphical representations are used as in Figures 4 and 5: here, multiple models are trained using the same representative set but different random subselections of the training set, **y**_*j*_. The cost of training scales linearly with the number of committee members, *n*_c_, and each training yields a different weight vector, **c**_*j*_. When performing a prediction, a single vector of kernels, **k**, needs to be evaluated (which is usually the computationally intensive task for prediction), and multiple predictions, *ỹ*_*j*_, can be obtained cheaply by taking scalar products of **k** with the individual weight vectors corresponding to the members of the committee. Example applications of this methodology are shown in Figure 28.

Here, we discuss a simplified version of the uncertainty quantification framework discussed in ref (202), which is illustrated in Figure 27. Given an overall training set containing *N* configurations, and a representative set containing *M* reference environments, we perform *n*_c_ fits, keeping the representative set fixed, but extracting in each case a different random subset of the full training data to be used in each fit. This yields a collection of regression weights, {**c**_*j*_}. When a prediction is made for a new structure, one needs to compute a vector of kernels, **k**, between the new structure and the representative set. This is usually the time-consuming step, whereas the evaluation of *n*_c_ different predictions  is inexpensive. The possibility of computing all predictions with a single set of kernel evaluation makes the choice of building the committee by randomizing the training set much more efficient than the alternative option of randomizing the choice of representative points, which would be more directly analogous to randomizing the topology of a neural network (e.g., dropout), or by varying other hyperparameters.

**Figure 28:**
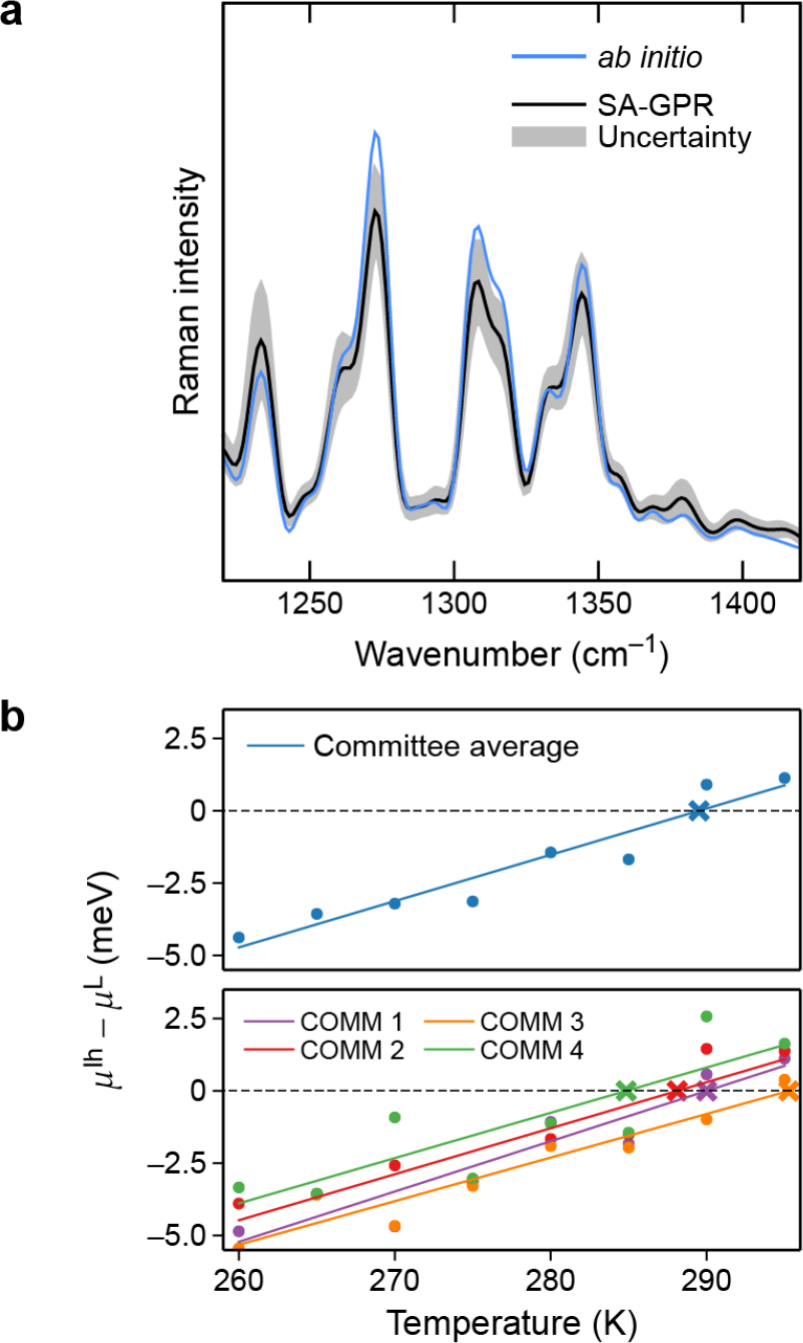
Applications of committee models for GPR predictions. Two examples are shown: (a) the prediction of the Raman spectrum of paracetamol form I;^203^ (b) the prediction of the melting point of water^204^ by determining the difference in chemical potential, μ, of hexagonal ice (“Ih”) and the liquid phase (“L”), and defining the zero intersect as corresponding to the melting temperature. Panel a is adapted from ref (203), where the original figure is published under the CC BY 3.0 license (https://creativecommons.org/licenses/by/3.0/); panel b is adapted from ref (204). Copyright 2021 AIP Publishing.

The set  constitutes the ensemble of predictions, and its distribution reflects the behavior of the model with respect to changes in the training set. For any atomic configuration, *A*, the mean and the variance of the ensemble
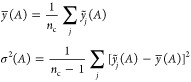
80can be taken to represent the best estimate and uncertainty. In practice one often finds that, similarly to the GP variance, this uncertainty estimate is qualitatively informative—small values being associated with good predictions and large values being associated with unreliable predictions—but not quantitatively accurate. In particular, there is a bias of the variance estimator for small *n*_c_. This bias can be reduced by introducing a scaling factor α, that can be computed by maximizing the log-likelihood of the model over a test set, {*A*}, which yields^204^
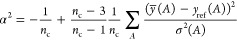
81The corrected ensemble variance is then obtained by redefining σ^2^ ← α^2^σ^2^. Furthermore, one can define “calibrated” committee models whose predictions are *ŷ*_*j*_ ←  + α (_*j*_ – ), which have the same mean as the initial committee, and an appropriately scaled variance. The advantage of this second approach is that one can then easily perform uncertainty propagation by computing a derived property, *F*, that depends on the model in arbitrary ways, by evaluating it on the calibrated model, *F*_*j*_ = *F*(*ŷ*_*j*_). This approach has been applied, for instance, to the prediction of Raman spectra together with the associated uncertainty (Figure 28a). These spectra are computed as the Fourier transform of the polarizability of the simulation box evaluated along the course of an MD trajectory.^203^ Another application has been the prediction of the electronic density of states (DOS) in amorphous silicon;^164,212^ details of this “ML-DOS” methodology are provided in section 7.4.

More recently, this inexpensive approach to obtain prediction errors has been put to use in practical applications to MD simulations. A common scenario entails the use of a baseline potential *V*_*b*_ (e.g., an empirical force field, or an approximate electronic-structure method), which is corrected using an ML model *V*_*δ*_(*A*) to define an overall energy *E*(*A*) = *V*_*b*_(*A*) + _*δ*_(*A*) that achieves the accuracy of more sophisticated, and expensive, electronic-structure calculations (cf. Figure 18a). In this case, one can use the committee error, σ(*A*), and an estimate of the RMSE *σ*_*b*_ of the baseline (relative to the accurate method), to define a *weighted baseline* potential
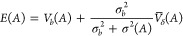
82that smoothly interpolates between the corrected potential *V*_*b*_(*A*) + *V*_*δ*_(*A*) when the ML model is predicted to be accurate and the bare baseline *V*_*b*_(*A*) when the predicted error is large.^204^ This improves the stability of simulations based on ML potentials and simplifies the iterative refinement of the model in all cases in which unexpected chemical reactions can occur, leading to structures that are not represented in the training set.

A second application involves the determination of the effect that uncertainties in the prediction of the ML potential have on thermodynamic properties that depend on the sampling of configurations, that is controlled by the potential-energy-dependent Boltzmann weight, *e*^–*βE*(*A*)^. An example application which employs on-the-fly reweighting^213,214^ of a single trajectory sampled according to the committee mean  is shown in Figure 28b. The spread in the prediction of the energy by the committee members translates into predictions of uncertainty in the ultimate property of interest—in this case the melting point of water. The conventional reweighting approach works by weighting the configurations in the trajectory driven by the committee average  by a factor *e*^(*E̅*(*A*)-*Ẽ*_*j*_(*A*))/*k*_B_*T*^, which makes it possible to compute averages as if the trajectory had been driven by _*j*_(*A*). This scheme works well only when the spread in the predicted energies of the committee is comparable to *k*_B_*T* throughout the trajectory. A more stable (albeit approximate) estimate of the error can be obtained with a cumulant expansion approximation,^214^ in which the averages ⟨⟩ computed using  are corrected based on the correlation between *y̅* and the logarithm of the weights, ((*A*) – _*j*_(*A*))/*k*_B_*T*. This reweighting scheme cannot be used to assess the error on *dynamical* properties, that are often computed from correlation functions of the trajectory generated by MD. To the best of our knowledge, the problem of error propagation to such observables has not been addressed, and the only possible, and rather time-consuming, strategy would be to generate separate trajectories using each individual member of the calibrated committee.

### 5.4 GPR Models for Isolated Molecules

Both in the present section and in the previous one, we have focused on ML models of strongly bonded, extended materials. This is where they had the largest impact early on, because empirical interatomic potentials for materials are in many respects rather poor models of the potential-energy surface. It is almost needless to say that molecular potential-energy surfaces are also an important area of application. The goal of the “first-principles” approach to making molecular force fields has always been the faithful reproduction of the potential-energy surface, assessed, e.g., by the accuracy of the vibrational spectrum or torsional “scan” around rotatable single bonds. For much of the long history of molecular modeling, the covalent bonding topology of a molecule has played a central role, giving rise to two constraints on the model:

(i) identification of the set of atoms that are bonded together and constitute the molecule;
(ii) the fixed network of covalent bonds used to define coordinates of the model (bond lengths, angles, etc.), and also to identify and differentiate between atoms according to the functional groups of which they are part (cf. “atom types”).

On the one hand, the formalism and approach that we introduced thus far in sections 2–4 can in principle be applied to molecules directly and lead to entirely *topology-free* models, and indeed this has been done and is particularly fruitful for molecular materials—we defer their discussion to section 6.6. On the other hand, there is a large body of modeling work that lifts the topological constraints of (ii) but keeps (i). In this case, an isolated molecule (or indeed a small cluster of molecules) with its constituent atoms is specified, but the model makes no further assumptions about the way in which the atoms are bonded together. The geometry is typically represented by the set of interatomic distances. A comprehensive and historical review is outside our scope here, but we note in passing the foundational works of Bowman and Braams^223^ that introduced permutationally invariant polynomials of the interatomic distances and the highly successful water model of Paesani and co-workers based on this formalism.^173,188,224,225^ A recent review of neural network models applied to the same problem is given in ref (226).

Recently, GPR has been employed for the same task by a variety of authors, fitting either the potential-energy surfaces directly or the difference between different levels of theory. We summarize recent works in Table 2, showing the system under study, the fitting target, the dimensionality of the potential-energy surface, and the efficacy; the last is indicated by a combination of the number of training configurations and the ratio of the energy RMSE to the range of energies in the dataset. The aim of the table is to give a sense of the complexity of these models, rather than to compare the works of different groups with one another directly. The modeling goals and the type of input data in each work were quite different, and the complexity of the task of fitting the potential-energy surface of different molecules, even if the dimensionality is comparable, can be quite different. Recent reviews of this topic are in refs (227 and 228).

**Table 2:** Overview of Recent GPR Models of the Complete Potential-Energy Surfaces for Isolated Molecules and Small Molecule Clusters^a^

Year	System	Target	Dimensions	Training set size	RMSE ratio
2013	2 H_2_O^174^	ΔMP2	12	9000	0.01
2013	2 H_2_O^174^	ΔCCSD(T)	12	1000	0.05
2016	N_4_^215^	CASPT2	6	1800	0.03
2016	CO_2_N_2_^216^	MP2	9	200	0.005
2017	H_2_S^217^	CCSD(T)	3	3700	0.0007
2017	2 HF^218^	MP2	6	300	0.001
2017	CH_3_Cl^219^	CCSD(T)	9	11000	0.0001
2018	H_2_CO_2_^32^	analytic	9	2500	0.00005
2018	H_3_O^+^^220^	CCSD(T)	6	10000	0.0002
2018	OCHCO^+^^220^	CCSD(T)	9	2600	0.0004
2018	H_2_CO^220^	MRCI	9	17000	0.0002
2018	2(HCOOH)^220^	CCSD(T)	24	9000	0.002
2020	H_2_CO^221^	CCSD(T)	9	3200	0.0001
2020	CH_3_Cl^177^	ΔCCSD(T)	9	2000	0.05
2020	C_6_N_4_H_9_^+^^222^	MP2	51	5000	0.003

a The last column shows the (rounded) ratio of the energy RMSE and the range of energies in the training dataset.

There is yet more GPR work on molecular potential-energy surfaces that did not quite fit into the table. Gradients can be fitted directly to aid geometry optimization.^229^ If the *ordered* matrix of interatomic distances is used as the representation, although constraint (ii) does not apply formally, the lack of permutation symmetry in the representation in practice limits the model to fixed bonding topology. Nevertheless, for this special case, highly efficient and accurate models can be created, e.g., to fit a dispersion correction,^230^ or more generally the potential-energy surface directly, as is done by Müller and co-workers, also reviewed in the present thematic issue.^231^

**Figure 29:**
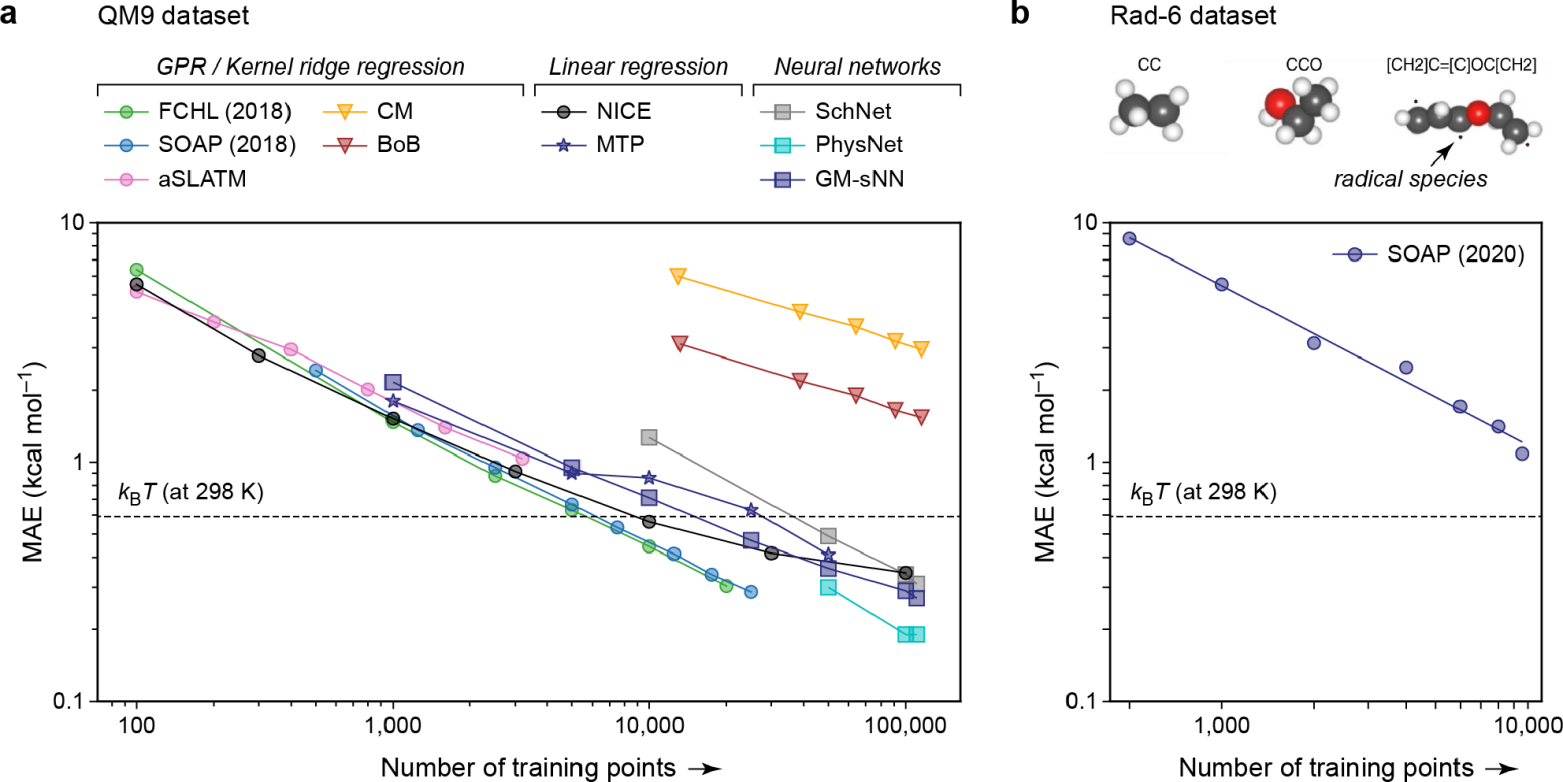
Performance of ML models for atomization energies of small organic molecules and radicals. (a) Learning curves for the QM9 dataset,^232^ using a variety of representations and regression methods. For each value of the training set size, we show the mean absolute error (MAE) evaluated on the test set which consists of the remaining structures from the full dataset. Models based on FCHL (2018),^233^ SOAP (2018),^69^ aSLATM,^234^ Coulomb Matrix (CM),^235^ and Bag-of-bonds (BOB)^236^ representations use Gaussian process/kernel ridge regression, whereas NICE^73^ and MTP^67^ use linear ridge regression, and SchNet^237^ and PhysNet^238^ are graph neural networks. GM-sNN uses a representation similar in spirit to MTP but based on a Gaussian radial basis set and a feed-forward neural network for regression.^239^ (b) Learning curve for the Rad-6 dataset.^240^ Example species are shown including a radical species, which actually account for over 90% of the total dataset (reprinted from ref (240); original work published under the CC BY 4.0 license; https://creativecommons.org/licenses/by/4.0/).

Finally, a modeling task entirely different from approximating potential-energy surfaces as a function of continuous atomic position is to predict static properties of new molecules with distinct bonding topologies. This is useful in high-throughput screening applications, e.g., in the pharmaceutical and organic semiconductor fields. A widely used benchmark to assess the efficacy of molecular representations and regression methods is the QM9 dataset of small molecules.^232^ In Figure 29, we show a recent set of results that includes a variety of GPR/KRR and neural-network models. Note that the quantity predicted in this benchmark is the DFT-calculated atomization energy of the molecules in their equilibrium geometry (as obtained using DFT), so none of these models in and of themselves are useful or practical for high-throughput screening, because the model input requires a DFT calculation (a full geometry optimization in fact) that already yields the target quantity. Nevertheless, the power of the density-based representations (FCHL and SOAP) combined with KRR/GPR is evident and suggests that it may be able to achieve other useful goals such as the fitting of correlated wave function theory based energy as a function of DFT-relaxed geometry.^151^ It is certainly the case that the QM9 benchmark has been very useful over the past years in refining descriptors and regression protocols, and the current crop of models perform significantly better than those from the same groups in earlier years.

One of the promises of ML force fields for molecules is that they will enable the accurate and routine construction of general *reactive* molecular force fields.^420^ There is scant research on this as yet, and Figure 29b shows that in comparison with closed shell molecules (such as those in QM9), describing open-shell radicals is much harder: the errors of the SOAP-based kernel model are three times larger on the Rad-6 dataset, which consists of all closed- and open-shell molecules containing C, H, and O with up to six non-hydrogen atoms.

## 6 Applications (I): Force Fields

The GAP framework is beginning to be applied to a variety of research questions in chemistry and materials science. The aim of the present section is to illustrate the step from the methodology (section 4) and its validation (section 5) to applications to practical problems, which are now beginning to emerge. The cases discussed below are therefore built on the premise that an accurate representation of a given potential-energy surface *has* been obtained and appropriately validated, and they highlight selected examples of what has been done with GAP models to date.

### 6.1 Transition Metals

Materials with crystalline order have long been successfully described with DFT, and larger-scale materials modeling frequently relies on computationally highly efficient empirically fitted potential models. There are cases, however, when neither of those options is practical: when the empirical potentials are too inaccurate to describe the specific (atomistic) materials-science problem that is being studied, yet DFT cannot reach far enough in terms of system sizes. ML-based interatomic potentials have emerged as suitable alternatives over the past decade—with applications to metals ranging from an early study of a structurally complex copper surface^128^ to simulations of compositionally complex high-entropy alloys.^243^

Tungsten was the first metal to be described by a dedicated multipurpose GAP.^121^ Owing to the applications of this metal in engineering, there are several important properties, ranging from the elastic constants and the formation energy of isolated vacancy defects to the delicate core structure of its screw dislocations.^244,245^ While properties such as the elastic constants can be derived from computations in small unit cells, and are therefore routinely obtained from DFT, other structural problems require thousands of atoms (and more) in the simulation cell. The GAP model introduced in ref (121) correctly describes the aforementioned core structure and can be used to study extended defects and their interaction using many thousands of atoms. This work has also been a prototype for how reference databases are constructed manually, guided by intuition and with specific applications in mind—adding, for example, vacancy or surface configurations and gradually improving the application scope of the resulting potentials (Figure 12).

**Figure 30:**
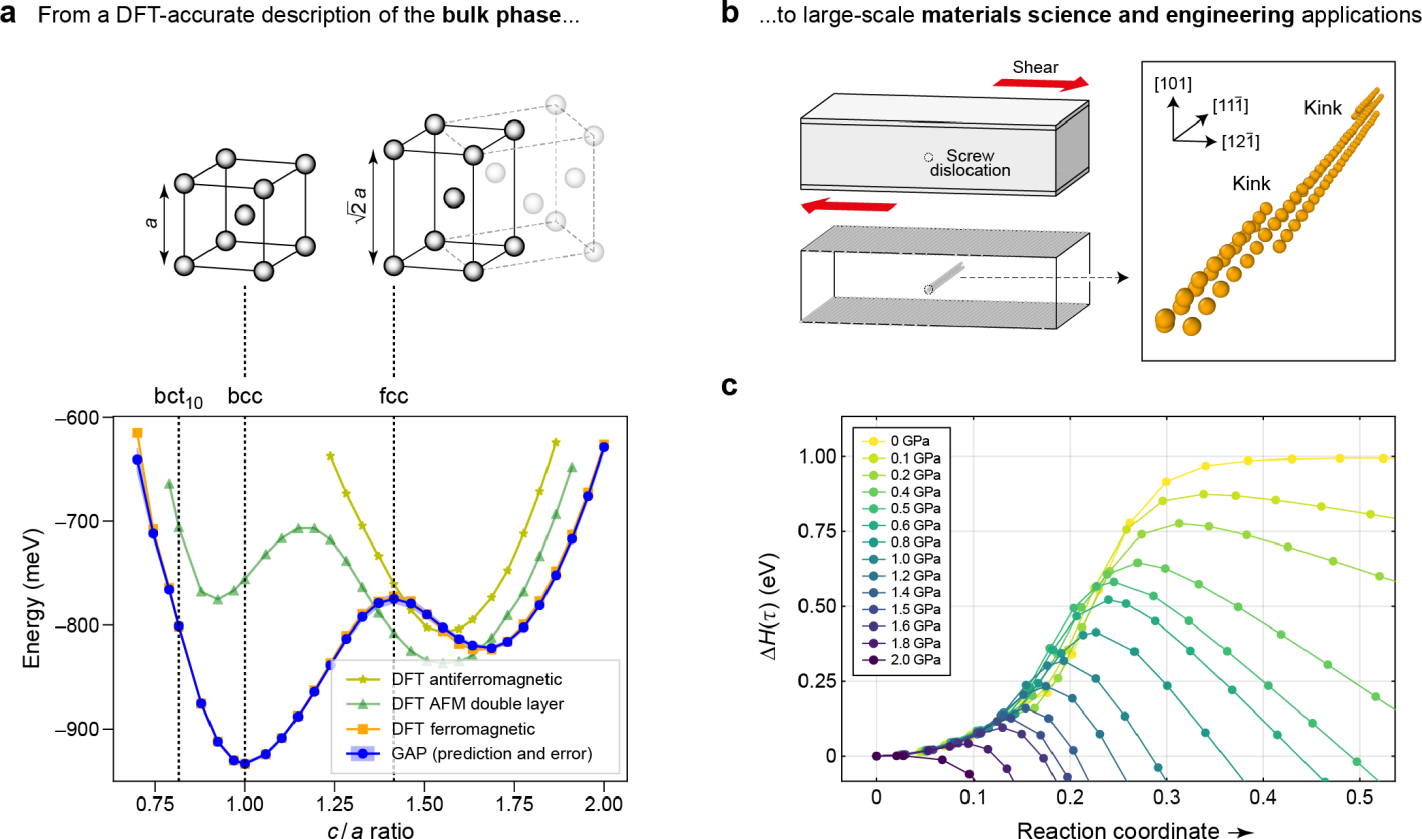
Applications of GAP to α-iron, from small unit cells (left) to large-scale simulations (right). (a) Energy for the Bain path, corresponding to the distortion of a body-centered cubic (bcc) unit cell to give tetragonal cells including one corresponding to a face-centered cubic (fcc) structure, as indicated. The results of spin-polarized DFT calculations for different magnetizations are shown, together with the prediction of a GAP model fitted to data corresponding to the ferromagnetic state. The GAP predicted error, indicated by shading, is mostly smaller than the line width, except around the  ratio corresponding to an fcc structure. Reprinted figure with permission from ref (241). Copyright 2017 by the American Physical Society. (b) Simulation setup for the computation of the double-kink nucleation barrier of a screw dislocation in a large periodic supercell model; details are given in ref (242). Adapted from ref (242), originally published under the CC BY 4.0 license (https://creativecommons.org/licenses/by/4.0/). (c) Minimum energy paths for the double-kink nucleation, drawn with data from ref (242): enthalpy change, Δ*H*, as a function of both shear stress, τ, and reaction coordinate.

The atomistic modeling of iron is notoriously difficult, partly owing to the magnetic nature of the ambient bcc phase. A GAP model fitted to ferromagnetic spin-polarized DFT calculations was shown to recover the energetic and temperature-dependent mechanical properties with high accuracy:^241^ a simple example is the Bain path (the tetragonal distortion of the body-centered unit cell, with *c*/*a* = 1 corresponding to the ground-state bcc structure, and  to cubic close packing), for which results from DFT and GAP are shown in Figure 30a. Later, the same potential was used in a study of the migration of the screw dislocation, in which the stress dependence of the Peierls barrier (double-kink nucleation barrier in this case) was calculated using a 50,000-atom system (Figure 30b–c).^242^ Furthermore, a software was developed for studying Fe grain boundaries and connected with the GAP model.^246^ We note that this potential is accurate for ferromagnetic iron at ambient temperatures, but it cannot simultaneously describe spin fluctuations and different magnetization states: that requires the incorporation of new, magnetic degrees of freedom.^247^ In other words, among the different DFT datasets shown in Figure 30a, only that for the ground-state ferromagnetic state has a corresponding GAP description.

As an example of an application at the other extreme of the temperature and pressure scale (where magnetism is suppressed), a GAP was developed to study liquid iron and sulfur under conditions corresponding to those at the Earth’s core: temperatures ranging from 4000 to 7000 K and pressures between 110 and 430 GPa.^248^ One of the objectives of that work was to study the partition coefficient of sulfur between solid and liquid iron. The GAP model reproduced the radial distribution functions of Fe, S, and Fe–S with high fidelity with respect to a DFT reference, as well as the melting curve of Fe. Having an accurate interatomic potential made it possible to carry out the large number of independent simulations (altogether comprising 10 M force evaluations on 180-atom unit cells) that were necessary for determining free energies at various compositions. In this application, the electronic entropy and its contribution to the free energy are significant, due to the high temperature. This required the construction of separate GAP models at each temperature point (in steps of 1000 K), fitted to DFT calculations which used the corresponding electronic temperature to determine the electronic free energy and Hellmann–Feynman forces. In the future, it would be desirable to incorporate the electronic temperature into the ML model itself explicitly, so that a single model would be able to predict properties corresponding to different electronic temperatures.

### 6.2 Complex Allotropy and Crystal-Structure Prediction

While most elements, particularly metals, have rather simple crystal structures, there are others which are much more complex: carbon, boron, or phosphorus are textbook examples. Such systems, even if comprising “only” a single elemental species, may pose outstanding challenges for force-field development, especially when multiple different allotropes are to be described at the same time. In return, elements with complex structures have turned out to be rewarding targets for the development of GAPs and other ML potentials, where the cost increase compared to empirical force fields may be justified by the gain in accuracy. For example, early ML-driven atomistic simulation studies of carbon allotropes were concerned with the description of the graphite–diamond coexistence line^116^ and, subsequently, with the nucleation mechanism of diamond in graphite under compression.^117^ These studies have been carried out with a neural-network potential following ref (66).

In cases where the structural diversity is large, and especially where previously unknown structures are to be explored, the requirements for ML potential development are shifted: rather than meV accuracy, one is primarily interested in having a robust potential that does not lead to unphysical behavior in simulations—only once that type of robustness is achieved, one will “focus in” on the structures of greatest interest. For example, a GAP for elemental carbon has been developed with a focus on amorphous phases and therefore describes a wide variety of structures including the coexistence of sp-, sp^2^-, and sp^3^-like carbon atoms over a wide range of densities^122^ (this model is referred to as “C-GAP-17” in the following). In contrast, a GAP for pristine graphene describes a much more limited configuration space, but at much higher accuracy.^249^ The tests for the latter potential included phonon dispersions at zero Kelvin as well as at elevated temperature,^249^ which provide an intuitive measure for the force accuracy of the fit. Numerical errors, given for in-plane force errors, are also instructive here: the graphene-specific GAP arrives at an RMSE of 0.028 eV Å^–1^ for its test set; C-GAP-17, a more widely applicable potential, shows a notably larger error in this test, viz. 0.27 eV Å^–1^, yet still outperforms all empirical potentials in terms of the same error measure (see details in ref (249)). A subsequent general-purpose carbon potential, “C-GAP-20”, extended the C-GAP-17 database with a large ensemble of manually constructed simulation cells representing defects in graphene, nanotubes, and other more complex structures.^152^ This potential was fitted to data computed at a higher DFT level than used for C-GAP-17, now including dispersion interactions which are important particularly in low-dimensional and sp^2^-rich carbon nanostructures. Tests in the initial work, as well as a separate, comprehensive benchmark study,^250^ confirmed the overall high accuracy that is afforded by this model. We note in passing that a similar approach has been applied recently to the isoelectronic and isostructural analogue of graphene, hexagonal boron nitride, and used for simulations of thermal rippling in large cells.^251^

GAP-RSS has been discussed in section 4.1.3 as an efficient way of exploring potential-energy surfaces and in section 4.1.5 as a proposed component in the development of “general-purpose” reference databases.^163^ There is, of course, now the question of how GAP-RSS may be *applied* in the next step, in a way similar to how AIRSS and related DFT-based structure-searching techniques have been used with great success to discover previously unknown structures and compounds.^4,137^

**Figure 31:**
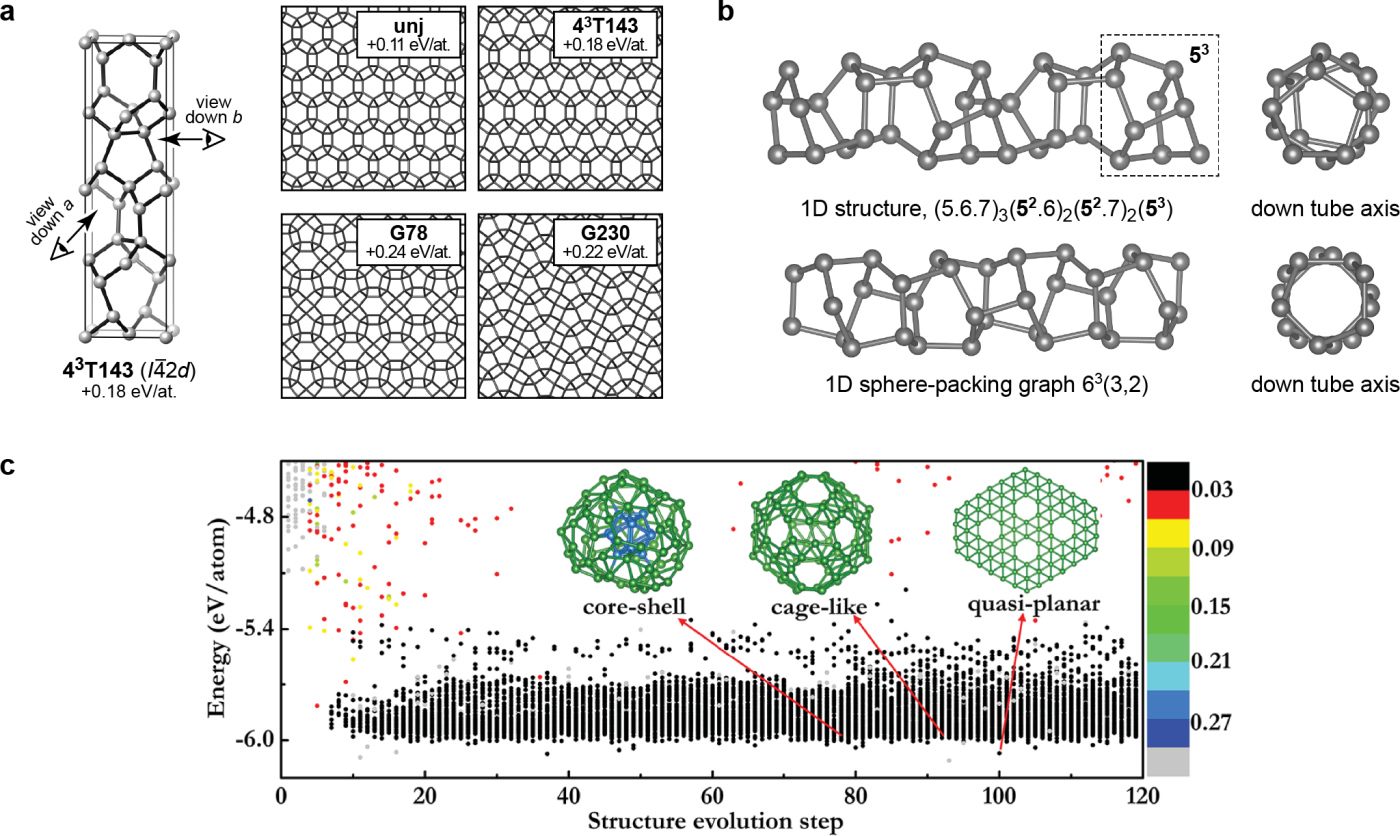
GAP-driven structure searching. Selected examples are shown for (a) hypothetical, crystalline carbon allotropes,^138^ (b) phosphorus nanowires,^142^ and (c) gas-phase boron clusters.^134^ GAP-driven modeling can speed up the global exploration of structural space by several orders of magnitude compared to purely DFT-driven computations and has been combined with existing approaches for structure search (panel b, AIRSS;^137^ panel c, CALYPSO^149^). Panel a adapted from ref (138), originally published under the CC BY 4.0 license (https://creativecommons.org/licenses/by/4.0/). Panel b adapted from ref (142), originally published under the CC BY 3.0 license (https://creativecommons.org/licenses/by/3.0/). Panel c republished with permission of The Royal Society of Chemistry, from ref (134); permission conveyed through Copyright Clearance Center, Inc.

Figure 31 illustrates three cases of structurally complex elemental systems that have been studied with GAP-driven structure searching in various implementations; it comprises bulk crystalline phases (Figure 31a),^138^ structures with low dimensionality (Figure 31b),^142^ and gas-phase clusters (Figure 31c).^134^ We focus on GAP below, but we note that more generally, the ways in which crystal structure prediction can be accelerated using machine-learned force fields (including various fitting schemes and their applications) have been reviewed in a recent perspective article.^141^

Early work was concerned with carbon, for which the prediction of hypothetical allotropes is a very active research area: see ref (252) and references therein. In 2017, it was shown how a GAP can be used to drive crystal-structure searching^138^—employing an approach similar to ab initio random structure searching (AIRSS)^136,137^ to generate a large ensemble of input structures, and subsequently relaxing these random structures, now using GAP. In this early study, the search was run by a potential that had not been fitted for any crystalline phase, instead including liquid and amorphous configurations in the reference database (which, of course, do cover diverse *local* structural environments). The work focused on all-“sp^3^” carbon allotropes by filtering the output of the search to only include those structures in which all atoms are 4-fold connected, and it allowed for the identification of multiple hypothetical structures that had not yet been included in the Samara Carbon Allotropes Database (SACADA)^252^ at that time. Two previously described hypothetical carbon allotropes (including the “chiral framework structure”, CFS,^253^ with **unj** topology), that were recovered in the GAP-driven search as well, are shown in Figure 31a. Below them, two related structures are shown that were identified by the GAP-driven search (indicated by the label “*G*” and a running index in Figure 31a).

For boron, there exist multiple crystalline allotropes,^254^ some of which are crystallographically disordered—most prominently rhombohedral β-B, which contains 105–108 atoms in the primitive unit cell, depending on how the structure has been described and refined (see ref (255) and references therein). The work in ref (140), which introduced the GAP-RSS method, led to a potential that could describe a variety of boron allotropes with close to DFT accuracy, including multiple supercell models of β-B with statistically distributed atoms on sites with mixed occupations.^140^ We note that a moment tensor potential was also developed for the boron allotropes by iterative structure searching and fitting and that that work identified further candidate structures for β-B.^127^

A related strategy, again using GAP fits and iterative exploration of the potential-energy surface, has been coupled to the CALYPSO particle-swarm optimization software for structure searching^148,149^ and has been applied to gas-phase boron clusters.^134^Figure 31c shows representative structures obtained in the process, including a cage-like cluster and a more stable quasi-planar structure. Very recently, an application of GAP- and CALYPSO-based structure searching to *bulk* phases of elemental boron was reported as well: the authors identified a possible metastable cubic B_24_ phase with an octahedral B_6_ unit as an additional structural feature.^256^

Phosphorus is similarly a case where a diversity of structural environments creates challenges for atomistic simulation. The most common forms are “white” (P_4_ molecular), “red” (amorphous), and “black” (puckered layers) phosphorus. But other phosphorus allotropes based on cage-like fragments also exist, and an even larger variety of such fragments has been studied in early computational work.^257^ Due to this structural diversity, new forms of phosphorus continue to be discovered: for example, nanotubular structures were described by Pfitzner and co-workers.^258^

An early GAP-RSS study dealt with phosphorus, showing how the structure of black phosphorus can be “discovered” and added to the reference database within a few iterations. It also included a proof-of-concept for the search for more complex, tubular structures, using an idea put forward by Ahnert et al.:^259^ rather than initializing the search with individual atoms, one would use entire fragments as the seed—in this case, phosphorus cages obtained from an information-theory-based decomposition of the structure.^259^ Based on this type of approach, the authors highlighted some candidate 1D and 3D structures (Figure 31b).^142^ A later study led to the prediction of a range of hypothetical, hierarchically structured phosphorus allotropes based on the simple P_8_ cage as a structural building block, including single- and double-helix forms.^260^

Phosphorus monolayers highlight again the importance of 2D structures, and several empirical force fields were developed specifically for phosphorene. Further, structurally more complex 2D materials include Hittorf’s (“hittorfene”), first predicted^261^ and recently experimentally realized;^262^ such structures may require more accurate computational treatment than fast empirical force fields can provide. Indeed, the GAP model of ref (163) is able to describe the exfoliation of hittorfene with high accuracy compared to DFT+MBD reference data. We also mention briefly the synthesis of nanostructures such as phosphorene nanoribbons,^168^ for which structural models are included in the reference database of that potential (cf. Figure 16).^163^

### 6.3 Structure of Amorphous Materials

Beyond the crystalline structures discussed so far, amorphous materials (i.e., those without long-range order) are natural targets for ML potentials, because they require highly accurate simulations over extended time scales and the use of large simulation systems—a requirement that cannot be met by established quantum-mechanical methods. In the following, GAP-driven simulations of amorphous solids will be briefly reviewed.

#### 6.3.1 Carbon Nanostructures

The structural and chemical diversity of elemental carbon is largely due to its ability to form 2-, 3-, and 4-fold-bonded environments (typically referred to as “sp”, “sp^2^”, and “sp^3^”, respectively, in a simplified notation). In amorphous carbon, these structural environments often coexist and their presence and relative abundance are controlled by external factors, such as the sample density.^264^ Among the many examples which require a more diverse description, we mention a computational study of the reversible graphitization in cold-compressed glassy carbon^265^ that used a state-of-the-art empirical potential model.^266^ The large structural diversity of amorphous carbon had motivated the development of the GAP-17 model for this element, and the initial work included tests for surface energies and the annealing of surface slabs (inducing graphitization at the surface at high temperature).^122^

**Figure 32:**
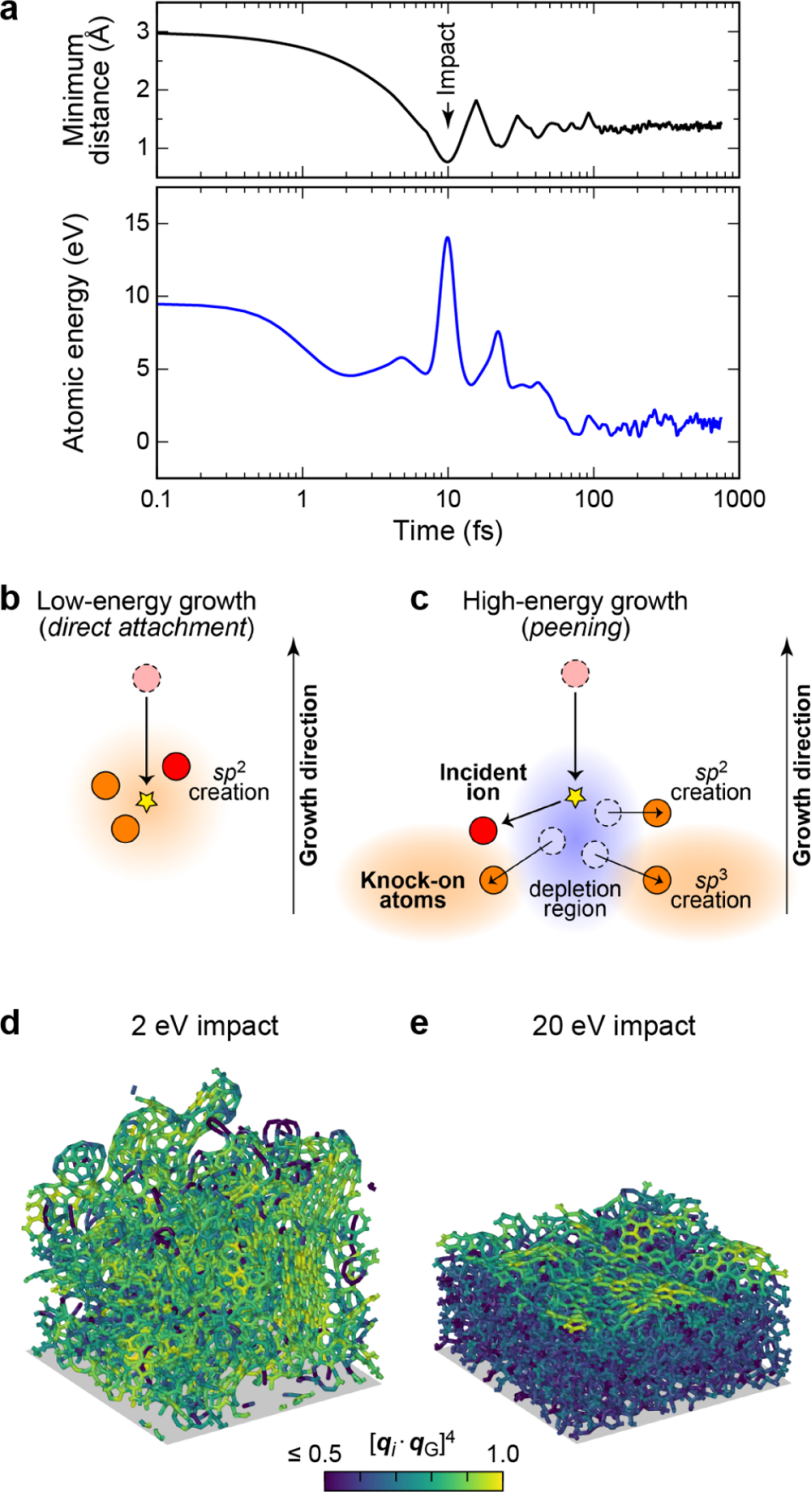
GAP-driven deposition simulations describing the growth of amorphous carbon films.^200,263^ (a) Example of an impact event: a carbon atom is placed 3 Å above the surface and given high velocity (corresponding to a kinetic energy of 60 eV). Within 10 fs, the atom impinges on the surface (corresponding to a spike in the energy of this atom; blue) and then comes to rest such that its nearest-neighbor distance is about 1.5 Å. (b, c) Schematic drawings of the proposed growth mechanisms at low and high impact energies, respectively, that are consistent with density changes over time observed in the GAP-MD simulations. Details, as well as quantitative data supporting these drawings, are given in ref (200). (d, e) Results of deposition simulations at two representative impact energies. The structure in panel d is a low-density, sp^2^-rich phase; that in panel e is a high-density, sp^3^-rich phase, in which only the surface region has substantial sp^2^ character (as determined by SOAP-based similarity and indicated by color coding). Reprinted with permission from ref (200). Copyright 2020 by the American Physical Society.

In 2018, the usefulness of GAP-driven simulations was demonstrated for deposition simulations of tetrahedral amorphous carbon (ta-C) films (Figure 32).^263^ Starting with a diamond-structured template, carbon atoms were accelerated toward the surface one after the other, with a kinetic energy corresponding to the energy of ions in deposition experiments (e.g., 60 eV). This type of deposition simulation is common in the carbon community but had previously fallen short of the experimentally observed sp^3^ count in ta-C (the latter reaching up to 90% in highly dense samples; see ref (264) and references therein). In contrast, the GAP-driven study recovered the experimental value.^263^ Furthermore, ML-driven atomistic simulations can not only create accurate structural models but also give insight into the mechanisms by which these structures form. In the case of carbon, there had been an ongoing debate in the literature as to which of several competing growth mechanisms is responsible for the formation of highly sp^3^-rich ta-C films (see references in ref (263)). GAP-driven simulations led to density profiles (averaged over many individual impact events) which are consistent with the “peening” mechanism proposed earlier by Marks^267^ based on simulations with the environment-dependent interaction potential for carbon (C-EDIP):^266^ a high-energy atom displaces atoms from the impact region and leads to a net *depletion* of sp^3^ density directly at the impact site; in contrast, the film grows laterally, around the impact site, where the sp^3^ count increases. The study was subsequently extended to cover a wide range of impact energies, demonstrating that a diverse type of film structure can be obtained as dependent on the impact energy (two examples are shown in Figure 32d–e).^200^

Deposition simulations are computationally demanding, and the more common way to obtain atomistic structural models of a-C (and ta-C) is given by rapid simulated quenches from the liquid state. A detailed study of structural and elastic properties of different a-C networks, obtained by quenching, was carried out by Jana et al., who compared simulations using an existing empirical potential with simulations using the C-GAP-17 model.^268^ Another study included the generation of many individual a-C model structures by GAP-driven quenching and a subsequent link to experimental properties.^269^ A computational study of plasticity in large structural models of a-C, again using a combination of GAP and a faster empirical potential, was reported in ref (270).

Another direction in the atomistic modeling of carbon materials is the thermal annealing of more disordered structures to gradually generate more ordered ones, following an early study in 2009 that used C-EDIP.^271^ There is a recent benchmark study of various interatomic potential models for carbon^272^ which used such annealing simulations for a series of tests, including C-GAP-17 and C-EDIP, and demonstrating good performance of both potentials (for example, in terms of the description of the graphitization process) compared to other, often simpler empirical interatomic potentials. Simulations of this kind have given rise to structural models of carbonaceous energy-storage materials,^273,274^ which will be discussed further below.

#### 6.3.2 Amorphous Silicon

Silicon is perhaps the most canonical covalent amorphous system, and its structure has often been approximated as an ideal tetrahedral random network in which all atoms have a coordination number, *N*, of four.^276,277^ However, defects (commonly defined as atoms with either *N* = 3, “dangling bonds”, or *N* = 5, “floating bonds”) are important as well,^278^ and the description of these defects can be challenging. An early work dealt with GPR models for energetics in silicon.^279^

In 2018, GAP-driven MD simulations were reported that generated a-Si structures by quenching from the melt,^193^ varying the quench rate over a wide range. A main advantage of the ML-driven methodology is not only in the accessible system sizes but also the accessible time scales, because the slow quenching and optimization of a-Si structures can be computationally demanding (see ref (280) for the recent DFT-based generation of a highly relaxed a-Si structure). The enthalpy of the amorphous network with respect to the more stable diamond-type crystalline phase, experimentally measured by calorimetry, is directly related to how ordered (that is, how well annealed) a given sample is, and the excess energies of simulated quenched a-Si samples obtained with quench rates between 10^13^ and 10^11^ K/s are consistent with experimentally reported excess enthalpies. These findings were later independently corroborated by a study with neural-network potentials.^281^ Using a series of progressively slower quenches for 512-atom a-Si systems, it was shown subsequently that slower quenching (10^10^ K/s in the relevant part of the simulation) does not seem to further lower the overall potential energy compared to a 10^11^ K/s quench—but it still increases the medium-range order, as measured by the count of six-membered rings.^53^ Concerning the question of how structural models of amorphous materials may be validated, which is a highly nontrivial task, ref (193) also included comparison with previously reported experimental data for ^29^Si NMR chemical shifts and structure factors from diffraction (see ref (193) and references therein).

**Figure 33:**
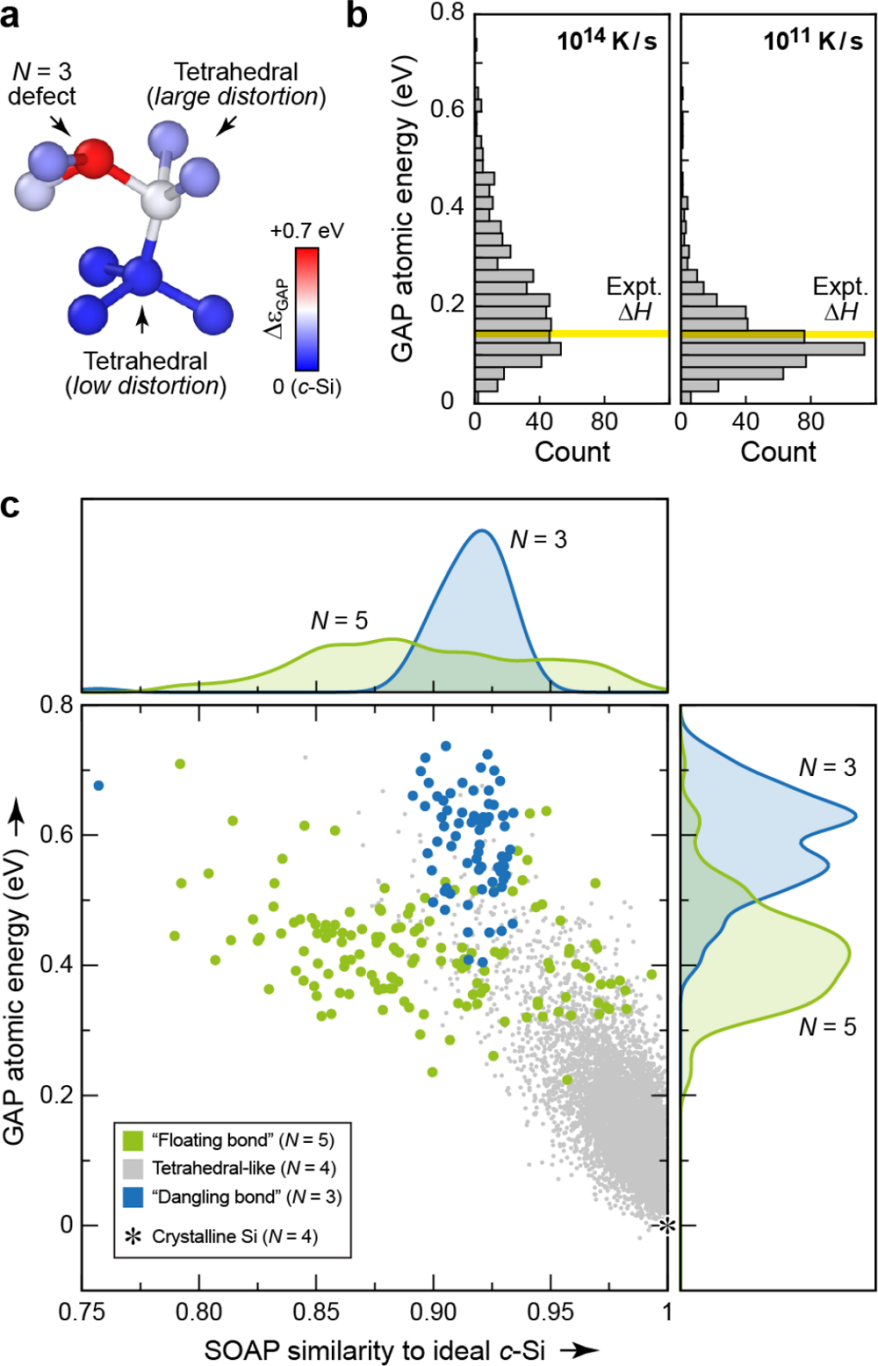
Machine-learned atomic energies in amorphous silicon (a-Si) as obtained from GAP regression models. (a) Example snapshot from an a-Si structure obtained by GAP-driven melt–quench simulations (see ref (275) for details). Atoms are color-coded according to atomic energies, referenced to crystalline silicon (c-Si). (b) Distributions of atomic energies in two a-Si systems obtained by quenching at a very fast (left) and slower (right) rate. (c) Two-dimensional plot of structural similarity to c-Si (horizontal axis) and atomic energy (vertical axis) for atoms in an ensemble of a-Si structures. The plot focuses on atoms with *N* = 3 and *N* = 5 neighbors, for which data are shown by larger symbols. Adapted from ref (53). Original figure published under the CC BY 4.0 license (https://creativecommons.org/licenses/by/4.0/).

The local atomic environments in a-Si were studied in terms of their energetics, as derived from GAP regression models, as well as structural properties (Figure 33).^53^ This study demonstrated that to some extent, the atomic energies from GAP can be interpreted in a chemical way (we note that a counterpoint for neural network potentials has been made in ref (282)). Second, it provides an explanation for the initially rather counterintuitive finding that defective a-Si networks can be energetically slightly favorable compared to those generated by the WWW algorithm^276^ leading to a “perfect” random network and subsequent DFT relaxation of both structures.^53^ The key finding is that while the 3-coordinated atoms are generally strongly unfavorable energetically, there exist 5-coordinated atoms which are *more* favorable than highly strained 4-coordinated atoms. This is another manifestation of the limitations of assigning atomic environments based on coordination numbers only (see ref (283) for a discussion in the context of ta-C and SOAP analysis). The energetic analysis was corroborated by studies of the electronic structure, particularly the local density of states resolved according to different *N*-coordinated atoms, revealing a fundamentally different character of the different local environments.^53^

In the context of atomic energies from GAP models, as characterized in Figure 33, it is worth noting a recent study in which those local energies were correlated with structural aspects of local distortions (“distortion scores”), with higher local energies corresponding to larger distortion scores.^284^ It was also suggested to combine the GAP atomic energies with a pressure-dependent term to arrive at an atomic *enthalpy*; see ref (164). Further investigations of the information that can be extracted from such atomic energies and enthalpies would seem worthwhile.

#### 6.3.3 Ge–Sb–Te Phase-Change Materials

Ge–Sb–Te phase change materials (PCMs) are important components of data storage and processing technologies^287^ and also relevant for emerging applications in photonics.^288^ The reason for this importance is a pronounced property contrast between crystalline and amorphous phases, which needs to be understood on the atomistic scale. DFT-based simulations have been a key technique in understanding and optimizing PCMs,^5,289,290^ but such simulations have only been able to address relatively small system sizes. Indeed, among the most extensive ones are a DFT-based simulation comprising 900 atoms^291^ and a report of simulations spanning over 8 ns but using smaller systems.^292^ Consequently, ML potentials are playing an increasingly important role in the field. Foundational early studies have been carried out for GeTe as a prototypical phase-change material, for which artificial neural-network models have been developed and applied by Sosso, Bernasconi, and colleagues.^293−297^ For example, the authors studied the thermal transport in the material^294^ and described the crystallization behavior of bulk^295^ and nanowire^296^ structures.

**Figure 34:**
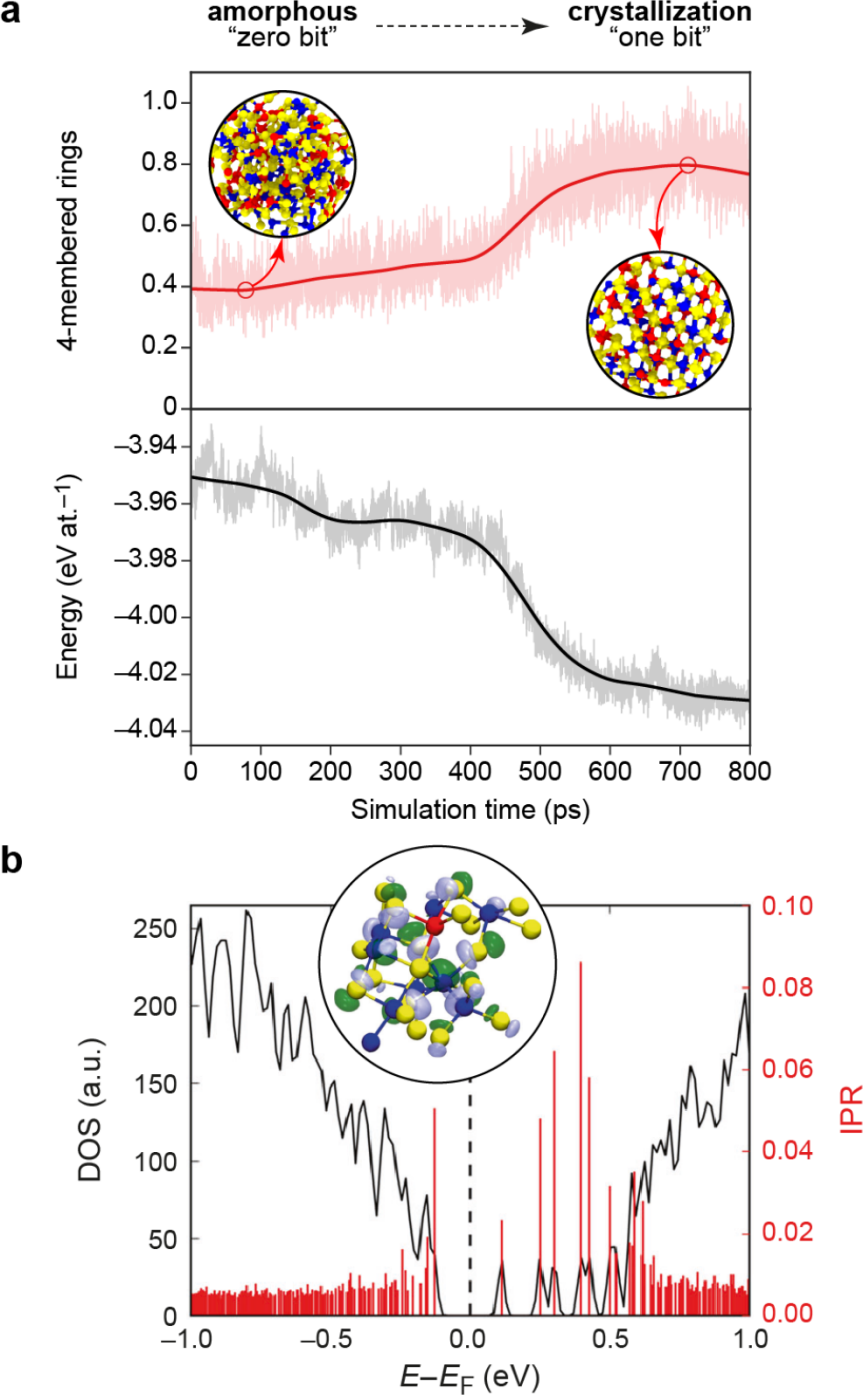
GAP-driven modeling of the Ge_2_Sb_2_Te_5_ phase-change memory material. (a) Partial crystallization of amorphous Ge_2_Sb_2_Te_5_. The upper panel traces the increasing structural order, quantified using the number of four-membered rings with “ABAB” alternation (as in the rocksalt-type structure). Representative structural fragments are shown and illustrate the transition from a disordered amorphous (left) to a partially crystallized (right) structure. The lower panel shows the potential energy of the system (as obtained from the GAP model), which indicates a stabilization during crystallization, as expected. Reprinted with permission from ref (285). Copyright 2018 American Chemical Society. (b) Electronic structure of a 900-atom structural model of amorphous Ge_2_Sb_2_Te_5_,^286^ obtained from a GAP-MD simulation, illustrating the synergy between large-scale GAP-MD and single-point electronic-structure computations. The inset shows a structural fragment and visualizes the electronic structure of the midgap state associated with it. Isosurfaces of the wave function amplitude are shown at an isovalue of ±0.015  Adapted from ref (286). Original figures published under the CC BY 4.0 license (https://creativecommons.org/licenses/by/4.0/).

In 2018, Mocanu et al. reported a GAP model for Ge_2_Sb_2_Te_5_, fitted to liquid and amorphous configurations of the ternary compound as well as structures of the constituent crystalline phases.^285^ Comparison of GAP-MD with DFT-MD data as well as experimental reference data indicated a good performance for liquid and amorphous Ge_2_Sb_2_Te_5_, assessed, for example, in terms of the description of the structure factor, and the potential was demonstrated to describe the formation of ordered, crystalline regions from an amorphous structure upon annealing (Figure 34a). This potential was furthermore used to generate multiple relatively small structures in parallel, which were then analyzed using first-principles DFT in regard to their bonding properties.^285^ Initial simulations for a 7,200-atom system were also reported—thereby demonstrating how Ge_2_Sb_2_Te_5_ may now be studied in much larger simulation cells than would be accessible to DFT-MD.^285^ Subsequently, simulations with simulation-cell sizes up to 24,300 atoms were carried out using the same potential, systematically addressing the role of the simulation-cell size as well as that of the quench rate on the resulting structures.^298^

In 2019, Konstantinou et al. described a study of the role of midgap states in amorphous Ge_2_Sb_2_Te_5_, which further emphasized the usefulness of combined GAP-driven modeling and electronic-structure analyses.^286^ In this case, hybrid-DFT level computations were used to study the nature of midgap states in amorphous Ge_2_Sb_2_Te_5_, which are of central importance to the electronic properties of the amorphous “zero bits”. The availability of the computationally efficient GAP model allowed the authors to generate a large ensemble of structural models to serve as input for the subsequent electronic-structure analysis. An electronic DOS analysis of such a (GAP-generated) structure is highlighted in Figure 34b,^286^ and a detailed discussion may be found in the original work in ref (286).

Recent studies were concerned with the supercooled liquid phase as described by GAP-driven MD,^299^ assessed by comparison with experimental data from ref (300) and with the application of the Ge_2_Sb_2_Te_5_ model to the end member of the quasi-binary line, Sb_2_Te_3_.^301^ The latter work is a case study in transferability: studying liquid and amorphous Sb_2_Te_3_ takes the potential away from the region of configuration space for which it was initially fitted. It is emphasized that the reference database for the potential contained liquid and amorphous Ge_2_Sb_2_Te_5_, in which the local environments of Sb atoms are expected to partially resemble those in Sb_2_Te_3_ because of the chemical relationship between the phases, but they will be different in detail (especially beyond the first neighbor shell).

Ge–Sb–Te materials are an excellent example of how the structural properties are directly linked to practical applications; more details of this are given in a subsection below. It is worth mentioning at this stage, however, that thermal properties of PCMs are likely to be of interest in the future, following early work on binary GeTe that used a neural-network potential.^294^ Indeed, a recent study used a GAP to simulate temperature-dependent vibrational properties in GeTe based on long time scale MD simulations.^302^

### 6.4 Surface Chemistry

Real materials are not infinitely extended, and the study of material surfaces opens up a further degree of structural complexity. Take diamond-type silicon as an example: the bulk crystal has a simple diamond-type structure, whereas the most stable (111) surface structure is a complex (7 × 7) reconstruction—and its description by DFT computations has been an important milestone.^303^ Similarly, the silicon (111) surfaces and their reconstructions have served as a testing ground for GAP models.^139,151^

Even more complex structures are found at the surfaces of amorphous materials. For example, surfaces of amorphous carbon have been studied in light of its applications in coatings and chemical sensing; an overview of applications of those materials in biosensing was given in ref (304). Recent work in ref (275) introduced a library of surface slab models, generated by cleaving from bulk ta-C samples and subsequent thermal annealing to “heal” dangling bonds at the surfaces. A systematic study was carried out of the structural properties as dependent on the system size, assessing the question of what size of simulation cell would be required to reliably describe ta-C surfaces. A system size of 216 carbon atoms per cell was found to be a reasonable choice. Because the carbon GAP in this case was fitted only for bulk elemental carbon,^122^ the authors showed how its simulation outcomes (here, the annealed ta-C slabs) can be further coupled to density-functional based modeling to access a larger chemical space (here, that of hydrogen- and oxygen-based functionalization which is relevant for practical applications). Specifically, the hydrogenation of slabs was described by grand canonical Monte Carlo simulations using density-functional tight-binding models, which require less computational effort than DFT and therefore allow for the evaluation of many individual configurations—up to reaching a hydrogen content of about 30%, consistent with experimental samples. On the other hand, oxygenation involves much more complex surface reactions and an interplay between, for example, epoxy and carbonyl groups; simulations of this type (again starting from the GAP-generated ta-C slabs) were therefore carried out using DFT-based *ab initio* MD simulations.^275^

Further analysis of the surface structures was carried out in a companion paper.^283^ The use of “sp^2^” and similar labels was compared with the outcome of a SOAP-based clustering technique. The latter identified a number of typical environments that are taken to be representative of different types of bonding in a-C materials: for example, an atom with *N* = 2 nearest neighbors might be either in a linear (−C≡C−) or in a defective sp^2^-like environment, and the SOAP-based analysis separates these two types of environments to a good degree. The work also exemplified the ability to include properties beyond the atomistic structure in the construction of kernel-based models. Specifically, the authors “encoded” electronic-structure fingerprints through the moments of the local density of states and used those to construct a second kernel that separates environments based solely on their electronic (and thereby, bonding) nature. Combining this kernel with a SOAP term to include the atomistic structure, Caro et al. demonstrated an improvement in the prediction of hydrogen adsorption energies (as a simple proxy for chemical reactivity) as compared to a pure SOAP-GPR model.^283^

Aarva et al. predicted X-ray photoelectron spectroscopy (XPS) and X-ray absorption spectroscopy (XAS) fingerprints based on GAP-generated and DFT-functionalized structural models,^305,306^ in another demonstration of how one may interface atomistic structure to high-level computations. Because the reference computations that predict the spectra are computationally expensive, it is crucial to carefully select those (relatively few) configurations for which computations are to be done—this was achieved using SOAP-based clustering, similar to ref (283). This methodology is beginning to be used to fit experimental X-ray spectra, as demonstrated in ref (307).

**Figure 35:**
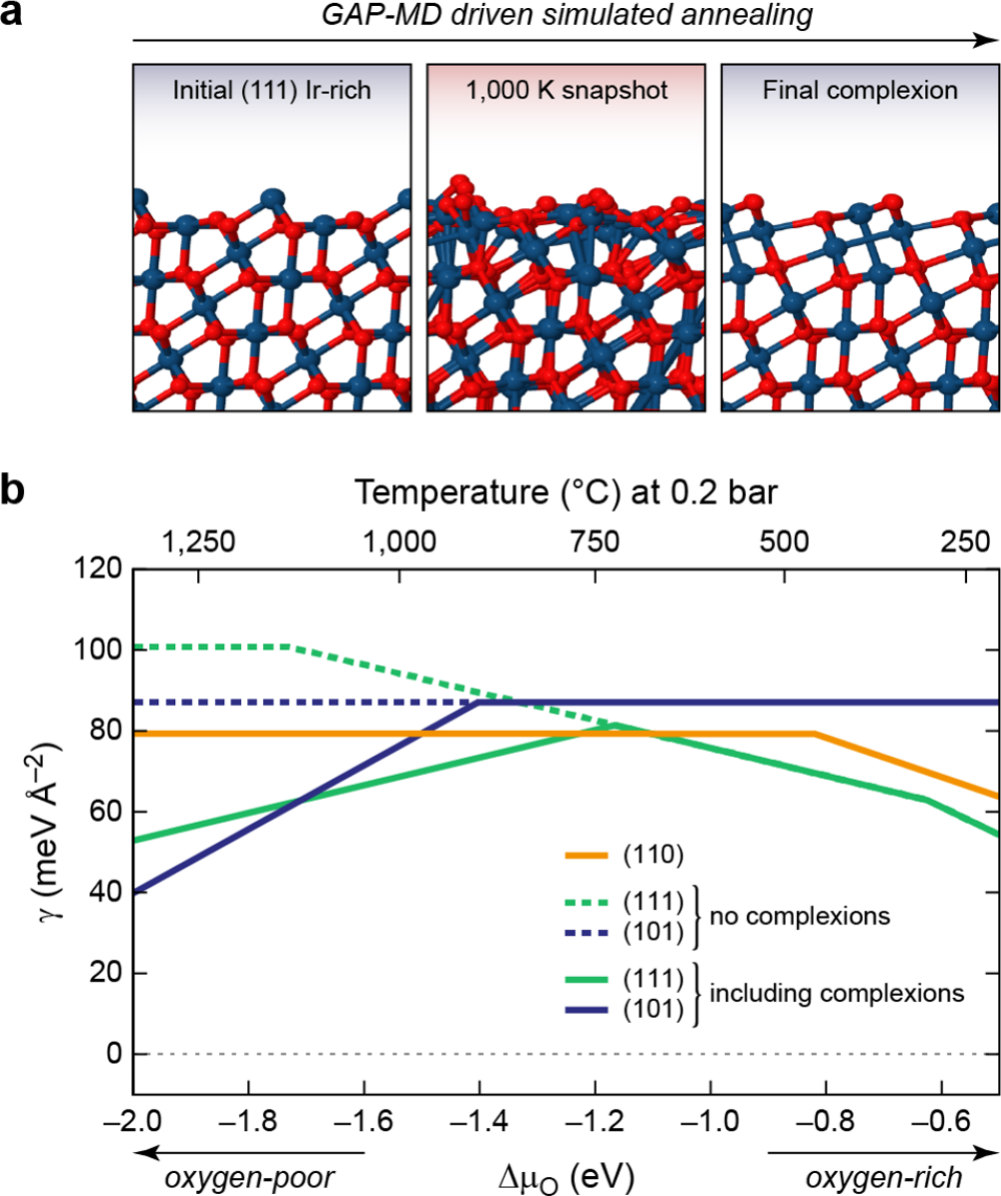
Modeling oxide surfaces with GAP. (a) Surface complexions for the (111) surface of IrO_2_, showing the initial structure, a snapshot at 1000 K, and the final equilibrium structure after simulated annealing. Reprinted with permission from ref (308). Copyright 2020 by the American Physical Society. (b) Surface free energies, γ, as a function of oxygen chemical potential, Δμ_O_, for three different surfaces. Dashed lines indicate the surface free energies obtained without complexions. Adapted from ref (308). Original work copyright 2020 by the American Physical Society.

Returning to crystalline phases, a recent study reported on IrO_2_ surfaces including various complexions, described by a GAP model.^308^Figure 35 shows the newly discovered metal-rich surface complexions, obtained using simulated annealing, and their corresponding surface free energies. ML-potential-based simulations of this type build upon DFT-based ab initio surface studies which are firmly established in the field.^309^ With greatly improved computational speed, one may now envision pushing the limits of such methodology even further: for example, to the exploration of much larger possible surface reconstructions (just like the (111)-(7 × 7) reconstruction of silicon, which searches in smaller unit cells would not have found, but a recent study did using an ML potential^310^), and to the prediction of the equilibrium shape of nanoparticles (based on Gibbs–Wulff constructions) with complex compositions at finite temperatures. Finally, with improved information about which specific surface is expected to form, one may extend the simulation study from a free surface to one with a molecule attached or to an entire catalytic reaction system.^311^

### 6.5 Functional Properties

A next step in the application of ML potentials, including GAP, is to move from structure to functionality, i.e. to material properties which are directly related to a practical application. A very recent example is given by amorphous silicon, for which structural studies were mentioned in section 6.3.2: Wang et al. experimentally investigated the behavior of a-Si samples under tension and compression, finding a much stronger tensile than compressive strength, and corroborated their mechanical measurements with atomistic simulations including GAP-18.^312^

The transition between different solid phases is an interesting challenge for ML-driven modeling, especially when the process involves very diverse local environments. The previously mentioned PCMs are a typical example of this, and crystallization simulations have initially been carried out with a GAP model for Ge_2_Sb_2_Te_5_. Figure 34a illustrates the partial crystallization of Ge_2_Sb_2_Te_5_ using the count of 4-fold rings as a measure for crystallinity:^313^ this value is expected to be unity in a perfect rock salt-type structure. The energy of the system, accordingly, is lowered notably during the crystallization, by almost 0.1 eV per atom. In terms of PCM applications, this simulation mirrors the SET process (amorphous → crystalline transition; see ref (5)).

The transport of heat in a crystalline or noncrystalline system is a critical functional property in thermoelectric waste-heat recovery. In principle, ML potentials are well suited to speed up predictions of such properties, because the latter are again derived directly from the PES; applications of ML potentials to the thermal properties of amorphous phases have been reviewed.^314^ The prediction of thermal properties for crystals in the GAP framework was exemplified for zirconium.^315^ Two separate studies discussed the thermal properties of crystalline, diamond-type silicon.^196,316^ A separate potential was fitted to describe the thermal conductivity in silicene.^317^ Finally, a GAP model was developed for the β polymorph of Ga_2_O_3_, specifically with a view to describing the vibrational and thermal properties.^182^

Materials under irradiation are exposed to extreme conditions, and accordingly the resulting atomistic structures are often very far from equilibrium. Until now, the interatomic repulsion at short distances has mainly been discussed as a qualitative feature of the PES that needs to be taken care of but is not the main subject of study.

**Figure 36:**
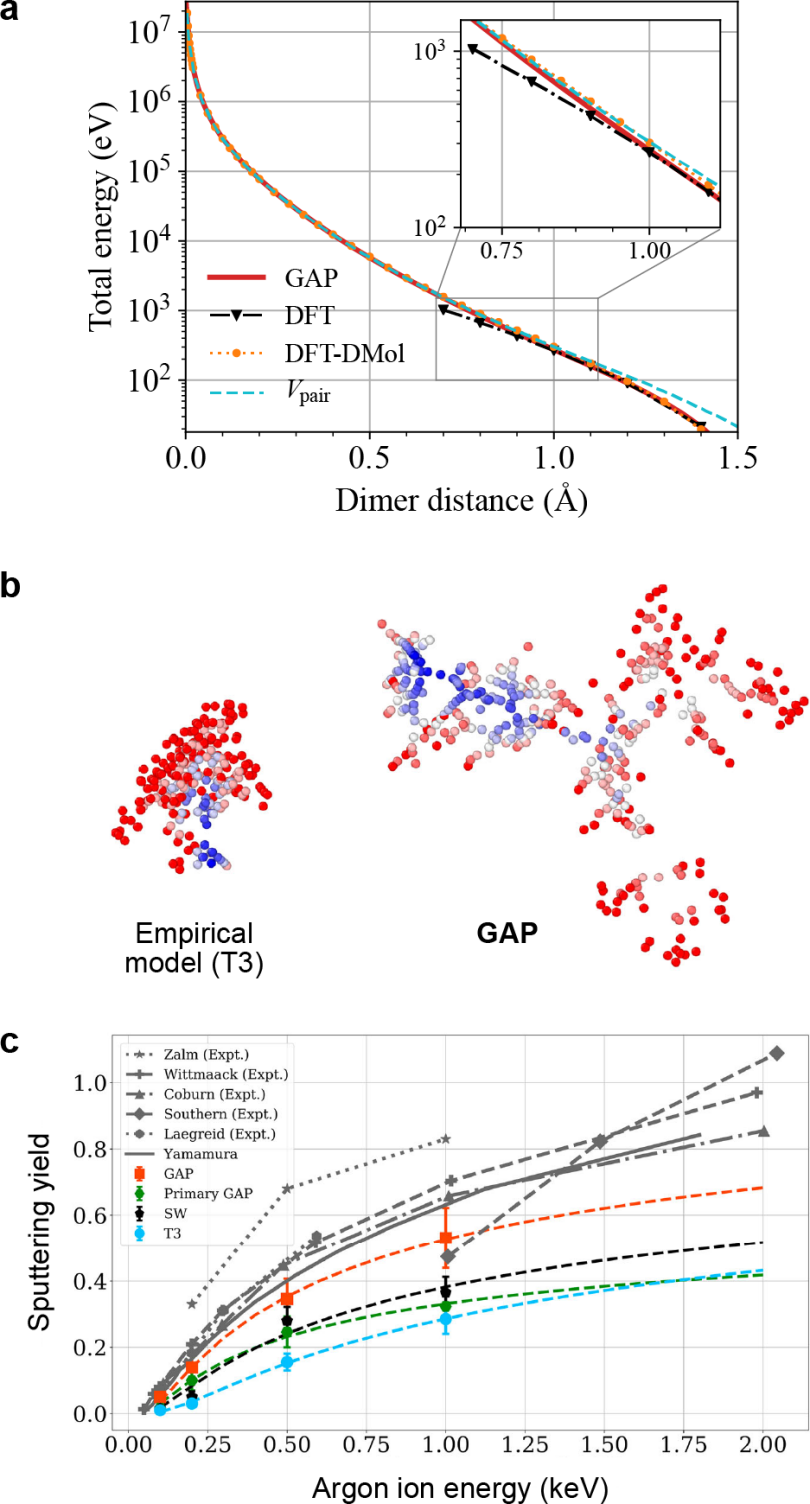
Simulations of matter under extreme conditions using GAP: here, exemplified by radiation damage. Panel a shows the construction of a GAP for tungsten with a repulsive term at very short interatomic distances, for which reference data are computed using DMol rather than the standard DFT method;^318^ note the energy scale reaching up to MeV energies. Reprinted figure with permission from ref (318). Copyright 2019 by the American Physical Society. Panel b is a selected example of a single impact event in silicon, simulated by an empirical interatomic potential (left-hand side) and a GAP (right-hand side).^319^ Only defect atoms are shown, with the color corresponding to the time the defect was generated, referenced to the primary impact event. Panel c shows the sputtering yield obtained in simulations with various force fields compared to experimental data (gray). The GAP model containing a pair potential term that is repulsive up to MeV energies (red) predicts a notably higher sputtering yield than the other force-field models. Reproduced (adapted) from ref (319); original figures published under the CC BY 4.0 license (https://creativecommons.org/licenses/by/4.0/).

In radiation damage studies, however, a *quantitatively* accurate description of interatomic repulsion down to very small interatomic distances is required, because it is there that the relevant microscopic processes are taking place. Accordingly, the energies of the repulsive potential range to the MeV region (millions of times more than a typical covalent bond energy). A recently developed GAP model for tungsten^318^ recovers this behavior accurately because it has been specifically extended to describe such small interatomic distances (Figure 36a)—similar GAP models were later developed for a range of refractory metals.^320^Figure 36b provides a comparison of two selected high-energy events as described by an empirical (Tersoff-III, “T3”) potential^321^ and the authors’ GAP for Si.^319^ While they both focus on one individual event out of a presumably wide distribution (and different empirical potentials will again differ from one to another; ref (319)), the authors’ results clearly suggest that the processes described in these two simulations are qualitatively different. The absence of physical constraints on the shape of the interatomic interactions may contribute to allowing the atoms to travel highly complex pathways in the case of the GAP-based simulation, characterized on the right-hand side of Figure 36b. The same groups recently published a study of such high-energy collision events in molybdenum.^322^

Figure 36c includes a comparison with GAP-18 (labeled there as “Primary GAP”), which performs worse than the tailor-made potential, yet still on par with a range of traditionally used empirical force fields. This may be an important guiding point for the future construction of general-purpose GAPs: even in extreme situations, one would like them to revert *at least* to the physical behavior of empirical force fields. The methodological steps required for this relate to all three key components of GAP model fitting (cf. Figure 11): (i) the development of appropriate reference databases which must include relevant environments in small simulation cells (as does, for example, the C-GAP-17 model for carbon which contains the results of small-scale surface simulations at very high temperature^122^); (ii) the construction of suitable atomistic descriptors which may include 2-body and other terms in a hierarchical way (cf. section 4.2); and (iii) the appropriate control of input (regularization) and output (uncertainty quantification) in the GPR model, both at the fitting stage and at the stage when the simulations themselves are being carried out.

**Figure 37:**
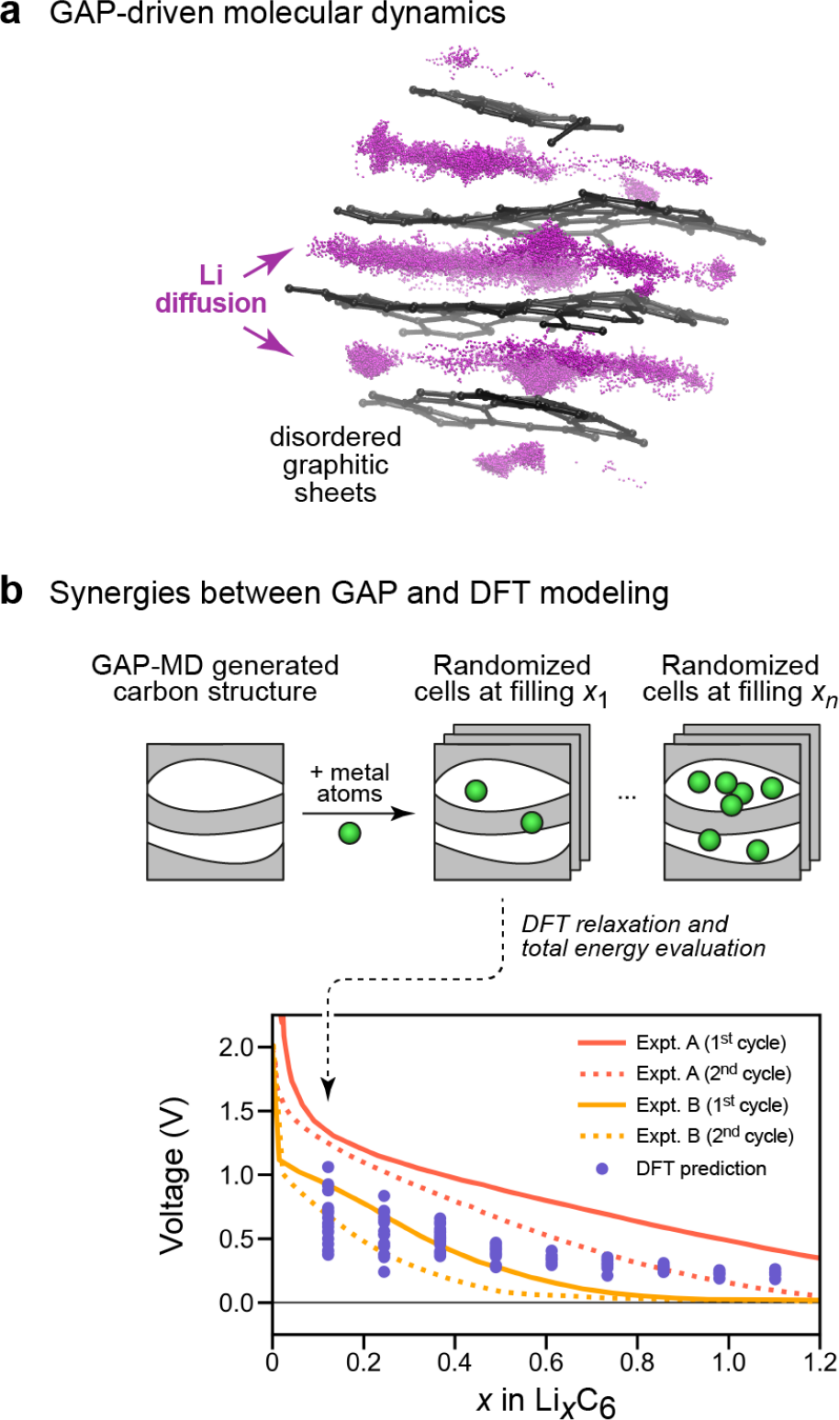
Early examples of how the GAP framework may be used for battery materials modeling. (a) Snapshot from an MD simulation driven by a hierarchical GAP model for Li in carbon structures (ref (119)), extending the pure carbon GAP-17 potential^122^ by adding a difference term; positions of Li atoms are shown in purple; those of carbon atoms are for a single snapshot only. Reprinted from ref (119), with the permission of AIP Publishing. Copyright 2018 AIP Publishing. (b) Synergies between GAP and DFT modeling in studying energetic and electronic effects of alkali-metal intercalation in disordered carbon structures. In this case, disordered and partly graphitized carbon structures were generated in GAP-MD simulations, and several randomized cells with varied Li content (*x*) were used as input for subsequent DFT computations, yielding total energies which may be converted into voltages. Adapted from ref (274). Reproduced by permission of The Royal Society of Chemistry. Copyright 2019 The Royal Society of Chemistry.

Machine-learned force fields are an emerging class of simulation tools in the area of battery materials research,^323−326^ and this has included initial applications of GAP models (Figure 37). A long-term goal of such research would be to compute voltage curves that correspond to the experimental charging and discharging processes. In 2018, it was proposed to use GAP-driven MD to generate relatively small-scale structural models of porous and other disordered carbon structures^273^ which find application in supercapacitors^327^ and battery anodes. The reason for focusing on system sizes of about 200 atoms per cell was the fact that those can then serve as input for DFT analyses, as discussed for ta-C films above. Accordingly, the study in ref (273) included initial DFT computations on the intercalation of Na in “hard” carbon materials, focusing on the evolution of atomic charges with increasing filling, which may be linked to previous operando NMR studies in ref (328). Subsequent work by Huang et al. systematically compared the insertion of Li, Na, and K ions in various disordered carbon structures generated, again, in GAP-driven simulations.^274^

While the previous studies had described the electrode material with GAP and then *subsequently* modeled the metal intercalation using DFT, it is ultimately more desirable to describe the entirety of the system using a machine-learned force field, bypassing the requirement for DFT altogether. Fujikake et al. reported a methodology based on the fitting of energy and force differences, treating the addition of Li to a disordered carbon structure as a “perturbation” of the ideal system (which, in turn, can be fully described by GAP models).^119^ Here we note the development of neural-network potential models for the Li–Si system, which is similarly of large importance for battery anodes.^329,330^ For electrochemically active systems, especially for strongly ionic (e.g., transition-metal oxide cathode) materials, an explicit treatment of the electrostatic interactions may be required. Indeed, ML potential models for systems in which charge transfer is important have been proposed, for example, by Goedecker and colleagues, in the form of charge-equilibration schemes^110^ which were recently incorporated in general “fourth-generation” neural-network potential models.^111,331,332^

Existing GAP-generated structures can be used for new simulations with other methods, which has been exemplified for supercapacitors^333^ and catalysts.^334^ In the first case, pore size effects were studied with empirical-potential simulations that built on existing GAP-based structures; in the second case, a large-scale screening of chemical functionalization was carried out using DFT. Another recent demonstration was the use of existing a-C surface structure models to describe the absorption of biomolecules—seamlessly combined with simpler structure models of graphene or nanotubes.^335^ An even earlier study used the B-GAP fitting database from ref (140) for other types of structural analyses^336^—fully independent from the potential model, but making use of the structural diversity that is explored by GAP-RSS. These examples emphasize the usefulness of openly available databases of *structural* data, which might find use in a variety of future applications.

### 6.6 Molecular Materials

Modeling molecules and materials are fields that often appear distinct, pursued by scientific communities with little overlap, and even the term used for the resulting model is different: “force fields” or potential-energy surfaces for molecules, and “interatomic potentials” for materials. We have briefly touched upon models for isolated molecules in section 5.4, and now we discuss some applications to “molecular materials”. Either liquids or crystals, they consist of strongly bonded molecular units that form extended systems held together by weak interactions, e.g., van der Waals dispersion or dipolar electrostatics. This presents an immediate problem for modeling, because these weak interactions are typically longer-ranged than the covalent interactions, and their length scale of variation is much larger than for covalent bonds. For example, the energy of a covalent bond has significant variation when the bond is stretched or compressed by a distance on the order of 0.1 Å; in contrast, intermolecular electrostatic and dispersion interactions vary significantly only on the length scale of 1 Å or greater.

There are essentially two approaches: the first is to use a *molecular body order expansion* in which the total energy is split up into contributions of each isolated monomer, the interaction energy of each dimer, each trimer, and so on. In this case, each of these terms corresponds to just isolated molecules or small clusters of molecules, and we are back to that modeling problem. Alternatively, we can consider the entire loosely bound collection of molecules as an *extended material*. In this case, we can use exactly the same descriptors and fitting methodology as for material systems. The input data also need to be similar: electronic-structure total energy calculations in the condensed phase, almost invariably using periodic boundary conditions. In practice, this limits us to using DFT, rather than the more accurate correlated wavefunction theory that one would be able to use for isolated molecules and clusters.

Neural networks for water^170,185,337,338^ were among the first models of a molecular material that did not explicitly rely on prescribing the fixed topology of the molecules. Water is somewhat of a special case, where the “weak” intermolecular interactions are relatively strong hydrogen bonds, and so this “material treatment” can be expected to be more successful. The great flexibility of the neural network functional form helps to simultaneously describe the short-range covalent bonds and the intermolecular interactions. Using GPR would be more difficult, because one of the key ingredients of those models is an optimized length scale for the kernel function that generates the basis. One way to deal with these different interactions is to focus the ML effort on the short-range part and to describe the long-range interactions using an analytical *baseline* model, as detailed in section 4. This has been done using a long-range pair potential for dispersion interactions for a phosphorus model that included the low-density molecular liquid phase^163^ and earlier for carbon to deal with the weak dispersion that holds layers of graphite together.^152^ Another possibility is to build models that describe the long-range nature of the interactions at the level of descriptors, such as models based on long-distance equivariants^339^ (LODE) that combine atomic neighborhood density features similar to SOAP with an artificial “far field atom-density potential” that captures long-range interactions.^340^

Another example of a purely “material-type” model is the application to the prototypical hybrid perovskite, methylammonium lead iodide, which we discussed in connection with active learning (cf. Figure 13).^123^ The methylammonium cation did not need to be defined as a separate topological unit, nor did its connectivity need to be fixed. All that enters the fit is a collection of atomic coordinates and associated energies, forces, and virial stresses. It may be expected that other hybrid materials, containing perhaps very complex organic molecules, will provide rewarding targets for investigations with similar machine-learned potentials; similar work was done (using a neural-network model) for one of the prototypical metal–organic frameworks, MOF-5.^282^

One very effective way to use GPR for describing molecular materials with high accuracy is to combine the above-mentioned two approaches as follows. The total energy is separated into intramolecular (“monomer”) and intermolecular terms as usual, but the intermolecular part (the “interaction energy”) is not treated by a further molecular body order expansion but rather by the material model framework using SOAP as atom-centered descriptors and GAP for regression. This solves the problem of disparate length scales.

**Figure 38:**
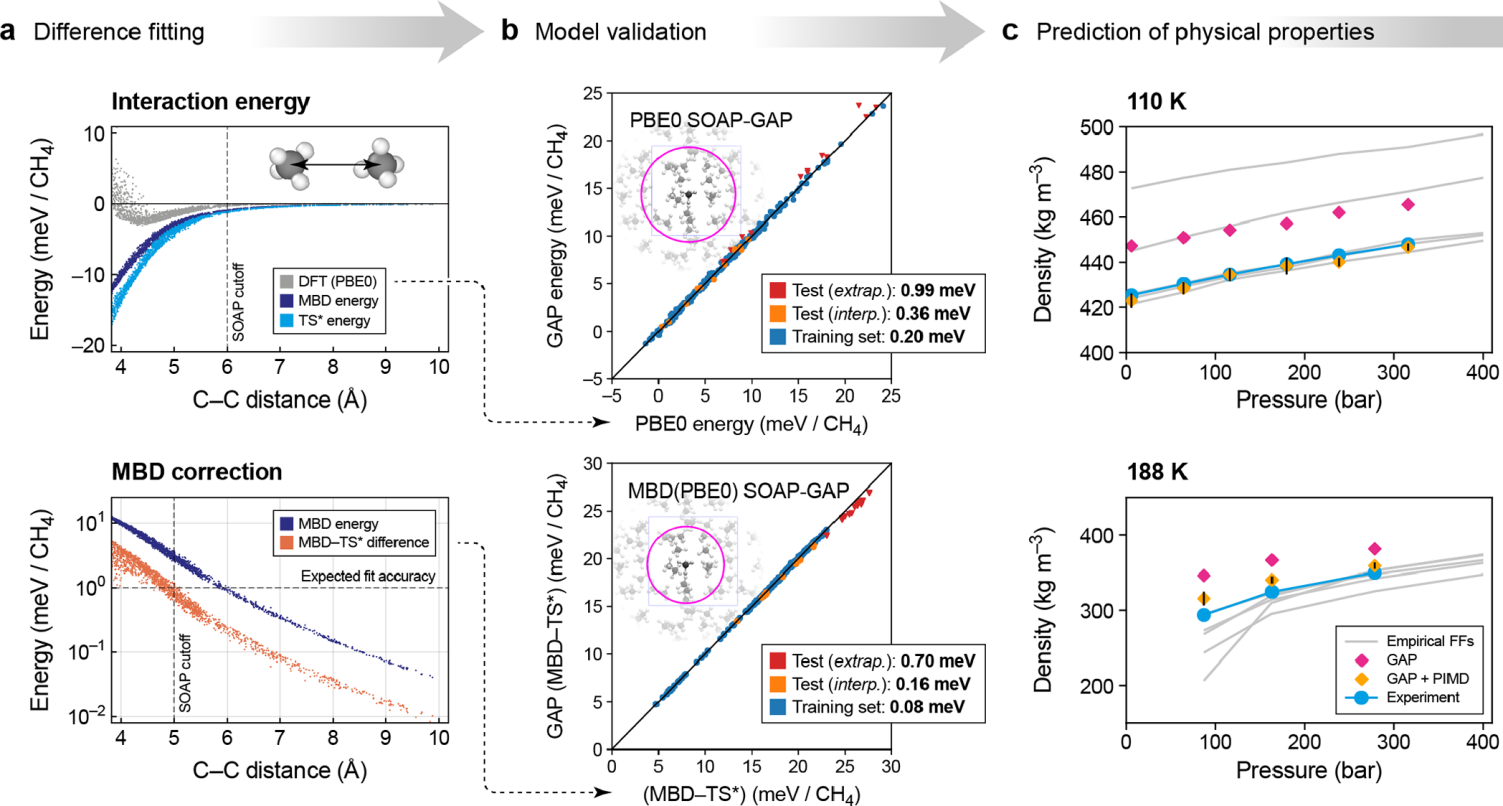
Hierarchical GAP model for fluid methane (CH_4_). (a) Different terms in the interaction energy of a pair of methane molecules (geometries obtained from the condensed phase), with the top panel showing the PBE0 and MBD energy as well as the TS correction using a fixed Hirshfeld volume (denoted TS*). The bottom panel shows the MBD energy on a logarithmic scale as well as the difference between MBD and TS*. (b) Two SOAP-GAP models are fitted separately to reproduce the PBE0 interaction energy and the MBD-TS* difference. The panels show the validation of the interaction models against their respective references. Numerical errors are given in the legends. (c) Prediction of physical properties, here shown for the density–pressure isotherms, comparing the performance of the GAP (magenta, without; yellow, with path-integral MD to model quantum nuclear effects) with experimental data (cyan); the results of various empirical force fields are indicated by gray lines; the black bars indicate the size of statistical error. Details are given in ref (172). Adapted with permission from ref (172). Copyright 2019 American Chemical Society.

We present two examples of this approach, both using hierarchical modeling in several different ways. In the first one, the total energy of fluid methane was broken up into the following terms:^172^
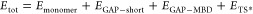
83with the last three constituting the intermolecular terms. The monomer term was a simple force field with two- and three-body terms fitted to CCSD(T) data on isolated methane molecules. The “GAP-short” term represents the short-range part of the interaction energy and was fitted to interaction energies computed using DFT-PBE0^341^—and since there is no dispersion in this DFT method, the resulting interaction is mostly repulsive and can be adequately captured with a finite-range potential. The last two terms together account for dispersion. Of these, “TS*” is a pair potential along the lines of the Tkatchenko–Scheffler^342^ scheme using a fixed C6 coefficient (obtained by averaging it over methane conformations), and the “GAP-MBD” term is the difference between the many-body dispersion energy^343^ and the TS* baseline. Having subtracted TS*, the remainder of the MBD energy could be described by a SOAP-GAP model with 5 Å cutoff with an error of less than 1 meV per molecule. Both GAP models used condensed-phase data with 27 methane molecules in the unit cell. Figure 38 shows the validation of the two separate intermolecular fits (a–b) and the successful prediction of the density as a function of temperature and pressure (c), once path integral molecular dynamics (PIMD)^344^ was applied to the combined potential. Note that applying PIMD results in up to a 10% shift in the predicted mass density. The gray lines in Figure 38c represent results from a variety of empirical force fields, some of which have been directly parametrized to reproduce the density—so they do this successfully, *but not for the right reason*, in the sense that they do not represent the correct potential-energy surface.

**Figure 39:**
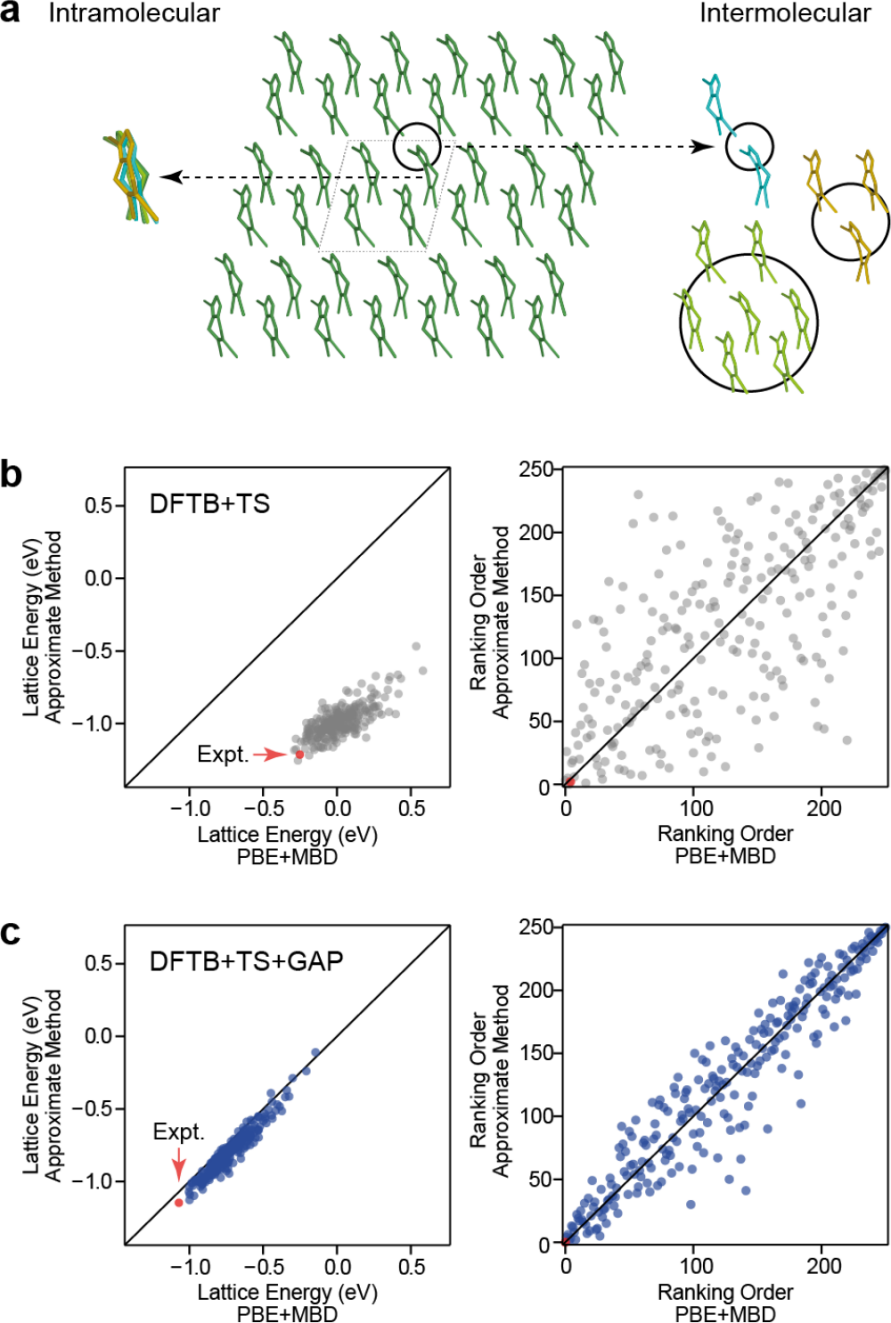
GAP models for molecular crystal-structure prediction. (a) Illustration of the hierarchical construction of the ML model. Intramolecular and intermolecular terms are fitted independently, and both are difference models with DFT at the PBE+MBD level being the higher level of theory and the semiempirical DFTB+TS serving as the lower level baseline (cf. Figure 18a). The database of the intramolecular model consisted of isolated molecules, whereas that of the intermolecular model contained small clusters obtained from DFTB relaxations of crystals. The molecule shown is tricyano-1,4-dithiino[c]-isothiazole, which was target XXII in the sixth blind test of organic crystal-structure prediction.^346^ Results of independent crystal structure searches performed with the DFTB+TS baseline (panel b) and the DFTB+TS+GAP model (panel c) on lattice energies (left) and rank order (right), with respect to the PBE+MBD reference (computed without further relaxation). The red dot indicates the experimentally found crystal structure. The large overall shift in the DFTB+TS energies is due to the incorrect monomer geometry of the baseline model. Reprinted from ref (175); original figures published under the CC BY-NC 3.0 license (https://creativecommons.org/licenses/by-nc/3.0/).

The second example is molecular crystal-structure prediction,^175^ using a double-hierarchical model in which both the molecular monomers and their interaction energy are described, separately, by respective SOAP-GAP fits—each using the semiempirical DFTB model^345^ with the TS correction^342^ as baseline model and DFT+MBD^343^ as its ultimate target. The intramolecular GAP model is fitted to the following difference,
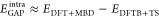
84and is trained on isolated molecules with geometries obtained from the crystal, illustrated in Figure 39a. The GAP interaction energy is fitted to the difference of differences,
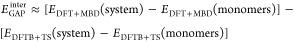
85and is trained on clusters (referred to as “X-mers” in the original paper), again carved from crystal configurations obtained by simulations using the baseline model. The GAP corrections were shown to significantly improve on the energy prediction of the baseline model both in an absolute sense and in ranking low-energy crystal polymorphs (Figure 39b and c), with savings in computational cost of over a factor of 300 for a full crystal-structure search. Note that the computational cost of evaluating the combined model was shown to be dominated by the semiempirical baseline, with the GAP model taking less than 2% of the total time.^175^

When modeling molecular materials nowadays, one is almost always looking out for ways to go beyond standard DFT. Since this is now routine for the ML modeling of isolated molecules (see, e.g., ref (180)), even considering excited states,^347−349^ it is natural to use the techniques illustrated here to carry over this high level of accuracy to periodic systems. Reference electronic-structure methods and training databases have to be chosen carefully, but it is now within reach to train intermolecular potentials using symmetry adapted perturbation theory,^350^ random phase approximation,^164^ or even quantum Monte Carlo^351^ data.

## 7 Applications (II): Beyond Force Fields

Even though the bulk of this review focuses on the central problem of fitting interatomic potentials, GPR is applicable to a wide range of atomic-scale properties. Early work on molecular ML used descriptors for the molecular structure such as Coulomb matrices—in combination with GPR/KRR—to model a couple dozen properties of small organic molecules.^232,235,352,353^ In a systematic comparison including different descriptors and regression techniques, kernel methods were shown to match, and often outperform, nonlinear techniques including graph convolutional networks^354^ (see also section 5.4).

Several of the efforts aimed at learning properties other than the potential energy link back to the problem of constructing force fields. This is the case, for example, for the prediction of atomic charges^355,356^ and molecular multipoles^81,357,358^ that are then used to define an electrostatic baseline to model long-range interactions. Other examples are the direct prediction of the lattice energy of molecular crystals, using as training and as inputs only the geometries optimized with an empirical force field^359^ (which is a simpler learning task than training a fully general potential for the same class of systems), and the estimation of free-energy surfaces,^360^ which involve finite-temperature sampling with a (traditional or machine-learning) force field. Although the present review focuses on fully atomistic models, the construction of ML-based coarse-grained force fields is a burgeoning research field where initial progress has been made with GPR-based and other ML methods.^361−365^

It is also possible to use GPR for Bayesian optimization (BO),^366^ which attempts to find the *global* PES minimum by using the predicted value and predicted variance to optimize the choice of sampling points. ML schemes have been applied to accelerate the search of the most stable configurations^367,368^ and of saddle-point structures associated with an activated transition.^369−372^ In this case, a GP model of a PES is iteratively generated, but it is generally considered to be only an aid for finding the global minimum. This approach has been used for finding minimum-energy crystal structures^373,374^ by iteratively proposing structures that maximize the likely energy gain, relaxing them with DFT, and adding the resulting structures and energies to the fitting database for the PES model of the next iteration. After the first iteration, the potential is only fit to DFT local minima. BO approaches have been used for ionic diffusion paths:^375^ for this application, the PES was expressed as a function of the position of a single diffusing atom; the fitted energies were computed after relaxing all other atomic positions with DFT, and BO was used to simultaneously optimize the positions of the migration path end points (local minima) and the energy barriers along the path (saddle points). GPR and the predicted uncertainty were used in constructing a surrogate model for nudged-elastic-band computations.^376^ Hammer and co-workers combined structure optimization with a GPR model^368^ and showed how their atomistic structure learning (ASLA) technique^377^ can be coupled with a GPR-based potential model fit to accelerate the global search for stable structures.^378^

In the remainder of this section, we provide an overview of several applications of GPR to properties that are different from potential-energy surfaces. We have selected these applications to highlight how atomistic ML based on local representations can provide surrogate models for any atomic-scale property that can be computed by electronic-structure methods—including atom-centered scalar properties (NMR chemical shieldings), tensorial properties (dipole moments and polarizabilities), scalar fields (the electron density), and energy-dependent properties (the density of states). The main take-home message is that even though the overall GPR scheme presented in the previous sections is general enough to underpin ML predictions of arbitrary properties, their specific nature requires careful consideration of the structure of the model, which needs to mirror the symmetry properties and physical behavior of the target.

### 7.1 NMR Chemical Shieldings

The vast majority of the models we discuss in this review rely on an atom-centered decomposition of the target property. As a consequence, they can be applied in the most straightforward way to the prediction of properties that are inherently atom-centered,^379^ such as the chemical shieldings of nuclei that determines the characteristic signature of a material or a molecule in nuclear magnetic resonance experiments. NMR measurements usually determine chemical *shifts*, i.e. differences between the NMR shieldings of the sample and of a reference. Even though they are exquisitely sensitive probes of the chemical environment of nuclei, the small changes in shieldings/shifts that are necessary e.g. to distinguish different polymorphs of the same molecular crystal cannot be interpreted on a qualitative level, and theoretical predictions are invaluable to assist the analysis of experiments. Models for the prediction of chemical shieldings in solution^380^ and for polypeptides^381,382^ have been among the first applications of artificial neural networks in chemistry. With the development of frameworks to compute the magnetic shieldings of nuclei using DFT,^383,384^ such as the gauge including projector augmented wave (GIPAW) method, it has become possible to construct regression models that are based on a first-principles computational framework.

**Figure 40:**
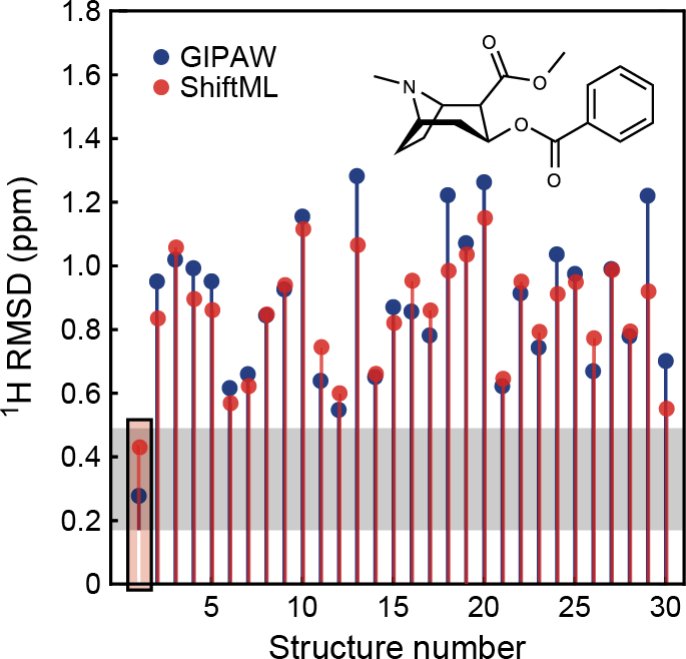
NMR chemical shift prediction. The figure shows a comparison between the ^1^H chemical shifts for a set of hypothetical polymorph structures of cocaine, obtained using a crystal structure prediction algorithm, and those of the most stable polymorph, determined experimentally. For each candidate structure an aggregate RMSD is shown between experimentally measured shifts and shifts calculated using either GIPAW-DFT (blue) or ShiftML (red). The gray zone represents the confidence intervals of the GIPAW-DFT ^1^H chemical shift RMSD. Candidates that have RMSDs within this range would be determined as correct crystal structures using a chemical shift-driven solid-state NMR crystallography protocol. Adapted from ref (386). Original figure published under the CC BY 4.0 license (https://creativecommons.org/licenses/by/4.0/).

The fact that electronic-structure calculations provide chemical shieldings for a specific nuclear configuration, rather than the average over molecular fluctuations that is probed experimentally, makes a DFT-based ML model particularly useful for solid-state NMR, in which fluctuations around equilibrium configurations are less pronounced. Early models based on a neural network for ^17^O and ^29^Si shieldings in silica^385^ were recently complemented by a framework enabling predictions for ^1^H, ^13^C, ^15^N, and ^17^O in molecular crystals,^386^ which relies on a GPR model using SOAP kernels that incorporates many of the techniques discussed in the present review, including multiscale kernels and sparse models. This “ShiftML” model^387^ achieves an accuracy comparable to the reference DFT calculations and can be combined with experimentally determined shifts to assign the crystal structure of a sample to the most compatible polymorph among a set of candidates (Figure 40). In combination with model error estimation, it is also possible to establish, in a quantitative manner, the reliability of such assignment^388^ and to use the ML prediction to interpret solid-state NMR experiments.^389^

### 7.2 Dielectric Response Properties

The response of the energy of a system, *U*, to an applied electric field, **E**, gives rise to a hierarchy of dielectric response properties, *∂*^*n*^*U*/∂ **E**^*n*^—these include the polarization, **μ**, the polarizability, **α**, and higher-order responses such as the hyperpolarizability, **β**. These quantities are inherently tensorial, and so they require regression models that incorporate the covariance of the tensor with respect to rigid rotations of the system. As anticipated in section 3.4, many early attempts to build regressors for dielectric properties, as well as multipole moments, relied on the definition of a local reference frame attached to the molecular building blocks.^81,83^ Another approach, reflecting well-established practices in the construction of molecular dipole moment surfaces,^390,391^ involves associating formal charges to each atom and combining them with the atomic positions to compute a formal polarization vector—this approach is also readily applicable to neural-network models.^84,238^ Fully symmetry-adapted models, instead, define a kernel or feature basis that reflects the covariant properties of the target. Early proponents of the application of covariant models to the prediction of tensorial properties relied on the kernel framework^80,91^ described in section 3.4, as well as on an alternative formulation that uses formal atomic charges to determine an atom-centered reference frame—which can be elegantly expressed in terms of an “operator ML” formalism.^82^

**Figure 41:**
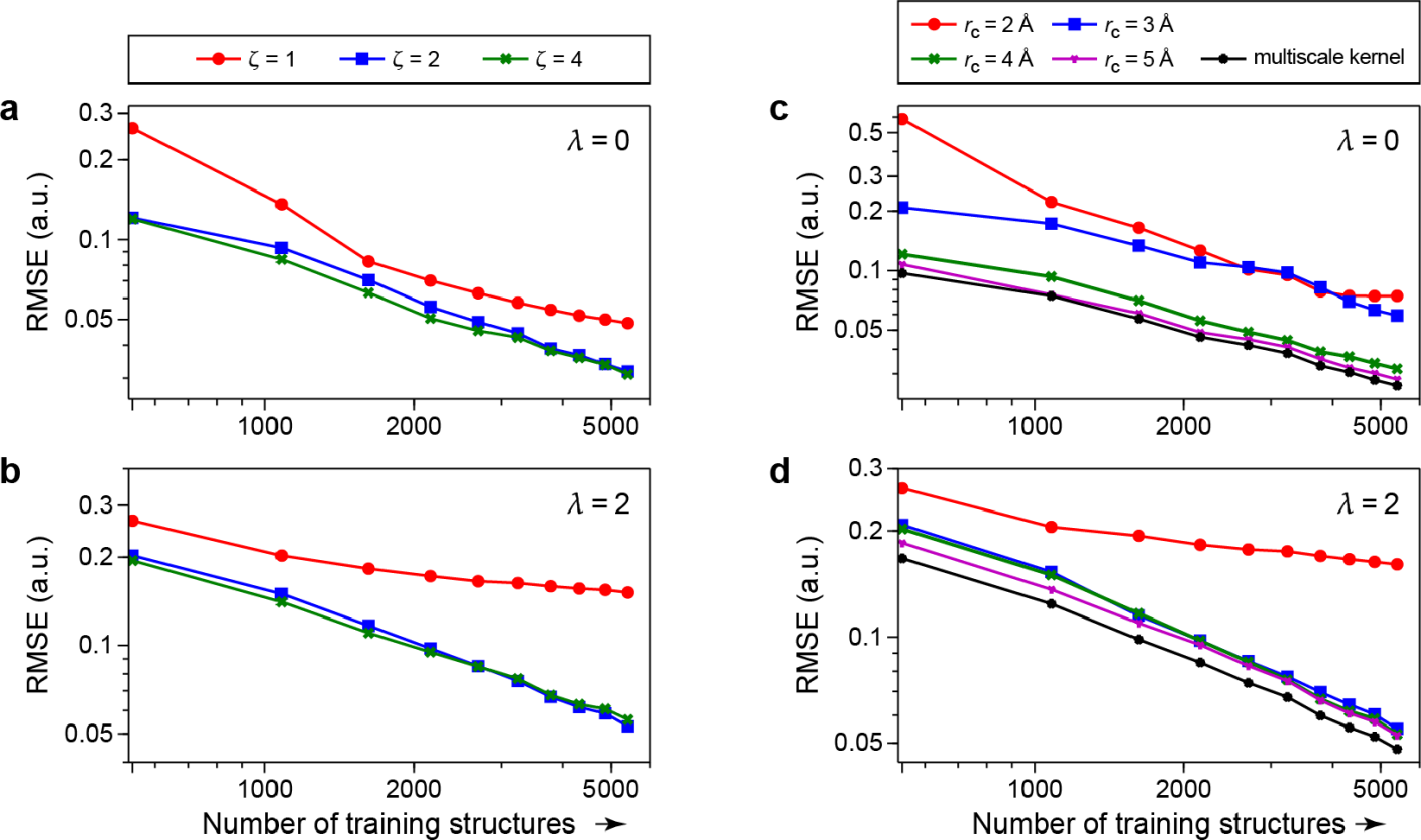
Learning tensorial properties. The figure shows learning curves for the λ = 0 (top) and λ = 2 (bottom) components of the per-atom polarizability for the QM7b dataset.^352^ Polarizabilities were calculated using CCSD, and the test set in all cases consists of 1811 molecules. (a, b) Effect of the kernel exponent. Nonlinear (ζ = 2, 4) SA-GPR SOAP kernels yield much better asymptotic learning performance than the linear (ζ = 1) form. A radial cutoff of *r*_*c*_ = 4 Å is used throughout. (c, d) Effect of the environment cutoff radius. Polarizability is a property that depends strongly on long-range correlations, and so a large cutoff distance is usually beneficial. However, a multiscale kernel built by combining kernels with different cutoffs, with weights that are optimized by cross-validation, yields a small but consistent improvement over each of the individual models. Adapted from ref (99).

The earliest applications of the SA-GPR approach on which we focus in this review tackled the problem of modeling the dielectric response series of water oligomers up to the third order, and the electronic dielectric constant of bulk water.^91^ These benchmarks highlighted the success of SA-GPR across a range of orders of tensor and its ability to handle systems that cannot be split into well-defined molecular units. However, it is with the AlphaML model of molecular polarizability^99,393^ of organic molecules that SA-GPR proved its ability to achieve an accuracy at least as good as that of DFT. This accuracy was achieved for both the scalar and the tensorial part of **α** across large swathes of chemical space, even when extrapolating to much larger and more complex models than those included in the training set—a reflection of the transferability afforded by atom-centered decomposition of the targets. The fact that SA-GPR is an extension of scalar GPR means that developments designed originally for scalar learning can be transferred straightforwardly to tensor learning; for example, the use of multiscale kernels that combine several length scales with optimized weights improves the model performance over individual models (Figure 41, right panel). The standard result that [*k*(*A*, *A*′)]^ζ^ is also a valid invariant kernel, used to introduce nonlinearity in SOAP GPR models, cannot be used directly to predict covariant properties, since a spherical harmonic raised to a power greater than one is a sum of spherical harmonics of different orders. One simple way to incorporate nonlinear models in SA-GPR involves using products of covariant and invariant kernels,
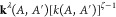
86which is still a covariant kernel of order λ. In the case of polarizability learning, this combination of spherical and scalar kernels was found to improve the performance of the models by a factor of 2–3 (Figure 41, left panel).

**Figure 42:**
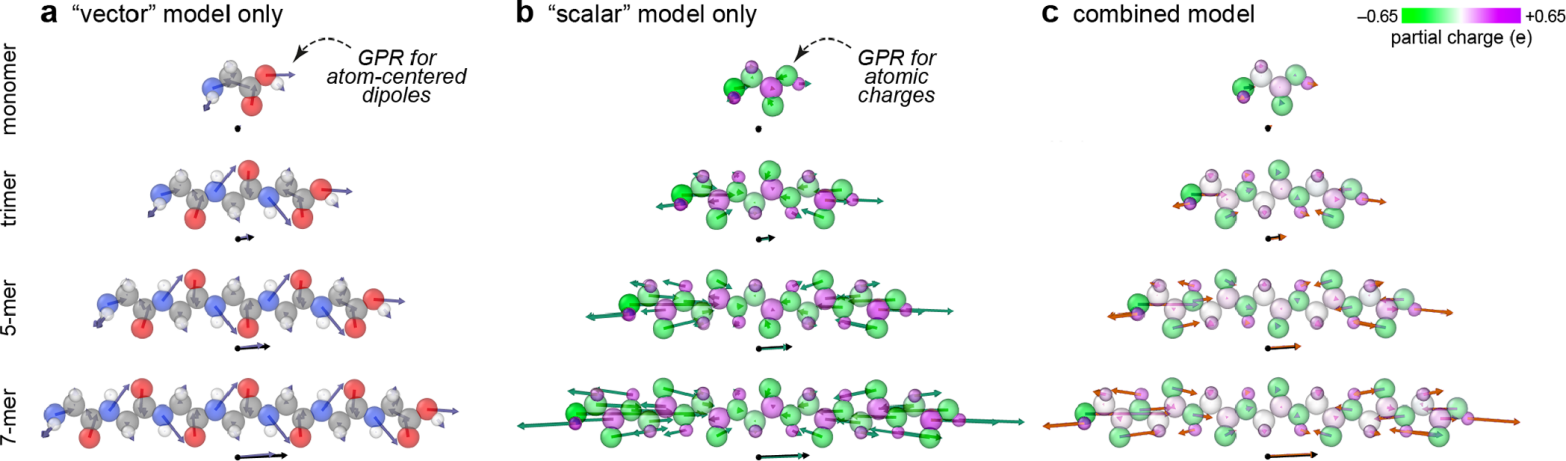
Learning dipole moments. The figure shows atom-centered contributions to the dipole moment of different polyglycine molecules from the monomer to the 7-mer. (a) Results of a “vector” (λ = 1) SA-GPR model in which the predicted dipole is made up of atom-centered dipoles (gray vectors). (b) Results of a scalar (λ = 0) GPR model, where atom-centered charges (whose magnitude is indicated by the green/purple color scale) are predicted instead and used to calculate the molecular dipole moment. For this model, the green vectors show the predicted charges multiplied by the atomic displacements. (c) Results of a model in which scalar and vector SA-GPR are combined, and the prediction is a combination of atom-centered dipoles and charges (red vectors give the weighted sum of the two contributions). Below each molecule, the black vector gives the molecular dipole moment calculated using the reference electronic-structure method (B3LYP-DFT), and the gray, green, or red vector gives the total GPR prediction. Adapted from ref (392).

The prediction of molecular dipole moments is particularly interesting. Being the simplest possible dielectric response, and the simplest nontrivial multipole, dipoles have been used as benchmarks for many different methods, including “operator ML”,^82^ learning the position of Wannier centers,^394^ as well as learning of atomic charges.^84,238,390,391^ As discussed in ref (392), different approaches can be linked to a different physical model of the origin of the polarization. A λ-SOAP model describes a combination of atom-centered dipoles, which is most effective to describe local polarization effects, while a model based on atomic charges is more suitable to describe the presence of charged groups or long-range charge transfer. Figure 42 illustrates predictions of molecular dipole moments at the B3LYP-DFT level that combine a λ-SOAP model for atom-centered dipoles and a scalar SOAP model for atomic partial charges. In general, the combination of the two models gives predictions that are better than either model by itself. In particular, a substantial contribution from the scalar part of the model improves significantly the transferability of this “MuML” model, which is trained on small organic compounds from the QM7b dataset,^352^ to larger molecules and to compounds with substantial charge transfer. This more flexible model achieves an error for the out-of-sample predictions on the QM9 dataset^232^ that is smaller than that of an “operator ML” model trained on the larger molecules. A comparable “in-sample” MuML model reduces the error further by a factor of 3.

In combination with ML potentials, the possibility of computing the dielectric response of molecules and condensed-phase systems makes it possible to inexpensively evaluate spectroscopic observables. For instance, the infrared (IR) spectrum can be obtained by Fourier-transforming the dipole moment correlation function, ⟨**μ**(*t*)**μ**(0)⟩, along an MD trajectory;^395^ a similar expression for the polarizability, **α**, yields the Raman spectrum, and a combination of the two can be used to calculate the sum-frequency generation (SFG) spectrum. The theoretical calculation of light scattering also requires tensor properties; for example, second-harmonic scattering (SHS) is determined by the first hyperpolarizability tensor, **β**.^83,396^ This strategy has been applied to the IR and Raman spectra of molecules^84^ and condensed phases^86,203,394^ and even to incorporate the effects of the quantum-mechanical behavior of light nuclei on the spectroscopic properties of complex molecules and condensed phases^85,397^—a remarkable feat that would have been all but impossible without ML models that are capable to accurately reproduce *all* of the properties that are accessible to electronic-structure calculations.

### 7.3 Electron Density

The electron density, ρ̃(**r**), of a molecule or material provides all of its ground-state properties in principle, and as such it presents a natural target for ML models. Many techniques have been proposed in recent years, differing not only by the structural representation or the regression algorithm but also by the way the density is discretized. Early efforts, most notably the foundational work of ref (399), used the coefficients of an expansion in plane waves. Being global, and dependent on translations and rotations of the atoms, this approach suffers from poor transferability. Another method, first introduced in ref (400), is based on the separate prediction of the density at each point, **r**, in terms of a description of an atomic environment centered at **r**. This is usually combined with neural-network models^401−403^ that must allow for very fast training and estimation, because for each configuration millions of grid points have to be individually learned and predicted.

An alternative approach, which combines the transferability of a local model with a relatively small number of prediction targets, relies on a decomposition of the total electron density into atom-centered terms of the form
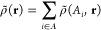
87where each of these atom-centered terms is given by
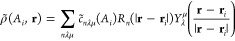
88using a basis of radial functions *R*_*n*_(*r*) and spherical harmonic  and emphasizing that the expansion coefficients are taken to be a function of the local atomic environment *A*_*i*_. Although for simplicity we do not indicate it here, the basis functions and the coefficients usually depend on the chemical nature of the central atom. For each value of *n* and λ, the _*nλμ*_(*A*_*i*_) transform as spherical harmonics, making them amenable to learning by SA-GPR.^404^ One subtlety, which can be readily resolved in the case of GPR models, involves the nonorthogonality of basis functions centered on different atoms. The density expansion coefficients **c̃** cannot be computed directly by projecting the density on the basis functions. Such a projection, instead, yields a set of weights,
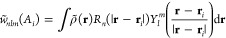
89that are related to the expansion coefficients by **Sc̃** = **w̃**, where **S** indicates the overlap matrix between basis functions. However, it was found that—because the overlap matrix is often ill-conditioned—determining the coefficients and learning them independently leads to inaccurate models; instead, one has to build a GPR framework in which the entire decomposition is learned at once.

The loss function to be minimized, *L*(*A*), for each training structure, *A*, is given by
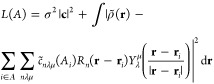
90that depends on the SA-GPR coefficients through
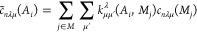
91where *M* indicates a set of representative environments (that could in principle be different depending on the nature of the central atom and the density basis function associated with the coefficient).

**Figure 43:**
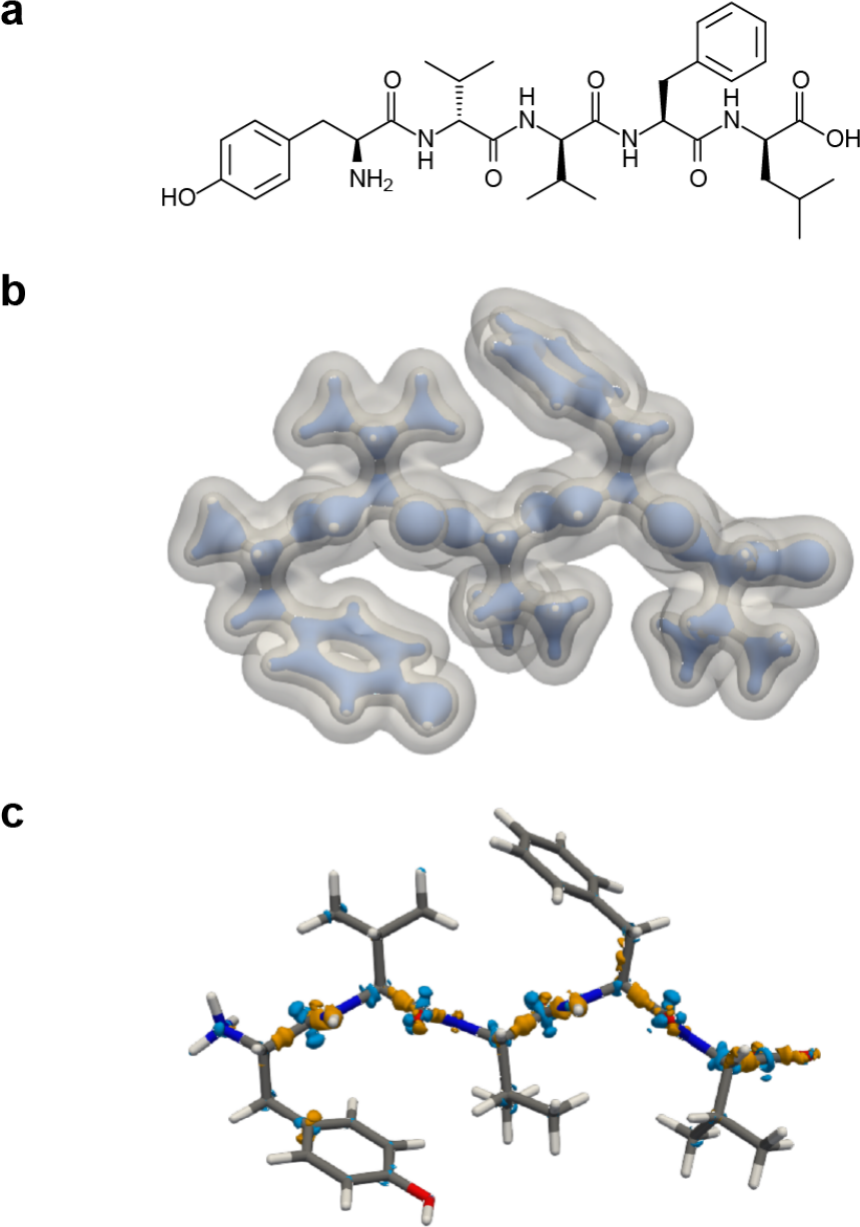
GPR model for the electron density. (a) Chemical structure of the enkephalin pentapeptide studied here. (b) Electron density prediction for the same molecule, using a model trained on dipeptides. Three isosurfaces of the predicted density are shown (0.5, 0.1, and 0.001 electrons bohr^–3^). (c) Difference between the predicted and calculated electron densities (showing isosurfaces of ±0.01 electrons bohr^–3^, with positive deviations in yellow and negative deviations in blue). Figure adapted from ref (398). Original figure published under the CC BY-NC 3.0 license (https://creativecommons.org/licenses/by-nc/3.0/).

The final expression for the regression weights,
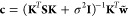
92shows how the nonorthogonal nature of the targets leads to the coupling of kernel blocks that are associated with different centers and basis functions. By learning the entire decomposition at once, it was possible to predict the electron density for a set of hydrocarbons with the minimum error possible given the decomposition.^404^ The models are transferable due to the local nature of the decomposition and are straightforwardly extrapolated to larger molecules. In fact, the accuracy of the local density expansion plays a crucial role in determining the prediction accuracy for ρ̃(**r**), which was addressed in subsequent work^398,405^ by using resolution of the identity basis sets.^406^Figure 43 shows the accuracy that can be obtained for an enkephalin molecule using a model trained only on dimers of small organic fragments.^398^ The error is concentrated on the oligopeptide backbone, a chemical motif that is not present in the training set. The availability of an accurate, transferable prediction of the electron charge density opens up the way to obtain ML models of similar quantities, such as the on-top density^407^ or the local spin density.

### 7.4 Density of States

The electronic density of states (DOS) is a fundamental fingerprint of the electronic structure of a material, and DOS plots derived from DFT computations are found in countless publications and probed for chemical insight.^408^ We discuss here a recently developed approach to machine learning aspects of the electronic DOS for atomistic systems using GPR. This is motivated in two ways: first, if successful, it would allow for an inexpensive prediction of the electronic DOS for much larger systems that are accessible to direct DFT evaluation; second, it would allow one to compute derived properties, such as the band width.

Once an electronic-structure computation for a given atomistic system has been carried out, the DOS is conventionally obtained as
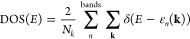
93where the sum runs over the bands and the **k**-point sampling of the Brillouin zone and *ε*_*n*_(**k**) are the single-particle eigenvalues of the electronic Hamiltonian.

**Figure 44:**
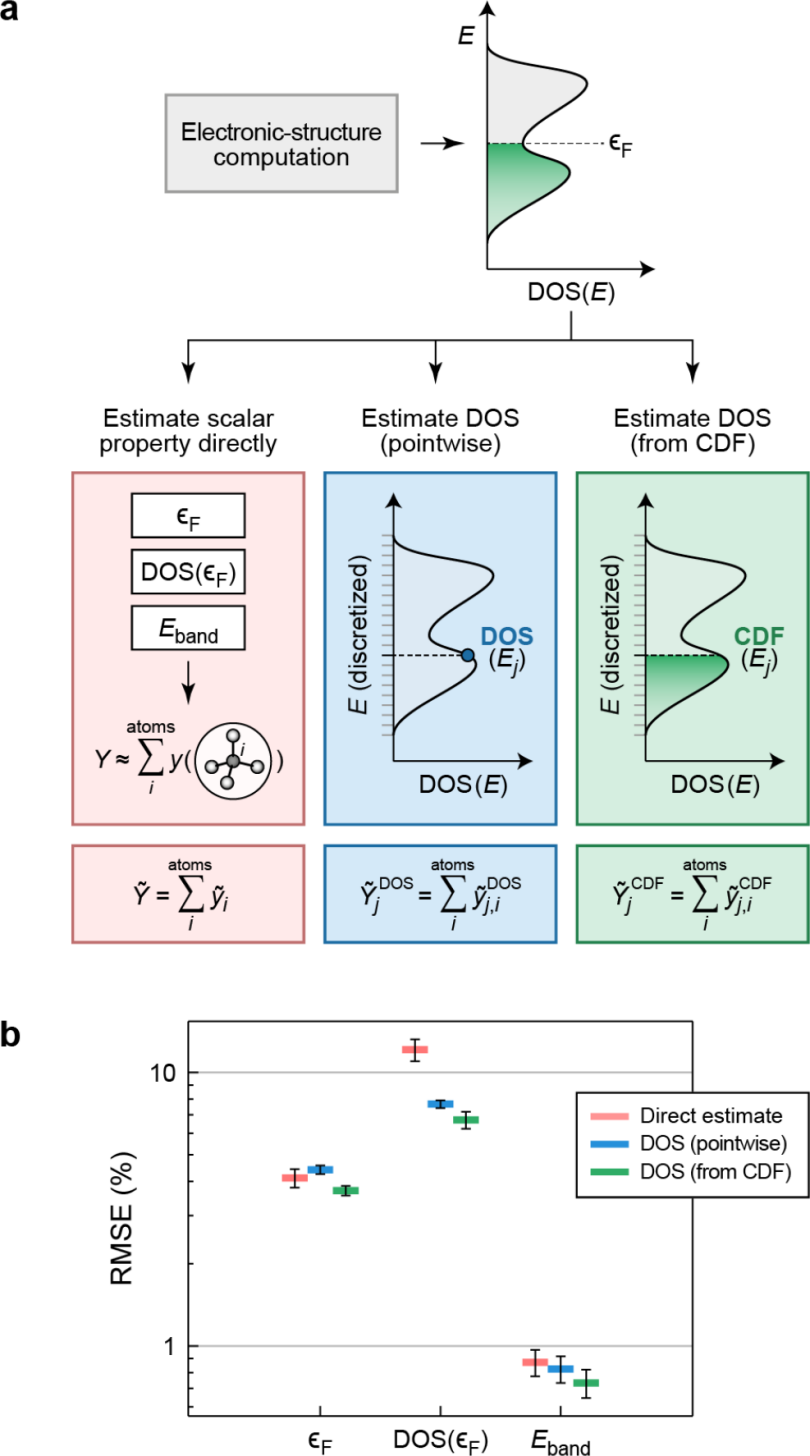
(a) Machine learning the electronic density of states (DOS) in the framework of GPR. Three approaches for estimating properties *Y* are shown in a highly schematic way: these properties could be scalar properties derived from the DOS (red), values of the DOS itself on a discrete grid of energy values (blue), or values of the cumulative distribution function (CDF; green); see text for details. (b) Average errors for the prediction of quantities that can be computed from the electronic DOS in amorphous silicon,^212^ either directly (red) or using two different representations of the DOS curves: the pointwise approach (blue) and that obtained by differentiating the CDF (green). The error bars represent the standard error of the mean. Errors are expressed as percentage of the intrinsic variability within the dataset. Drawn with data from ref (212).

Following the notation of section 2, we denote a global property by the capital letter, *Y*, and the approximation of this property by the GPR model by *Ỹ*. In line with the general linear structure of GPR models discussed earlier, a transferable prediction of properties of the entire stucture can be obtained in terms of a sum of local, atom-centered contributions,  viz.
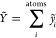
94where the sum runs over all atoms in the structure, and each local term is a GPR model that depends on a representation of the environment (e.g., based on SOAP). Figure 44a shows this generic construction in the red panel, for directly regressing different quantities that are all derived from the DOS: the Fermi energy *ε*_*F*_, the DOS value at the Fermi level DOS(*ϵ*_*F*_), and the “band energy”, *E*_band_ (by integration over the filled bands, not to be confused with the individual eigenvalues, *ϵ*_*n*_(**k**)).

Furthermore, one can fit models for the DOS itself. This is useful because one may be interested in predicting the DOS and comparing it to experimental observations and also because predictions for several derived quantities can be made from the predicted DOS. Naively, one might represent the (continuous) DOS by discretizing the energy into a grid of values *E*_*j*_, with *j* being a running index and the step size denoted *δE*, and model the DOS value at each energy level independently,
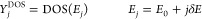
95Just as before, we model each global quantity as a sum of local atomic contributions,
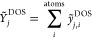
96Alternatively, instead of modeling the DOS, one can work with the integrated DOS (IDOS) up to a given energy value, *E*_*j*_,
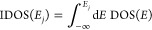
97The discretized representation of the IDOS, to which we refer here as the cumulative distribution function (CDF), is
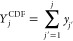
98which again can be fitted as a sum over local contributions,
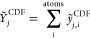
99and it was found in ref (212), and is shown in Figure 44b, that fitting the CDF consistently improves the accuracy of its property predictions over learning the DOS itself (and also over fitting properties directly). This improvement can be understood in terms of the link between the Euclidean distance between CDFs and the Wasserstein distance between the underlying distributions^409^—the latter being a better notion of similarity between spectra that often differ by small shifts in the positions of sharp peaks. Once the CDF is known, differentiation yields the DOS. Note that the kind of local model in ref (212) differs from previous work that instead learned simultaneously the charge density and the DOS using a regular 3D grid of points extending throughout the simulation box.^401^

The fact the fitting the DOS is better than fitting derived properties directly is a consequence of the locality properties of the DOS (in fact, the *local* density of states is computed regularly by many electronic-structure packages by projecting electronic states onto atom-centered basis functions). In constrast, the Fermi energy is determined by a global charge neutrality constraint that depends on the overall distribution of energy levels throughout the sample. These observations highlight the interplay between the physical nature of the target quantities and the structure of the regression model.

Work on learning densities of states is at an early stage, but efforts are also underway using different methodologies including neural networks,^410−413^ with ref (411) also employing atom-centered descriptors, and KRR.^414^ We note that if the structures in the database (for which the DOS computation with the reference method is carried out) are sufficiently small, the reference DOS can be evaluated with more accurate and computationally expensive methods—specifically, hybrid DFT, which was recently demonstrated for silicon;^164^ the latter work will be discussed below. In all these cases, an accurate and inexpensive ML model of the DOS provides a simple yet useful probe into the electronic properties of materials simulated by ML potentials—allowing one, for example, to estimate the role played by electronic excitations on the thermophysical properties of materials or to perform simulations that directly incorporate the role of finite electronic temperature.^415,416^

## 8 Conclusions and Outlook

Machine learning methods have arrived in theoretical and computational chemistry, and they are here to stay. In the present work, we have reviewed Gaussian process regression (GPR), one of the approaches to “learning” (fitting, regressing) atomic properties—scalars, vectors, and higher-order tensors. The applications of GPR are diverse, ranging from the prediction of local atomic properties such as NMR chemical shifts and dipole moments to the construction of accurate interatomic potentials, or force fields, e.g. in the Gaussian approximation potential (GAP) framework. Having been considered a highly specialized technique that requires expert knowledge until recently, ML methods are now poised to achieve more widespread use in chemistry. Methodological developments and extensive tests for numerical accuracy have been done, and there is no doubt that further optimizations are possible and important. Furthermore, it is also timely to implement protocols in a widely accessible fashion, enabling researchers to apply these methods to answer pressing scientific questions. We summarize how we see the field at present and where we envision it developing in the coming years.

The title of the present work refers to “materials and molecules”, and this wording reflects a separation that has widely been made in theory and computational modeling. Individual molecules are considered to be isolated systems and indeed are often experimentally studied as such (e.g., in gas-phase spectroscopic measurements); even in a condensed-phase molecular system, there is a clear separation into strong covalent interactions within a molecule and much weaker ones that couple molecules. In contrast, materials are extended systems where such a separation is not normally well-defined. This distinction has been reflected in the scope of most atomistic ML-based models reported to date being focused either on materials or on isolated molecules. In both cases, these new methods have achieved a step change in the system size that can be treated with first-principles accuracy and predictive power.

We envision that in the future, the conceptual separation between materials and molecules will be less distinct and ultimately cease to exist, because there is no fundamental requirement for it. Topology-free potentials, which do not depend on any fixed definition of bonds, have become more flexible and accurate by using ML methods and are increasingly able to match the accuracy of traditional bonded force fields. They can therefore reproduce the part of the configuration space that does not involve changes in bonding topology, while simultaneously describing more general configurations, including bond breaking and formation, more accurately than traditional reactive potentials.

In the interest of making atomistic ML models, such as GPR, broadly useful to various communities, further work is needed in terms of protocols and workflows—such that the construction of a new model no longer requires the user to have detailed knowledge of the ML methodology itself. In one extreme, this could be achieved by the on-the-fly fitting schemes that aim to accelerate *ab initio* MD. It may be expected that in the medium term, any such simulation that can generate sufficient data (i.e. a few hundred or a thousand configurations of the full simulated system) with the reference method will benefit from GAP or similar acceleration, as long as it is dependent on reaching *long time* rather than *large length* scales. A critical prerequisite for this is a good understanding of the predicted error or other uncertainty quantification methods, which constitute an active field of research in GPR modeling and in ML more generally. At the other extreme, we envision the use of highly general and flexible GAP models which we call “general-purpose”, in which the development of reference databases becomes a centrally important methodological aspect. We have introduced such models for a number of challenging elemental systems (C, Si, P)—although constructing suitable databases and ML potentials of the same scope for general multicomponent systems with complex phase behavior will be an even larger challenge.

Most GAP models in current use rely on the combination of low-body-order descriptors and SOAP descriptors, with appropriate scaling factors, as described in ref (122). There is active development going on in terms of SOAP and related many-body representations,^51^ which are typically different from the ways that chemists think in terms of bond distances and angles. In particular, the atomic cluster expansion (ACE)^68,417^ is a generalization of SOAP which explicitly keeps the low-body-order terms, which have been so successful in classical force fields—while also remaining computationally efficient up to high body order.

In terms of computational cost, GPR models constitute a middle ground between mathematically simpler (less flexible, cheaper) and more complex and flexible regression methods that are more demanding at least at the fitting stage. Examples of the former are fast linear models such as MTP, SNAP, and the aforementioned ACE, and these are well suited for very large-scale simulations. Regarding the latter, it is envisioned that with sufficient amounts of data, in the future one will be able to construct even “deeper” neural networks, not just of the feed-forward type but including message passing networks, that can capture increasingly subtle features of the target function. It is likely that a range of regression methods will continue to be used, each suited to a particular purpose.

Being a Bayesian method, GPR relies on the specification of a prior, which can be regarded as a bias that we place on the functional space, based on our prior knowledge of the fitting problem. In the case of GAPs, the prior imposes locality and ensures the smoothness of the potential but is otherwise rather “permissive” and does not impart to the model further physical knowledge of specific atomic interactions. Incorporating physics into the form of the potential (while retaining sufficient flexibility) is a development direction which has the potential to reduce the amount of data required in the fitting and improve transferability of GAPs. A concrete example is given by the construction of atomistic regression models for ionic charges based on local environments and the direct inclusion of such properties (which may also include higher-order multipoles) into the fitting of a more accurate force field that explicitly treats long-range electrostatic interactions. Conceptual steps in this direction have been made using neural-network models early on^356^ and also more recently.^331^ One may furthermore think of the on-the-fly learning of other parameters, such as those required for the explicit construction of many-body dispersion corrections rather than learning the latter only implicitly through the data from the reference method.^163^ Such an approach would enable straightforward and routine applications of ab initio MD at levels of theory which so far have been out of reach, even in cases where “only” the many-body dispersion parameters or another part of the computation, instead of the full potential-energy surface, need to be machine-learned.

**Figure 45:**
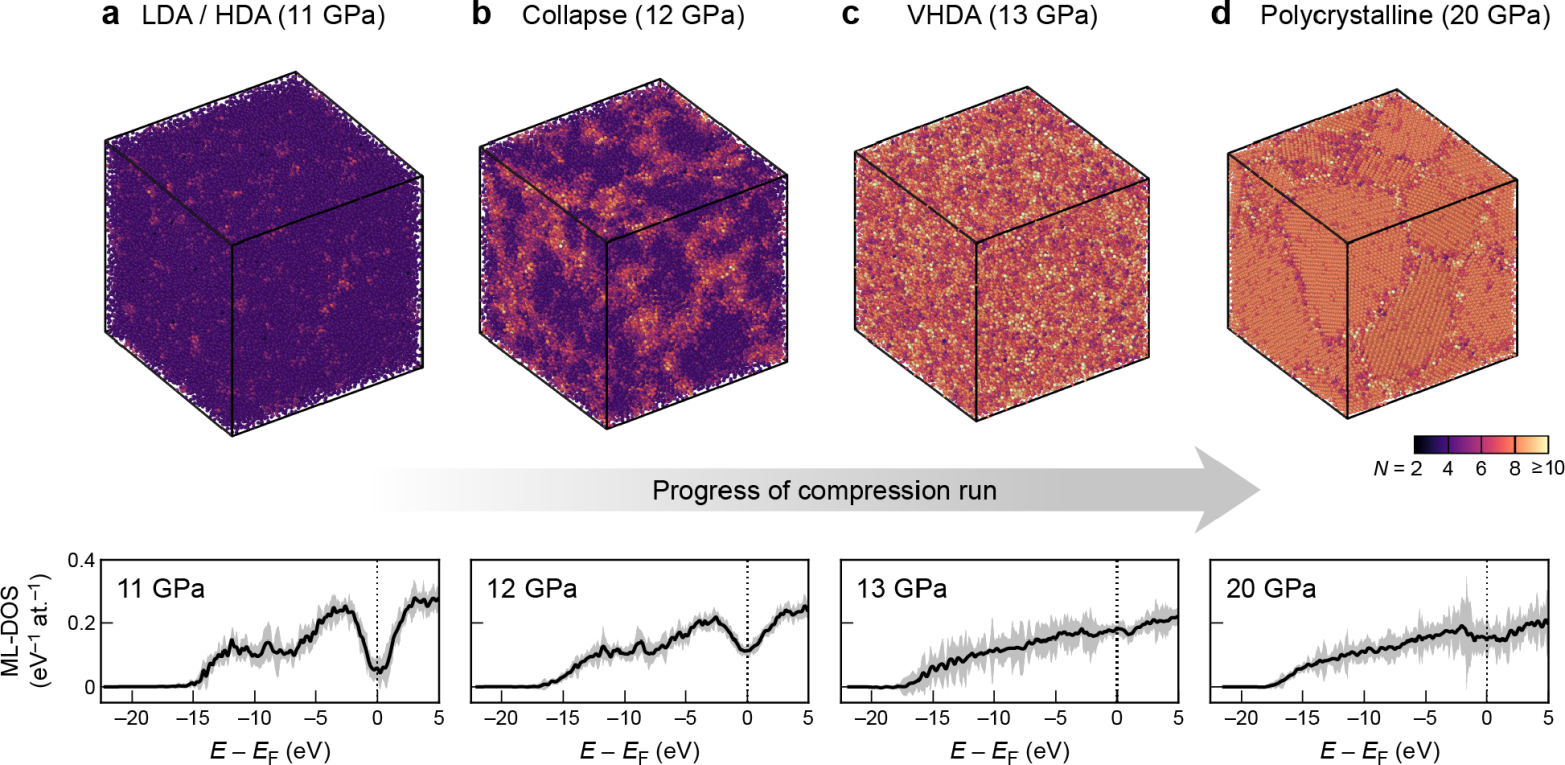
GPR models provide a unified view into structural and electronic properties of complex systems—here exemplified for dense disordered silicon, simulated using a system containing 100,000 atoms. The upper panels show atomic structures, obtained from a GAP-driven molecular-dynamics simulation in which an a-Si sample was compressed from ambient pressure to 20 GPa. The simulation revealed a series of structural transitions, from a low-density amorphous (LDA)/high-density amorphous (HDA) phase persisting up to about 11 GPa, through a distinct very-high-density amorphous (VHDA) phase with much higher coordination numbers at about 13 GPa, to the eventual formation of a polycrystalline structure with simple hexagonal grains. The lower panels show the corresponding electronic structures as described by the machine-learned densities of states (ML-DOS), which were also obtained in a GPR framework. Adapted from ref (164), where more details may be found.

Will ML models *replace* electronic-structure calculations and empirical force fields? We do not think so. The former will always be required to create reference data, while empirical force fields, being orders of magnitude faster than ML models, will continue to be used. Hence, rather than being a replacement, ML models can serve as the necessary “glue” that ties together modeling on different length scales in a systematic manner, thus enabling the program of first-principles modeling to be carried beyond electronic-structure calculations. We are now in the position to create models which combine very large-scale (10 nm and beyond) simulation with the accurate prediction of relevant atomistic properties. Recent work exemplified this synergy, combining the prediction of atomic forces (giving access to MD simulations for a 100,000-atom system, see Figure 45) with an ML model for the electronic density of states, together affording insight into the structural *and* electronic transitions in pressurized disordered silicon.^164^ Accurate prediction of ground-state energetics together with those of properties related to electronic, optical, or magnetic excitations is set to remove a critical roadblock.

In conclusion, data-driven techniques are poised to become an integral part of the molecular and materials modeling toolkit, helping to solve challenging scientific problems in years to come. We look forward to the time when machine-learning methods will have truly arrived in the community and their use in the context of atomic-scale simulation will be so natural and ubiquitous that it will not even merit special emphasis.

## Notes

Data Availability. Python (Jupyter) notebooks allowing reproduction of several of the plots shown in the present paper, as well as other supporting material, will be made available online at https://github.com/gabor1/chemrev-gpr.
